# Hierarchical landform delineation for the habitats of biological communities on the Korean Peninsula

**DOI:** 10.1371/journal.pone.0259651

**Published:** 2021-11-05

**Authors:** Nam Shin Kim, Jin Yeol Cha, Chi Hong Lim

**Affiliations:** Division of Ecological Survey Research, National Institute of Ecology, Seocheon, Chungnam, Korea; Duy Tan University, VIET NAM

## Abstract

Landforms determine the locations of particular biological communities based on their components and spatial positions. This study hierarchically classified the topographic spaces serving as habitats for biological communities in the Korean Peninsula and established the habitat types that occur on the classified landform types. We classified landform types by applying cell-based modeling, map algebra, and spatial query techniques to spatial data, including digital elevation model (DEM), Sentinel 2 image, land use, and field survey data to model their ecological characteristics. Landforms were classified into four categories (designated Category 1 through 4) according to their spatial scale based on topographical characteristics such as mountains, plains, alluvial landforms, coastal landforms, islands, and special areas (Baekdudaegan, DMZ), which are found throughout the Korean Peninsula. The landforms of the Korean Peninsula were classified into 47 subcategories in Category 1, 16 in Category 2, 36 in Category 3, and 63 in Category 4. There were 62 main types of habitats that were classified based on their topographic spatial units, and there were 437 types of sub-habitats, for which soil weathering, biodiversity, and geodiversity were combined with the main habitat types. When factor analysis was conducted for the environmental factors used to determine the main and sub-habitats, the first primary components were temperature-related factors, followed by biodiversity, geodiversity, aspect, and slope. When the indicator species were analyzed by habitat type, indicator species diversity was high in Jeju Province, Gangwon Province, and Gaema Plateau. Based on these results, landform elements for species habit conservation were assigned conservation values and classified into (I) absolute conservation areas, (II) transition areas, and (III) areas for coexistence with humans. Topographic spaces are being degraded as biological habitats as a result of climate change and human development; our proposed classifications can be applied to the conservation of landforms and biodiversity.

## Introduction

Earth is composed of various types of geomorphological landscapes. Geomorphological landscapes are formed by morphogenic and pedogenic processes [1–3]. Although they may be perceived as static because the timescales over which landforms change are much longer than those of biological elements, landforms are constantly being formed and altered over geological time through weathering, erosion, and sedimentation [4, 5]. Landforms are not only formed by abiotic processes, but also by the eco-dynamic equilibria among their structural (soil, landforms, and geology), biological (plant, animals, and microorganisms), and circular components (wind, water) [6].

The geomorphological landscape underlying a terrestrial ecosystem shapes the abiotic environment and determines which organisms can live there. Damage to landforms caused by continuously increasing anthropogenic land use and climate change is disrupting ecosystems and threatening the survival of species. The importance of habitat conservation is increasingly being recognized, and attempts to introduce geomorphological knowledge into ecological research are gradually expanding [7].

The Korean Peninsula contains unique natural environments because of its locational characteristics; as a peninsula located at the eastern edge of the continent of Asia, it experiences both continental and maritime climate characteristics. Moreover, cultural and historical factors, such as the importance and appreciation of the Baekdudaegan mountains in Korean culture and thought [8], the geopolitical specificity of the division of Korea into north and south, and rapid urbanization, combined with its natural environments, shape the unique biogeographic features of the Korean Peninsula. The Korean Peninsula is a region that has small elevational differences and a small spatial scope in which various landforms and biota exist. To establish a framework for the biological conservation of the Korean Peninsula, basic research on its ecosystems with respect to their biogeographic characteristics is necessary.

Therefore, this study classified landforms to identify biological habitat systems at the level of the Korean Peninsula, from broad biota distribution trends to microclimates appropriate for biological communities [9]. To that end, topographic spaces were hierarchically classified into biological habitats using satellite images, geology, digital topographic map, weather data, and biological survey data, and a digital elevation model (DEM) was generally used for landform classification. The habitat types were classified based on the classified topographic spaces, and the major indicator species of each habitat were derived.

## Materials and methods

### Description of study area

**Fig 1:**
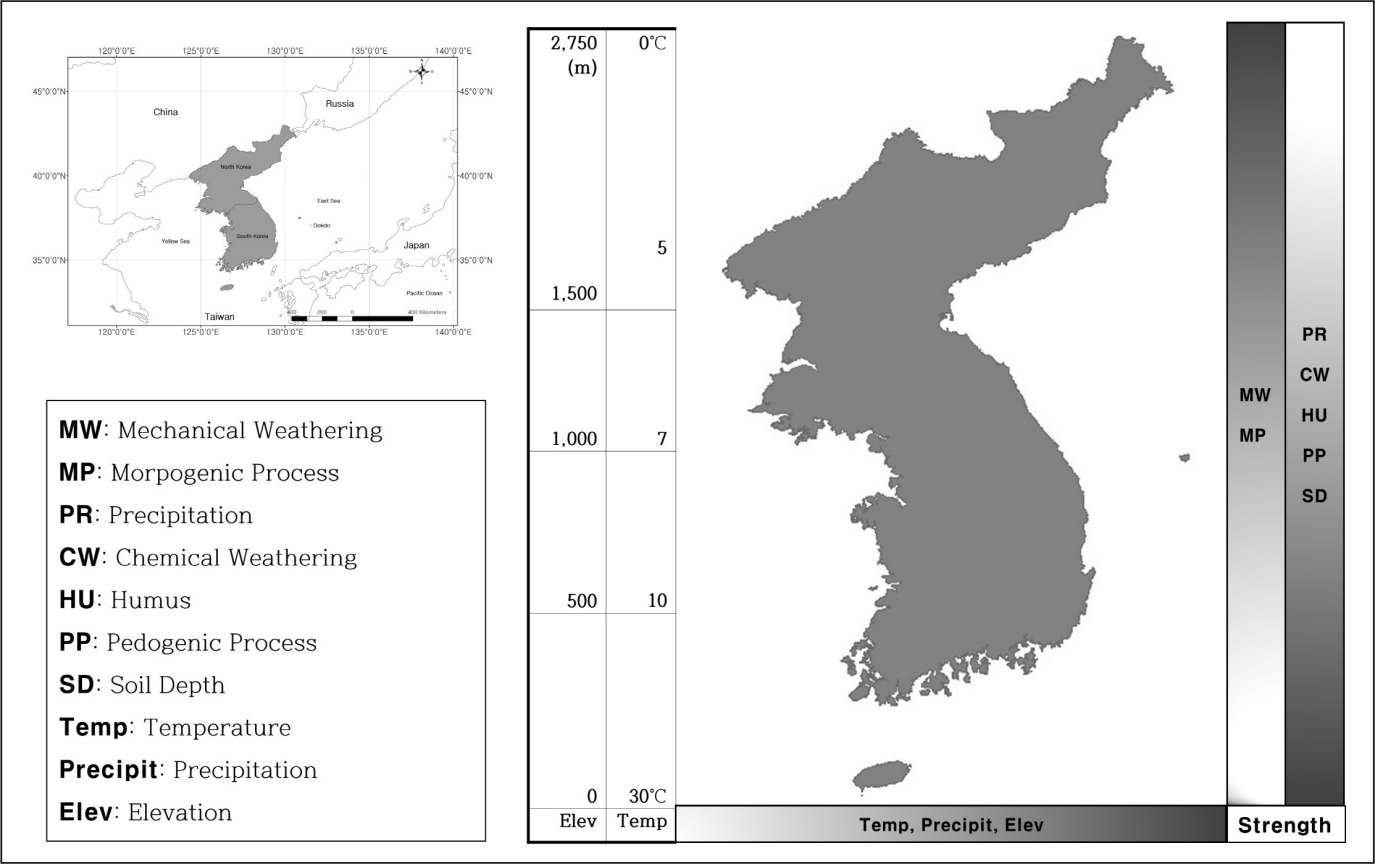
Diagram of geomorphic milieu in Korean Peninsula (base map data source: Https://gadm.org/).

The Korean Peninsula, the target area of this study, spans from 33°05′32″ to 43°00′52.67″ N in latitude and from 124°40′30.77″ to 131°55′45″ E in longitude (Fig 1). Geographically, the Korean Peninsula is located in the easternmost part of the Asian continent in the mid-latitude region of the Northern Hemisphere. Due to these geographic characteristics, it is subject to the simultaneous influences of continental and maritime climates. The annual average temperature is 10–15°C, and the annual average precipitation is 1,000–1,900 mm.

Altitudes on the Korean Peninsula range from 0–2,750 m above sea level. High altitude areas are asymmetrically located to the east and north of the peninsula. The areas have more active morphogenic processes and less humus in their soils. By contrast, environments in the southern region are characterized by higher chemical weathering and pedogenic activities, more rapid plant litter decomposition rates, and as a result, humus-rich soils. Soil depths (to bedrock) vary among soils with different parent materials but tend to be larger among the predominantly granitic soils of the south.

The forests of the Korean Peninsula are dominated by deciduous broadleaved forests, and coniferous forests have developed mainly in the lowlands following afforestation. Furthermore, there are evergreen broadleaved forests around the southern coast and evergreen coniferous forests in some subalpine areas. Specific habitat environments formed by topographic and geological conditions, such as algific talus slope and limestone field, serve as major habitats for the unique species of the Korean Peninsula [10, 11] (Table 1). The islands on the southwest coast, which were geographically isolated by a sea level rise in the 4th Cenozoic era, provide a habitat for terrestrial endangered species as well as rare and endangered migratory birds [12, 13].

**Table 1:** Major ecosystems and biota of the Korean Peninsula (sources: *National Ecosystem Survey* and *DPRK Flora and Fauna of Coreana*).

Ecosystems	Biota
Mountains	*Panthera tigris* altaica (TEMMINCK), *Panthera pardus*, *Ledum palustre* var. maximum, *Betula microphylla* var. *coreana*
Disjunct Distribution Mountain	*Abies koreana*, *Vaccinium vitis-idaea* L., *Diapensia lapponica* var. obovata F. Schmidt
Algific talus slope (wind hole)	*Diplazium sibiricum* (Turcz. ex Kunze) Sa Kurata, *Lycopodium selago* L., *Cystopteris fragilis* (L.) Bernh.
Limestone fields	*Mitella nuda* L., *Morus mongolica* (Bureau) C. K. Schneid, *Arenaria stricta* var. *uliginosa* (Schleicher ex Lam. & DC.) B. Boivin
Wetlands and Bogs	*Astilbe koreana* Nakai., *Trientalis europaea* subsp. *arctica* (Fisch. ex Hook.) Hultén, *Falco subbuteo*, *Naemorphedus caudatus*, *Pelophylax chosenicus*, *Ranunculus kazusensis* Makino, *Ranunculus kazusensis*
Tidal flat	*Suaeda glauca* (Bunge) Bunge, *Suaeda maritima* (Linnaeus) Dumortier, *Uca lactea* subsp. *lactea*
Sand dune	*Lathyrus japonicus* Willd, *Salsola komarovii* Iljin, *Vitex rotundifolia* L. f.
Ridges and south-facing slopes	*Pinus densiflora* Siebold & Zucc, *Abies holophylla* Maxim., *Taxus cuspidate* Siebold & Zucc.
Fluvial landform	*Acheilognathus somjinensis*, *Iksookimia koreensis*, *Pseudobagrus brevicorpus, Phragmites japonica Steud., Typha orientalis* C.Presl, *Salix gracilistyla* Miq.
Lagoon	*Menyanthes trifoliata*, *Iris laevigata*, *Euryale ferox* Salisbury
Plateau	*Tetrao tetrix*, *Panthera pardus*, *Sorbaria kirilowii* (Regel & Tiling) *Maxim*.
Island	*Kichulchoia brevifasciata*, *Thalasseus bernsteini*, *Campanula takesimana*, *Fagus engleriana* Seemen.

### Data acquisition and preprocessing

For the DEM, a shuttle radar topography mission (SRTM) DEM with 30-m resolution was downloaded and used (https://earthexplorer.usgs.gov/). Since the DEM was produced by image analysis, not by terrestrial measurements, it includes errors such as contour line breaks and twists due to the irregularities of sinks, peaks, and elevation points [14, 15]. Thus, 10 × 10 low pass filtering correction was performed for the DEM data [16]. The corrected DEM was resampled to 60-m resolution to classify geomorphological landscapes of medium or larger sizes and resampled to 30-m resolution for small-scale landform classification.

The digital geology map was downloaded from the website of the Korea Institute of Geoscience and Mineral Resources (https://data.kigam.re.kr/). It was used to extract the distributions of geomorphological landscapes, such as mountains, riverbeds, riverbed structures, sand beaches, and dunes, that are formed by weathering, erosion, and uplift due to the characteristics of bedrocks and faults, as well as the distributions of river sediments, coastal sand beaches and tidal flats. Since the digital geology map is a vector map, it was transformed into raster maps of 60- and 30-m spatial resolutions according to the DEM resolutions.

The digital topographic map (DTM) constructed by the Korea National Geographic Information Institute was downloaded from their website (http://map.ngii.go.kr) and used for the classification of small habitats in South Korea, such as riparian wetlands, dolines, small wetlands, shoals, and dunes [17]. A scale of 1:5,000 was used for the DTM, and a DEM with a 5-m resolution was produced by extracting contour lines at 5-m intervals. After performing the first small landform classification, the landforms were classified by comparing them with Google Earth images. In addition, to produce a landform distribution map for the small wetlands, shoals, and dunes in North Korea, for which the DTM cannot be used, DEM analysis was conducted, and the Google Earth images were deciphered visually.

To classify sedimentary terrain elements, such as lagoons, tidelands, wetlands, and sand beaches, that cannot be extracted from the DEM, we used Sentinel 2 satellite images with 10-m resolution that were captured in May and June 2019, a land use map that was constructed in 2018 by the Ministry of Environment, and Google Earth images.

Mountain marshes and algific talus slopes are very small habitats and cannot be checked by images because of interference from the surrounding forests. Thus, mountain marshes were classified using the location information of the landforms found in the national ecosystem survey performed by the Korea National Institute of Ecology [18]. For the classification of small landforms with unusual habitats, such as algific talus slopes [11, 19, 20], the locations and species information in national ecosystem survey data and papers were used [11].

For the soil weathering analysis, the degree of soil weathering was classified by the Peltier method for the temperature and precipitation data downloaded from WorldClim (https://www.worldclim.org/) [21, 22].

The flora location information of the northern and southern parts of the Korean Peninsula was built by integrating approximately 1.6 million survey results from the Korea National Institute of Ecology [18] and North Korea [23–36] based on the national list of indigenous species of Korea [37]. We unified the form of the collected information and built a database based on the scientific name. The database was converted into spatial information of the geodetic longitude and latitude projection (EPSG 4326, WGS84 ellipsoid).

To derive the biodiversity and geodiversity of the Korean Peninsula, the Korean Peninsula was divided into 2000 m × 2000 m grids, and the Shannon’s diversity index was calculated for the biological species and landforms recorded in each grid. To classify the ecoregion types, the biological species and landforms were rearranged by overlaying polygon administrative districts map(gun-unit administrative district level) and the raster data of Shannon’s diversity index [2, 7, 38–40].

### Landform classification

In order to classify landforms as biological habitats, we considered the advantages and limitations of the landform classification through the DEM, the need for supplementary data, and previously studied geomorphological expertise.

The DEM, which contains surface elevation data, does not include the information on all landforms on Earth. The mountains, plains, terraces, ridges, slopes, and watersheds can be classified by applying statistical classification methods (e.g., clustering, PCA) to the DEM elevation data. However, this can be only applied to the overall categorization in small sale classifications of the Earth or continent units and is not appropriate for classifying topographic spaces into biological habitats. Furthermore, the DEM lacks landform data for wetlands, riverbeds, lagoons, sand beaches, shoals, and blockfields, which are important biological habitats. Thus, this study used supplementary data, including satellite images, land use maps, and field survey data, to classify landforms as biological habitats.

Even though the result of the primary landform classification can be derived according to the two conditions described above, the actual existence of geomorphological landscapes must be verified. This requires expertise, complemented by a perspective on the interpretation of the geomorphological landscapes. To address this issue, we performed space calculations iteratively, adjusting the conditions based on our judgments regarding the existence and geomorphology of the landforms in the extracted data.

**Fig 2:**
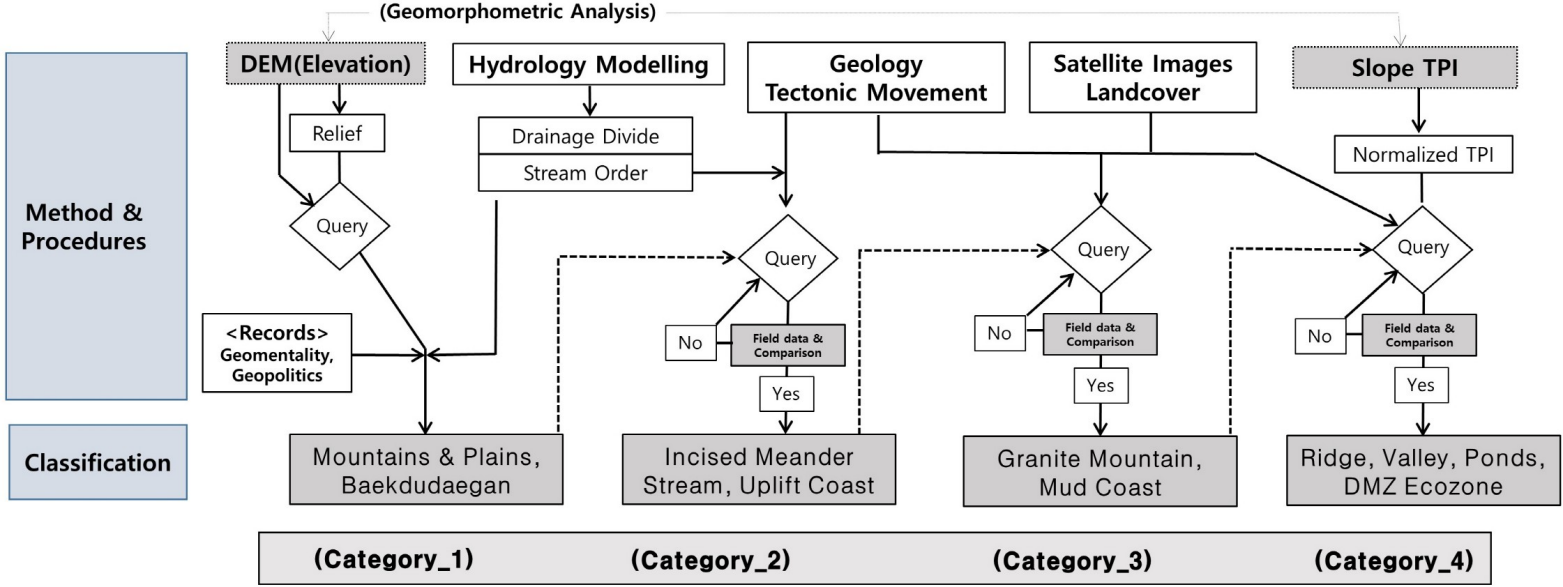
Flowchart showing the overall process of landform delineation.

The overall research procedure and analysis methods are shown in Fig 2. According to this procedure, the metrological landform classification was performed through map algebra, spatial query, supplementary data, and geomorphological review (for detailed information about the classification criteria, see S2 File).

The mountains, plains, basins, flats (plateaus), hills, undulating landforms, terraces, sloping landforms, and gentle slopes (ranging from large to small landforms) in the Korean Peninsula were classified by traditional geomorphological relief analysis. In geomorphology, relief is used in the formation and classification of landforms by calculating the elevation difference between the maximum and minimum points in a unit area created by the weathering and erosion of the original landforms. The relief data has continuous values above 0. Theoretically, a large relief value corresponds more closely to a mountain and a relief value of 0 corresponds to a plain, flat (plateau), terrace, mountain plateau, or tableland. Various landforms can be classified by dividing the continuous values of the relief into discrete sections. In the DEM, the relief is calculated by the square root of the square of the maximum elevation value minus the square of the minimum elevation value in a unit area, as follows:

$$Relief=\sqrt[{2}]{abs(square(\max)-square(\min)))}$$

Relief analysis provides useful information for analyzing landform development and can be used to classify major landform elements, but it needs to be improved because concave and convex landforms cannot be sufficiently explained by relief. The basic principle for extracting concave and convex landforms from the DEM is to calculate the relative value of geomorphic surface based on the average elevation. If the DEM minus the average elevation of a landform is a positive number, the landform is classified as a convex landform, and if it is a negative number, the landform is classified as a concave landform.

For analysis of the sloping landforms, the landform classification technique developed by Zimmermann [41] to determine the topographic position index (TPI) was calculated. The TPI compares the elevation of each cell in a DEM to the mean elevation of a specified neighborhood around that cell [42]:

ZimmermanTPI=DEM−Mean

where *DEM* is the elevation of each cell and *Mean* is the mean elevation for the adjacent cell. In addition, a normalized surface to normalize the roughness of the TPI was calculated. Normalized surface measures the topographic position of local relief normalized to local surface roughness [43]:

$$Normalized\;Surface=\frac{ZimmermannTPI-ZimmermannTPI_{mean}}{ZimmermannTPI_{SD}}$$

where *ZimmermannTPI*_*mean*_ is the mean of *ZimmermannTPI*, and *ZimmermannTPI*_*sd*_ is the standard deviation of *ZimmermannTPI*. Zimmermann’s slope landform classification is appropriate for the classification of four landforms (ridge, toe slope, slope, and valley), unlike many slope landform classifications suggested by other TPI analysis methods. Many slope landform classification methods are based on measured values, rather than landform classification from a geomorphologic perspective, and have limitations for classifying landforms with respect to their potential as biological habitats.

Spatial information for the watershed and linear stream systems was extracted from the DEM using the hydrological modeling tool in ArcGIS 10.1. The watershed and stream networks were extracted as follows. The Fill function was applied to remove sinks and peaks, which can affect the extraction of drainage basins. Next, the flow values in eight directions on the slopes and ridges were calculated using the Flowdirection function. The cumulative flow value was calculated by applying the Flowaccumulation function using the flow values in eight directions. Lastly, the watershed and stream networks were extracted by applying the cumulative flow value as the threshold.

To construct polygonal stream system spatial information, the watershed information was extracted from the land use map for South Korea. For North Korea, it was extracted by synthesizing the regions with values ≤ 0 for the normalized difference vegetation index (NDVI) derived from the Sentinel 2 images the supervised classification of the Sentinel 2 images.

The lake and lagoon landforms were classified from satellite images and land use maps. Furthermore, wetlands formed by sedimentation on the bed of watersheds that flow into artificial or natural lakes, which are difficult to identify through satellite images and land use maps, were extracted using the following method. The lakes were clipped from the water network in the order in which they were extracted during hydrological modeling, and the nodes of the lake boundaries were extracted from the clipped watersheds. After extracting information about the start and end points of the extracted nodes, the slopes of the end points corresponding to the inflow points were calculated. Then the areas in watersheds with slopes of ≤ 5 degrees that were within 200 m of a lake boundary were first selected as candidate wetlands. Finally, the areas whose NDVI values were ≥ -0.2 and ≤ 0.3 were extracted from the Sentinel 2 images and overlapped with the candidate areas; whether they were wetlands or not was verified through the Google Earth images. For areas that needed improvements, their boundaries were created by headup digitizing.

For the Baekdudaegan, based on the watersheds and stream systems extracted by specifying the threshold of 2,000 for hydrological modeling, the first order streams and the watershed ridges that border them were extracted. Next, the boundaries of the Baekdudaegan were established by referring to the history book “Daegan” by San Gyoeng Pyo.

### Statistical analysis

Factor analysis was conducted for each main and sub-habitat to analyze the main variables influencing the main and sub-habitats, which are topographic spatial units, as ecological areas. Eleven environmental parameters were used in the factor analysis: mean elevation (elev), maximum elevation (elevmax), precipitation (precipt), temperature (temper), biodiversity (biodiv), geodiversity (geodiversity: geodiv), coldness index (cold), continentality (cont), warm index (warm), slope, and aspect. Among these parameters, mean elevation, maximum elevation, and precipitation were analyzed by reverse scoring because they have negative correlations with other parameters and large deviations in their values; they may be calculated as negative scores as a result of principal component rotation. Based on the results of the analysis, the effective factors whose values were ≥ 1 were selected, and orthogonal rotation of the varimax method was performed to identify correlations between the factors.

### Indicator species analysis

To confirm the differences in the composition of plant species according to the habitat type, indicator species by main habitat type were analyzed using plant information of 929,373 individuals corresponding to 268 species. The analysis of indicator species is used to assess the representativeness of biological species for a specific group [44]. In this study, the indicator species for the Category 1 habitats were extracted using the presence/absence data for the plant species that appeared in each sub-habitat spatial unit. For the analysis of indicator species, the ‘multipatt’ function in the ‘indicspecies’ package of R (https://cran.r-project.org/bin/windows/base/, 4.0.2) was used [45].

## Results

### Landform classification

**Fig 3:**
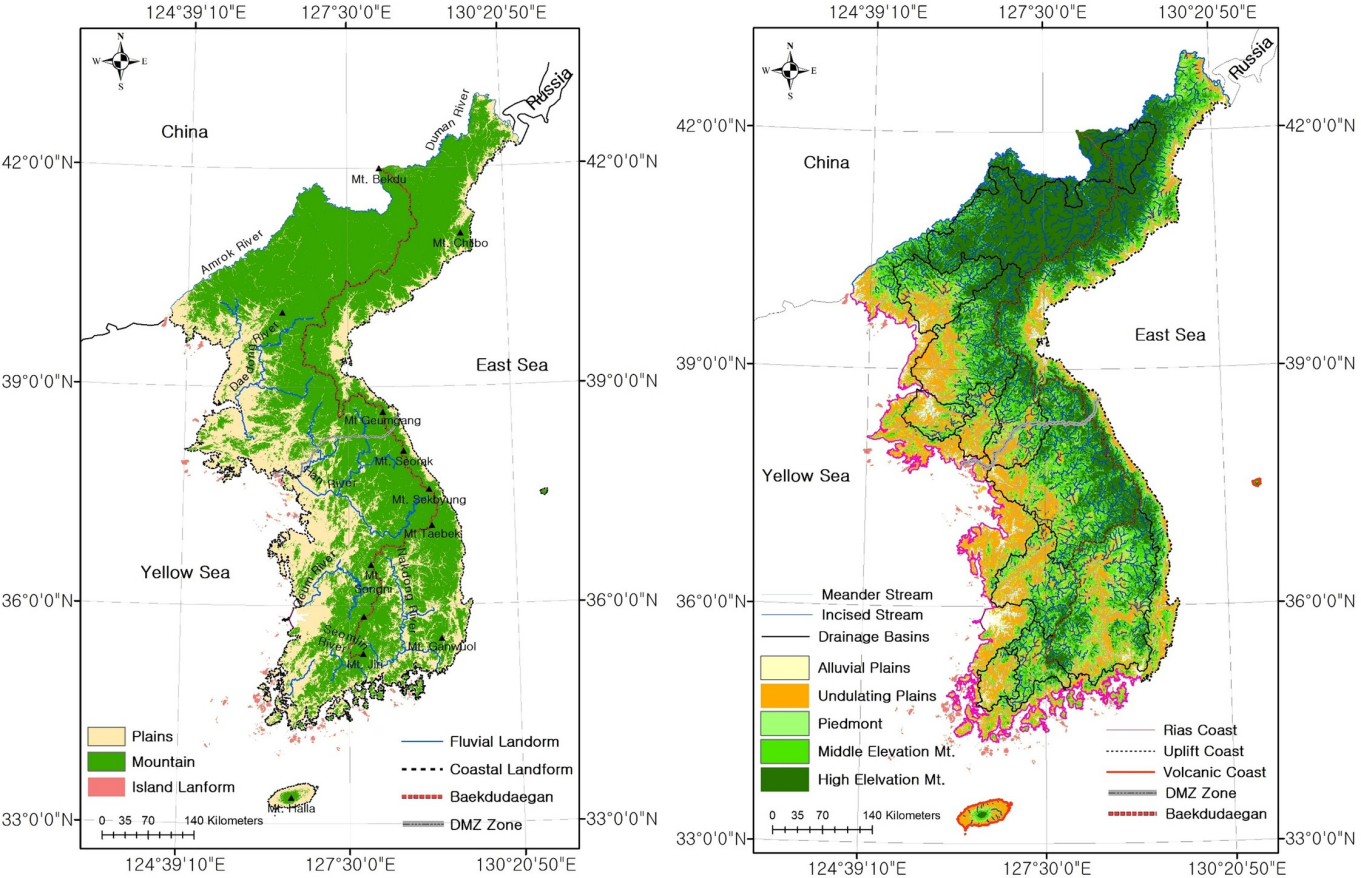
Result of habitat categorization by classification scale (left: Category 1, right: Category 2; base map data source: Https://gadm.org/).

**Fig 4:**
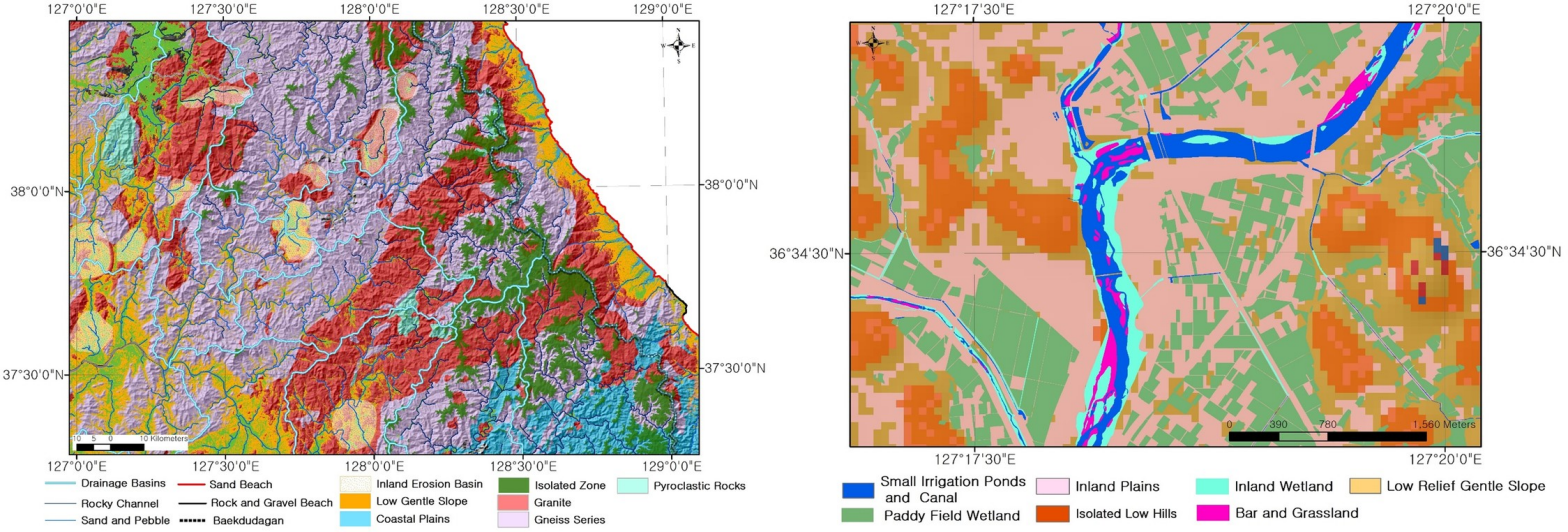
**Result of habitat categorization by classification scale (left: Category 3, right: Category 4; base map data source: Https://gadm.org/).** Categories 3 and 4 are case maps. For detailed maps, see S3 File.

To categorize the habitats into topographic spatial unit, the landforms were classified into Category 1 (1:5,000,000 scale), Category 2 (1:1,000,000), Category 3 (1:50,000 to 1:25,000), and Category 4 (under 1:5,000) as shown in Table 2 (Figs 3 and 4).

**Table 2:** Hierarchical landform classifications by scale.

Category 1 (1:5,000,000)	Category 2 (1:1,000,000)	Category 3 (1: 50,000 ~ 25,000)	Category 4 (Under 1:5,000)
Mountains	High Elevation Mt.	Mt. Granite	Magma plateau
		Mt. Gneiss Series	Highland flat
		Mt. Tertiary Layer	Karst basin
	Middle Elevation Mt.	Mt. Limestone	Karst flat
		Mt. Pyroclast	Wetland Mt.
		Volcanic Mt.	Algific talus slope
			Piedmont
	Piedmont	Lava plateau	Ridge
		High flat	Valley
		Limestone basin	Ridge saddle
	Drainage divide	Isolated mountain	Rock block
		Drainage divide	Mountain cliff
			Creator
		Water and lake	Mountain bog
	Water and lake	Wetland in lake	Doline wetland
		Inland erosion basin	Drainage divide
		Caldera basin	Water and lake
			Wetland in lake
Plains	Alluvial plains	Coastal plains	Bar and grassland
			Waterway and wetland
		Inland plains	Small irrigation ponds and canals
	Undulating hills	Delta plains	Small inland plains
			Small coastal plains
		Water and lake	Inland wetland
			Isolated low hills
		Wetland in lake	Low relief gentle slope
			Paddy field wetland
		Low gentle slope	Water and lake
			Wetland in lake
Fluvial landform	Incised meander stream	Rocky channel	River terrace
			Alluvial island
		Sand and gravel channel	Riparian wetland
			Riverside wetland
	Meander stream	Silt and mud channel	Braided stream channel
			Bar
		Water and lake	Riparian zone
			Stream and lake
		Wetland in lake	Wetland in lake
			Fluvial cliff
Coastal landform	Uplift coast	Rocky coast	Coastal terrace
			Rocky beach
		Sand coast	Sand beach
			Sand and mud Beach
	Rias coast	Sand and mud Coast	
			Sand and gravel beach
			Tidal flat
		Sand and gravel Coast	Mixed Coast
			Sand dune wetland
		Mud coast	Sand dune
			Salt marsh
	Volcanic coast		Lagoon
		Mixed coast	Coastal cliff
			Headland
Island	Island	Island	Mud beach
			Salt marsh
			Sand beach
			Sand dune
	Volcanic island	Volcanic island	Lagoon
			Tidal flat
			Cliff
			Coastal terrace
Baekdudaegan	Baekdudaegan	Baekdudaegan	Baekdudaegan ecozone
DMZ	DMZ	DMZ	DMZ ecozone

The number of landform types classified by scale were 7, 16, 36, and 63 for Categories 1, 2, 3, and 4, respectively. The Baekdudaegan and DMZ were classified into 7 and 9 zones, respectively, according to their landform, elevation, geological features, and hydrological characteristics (Table 3).

**Table 3:** Number of landform types.

Landforms	Category 1	Category 2	Category 3	Category 4
Mountains	1	5	15	18
Plains	1	2	6	12
Fluvial landform	1	2	5	10
Coastal landform	1	3	6	13
Island	1	2	2	8
Baekdudaegan	1	1	1	7
DMZ	1	1	1	9
Total	7	16	36	77

### Biological classification of landform types

**Fig 5:**
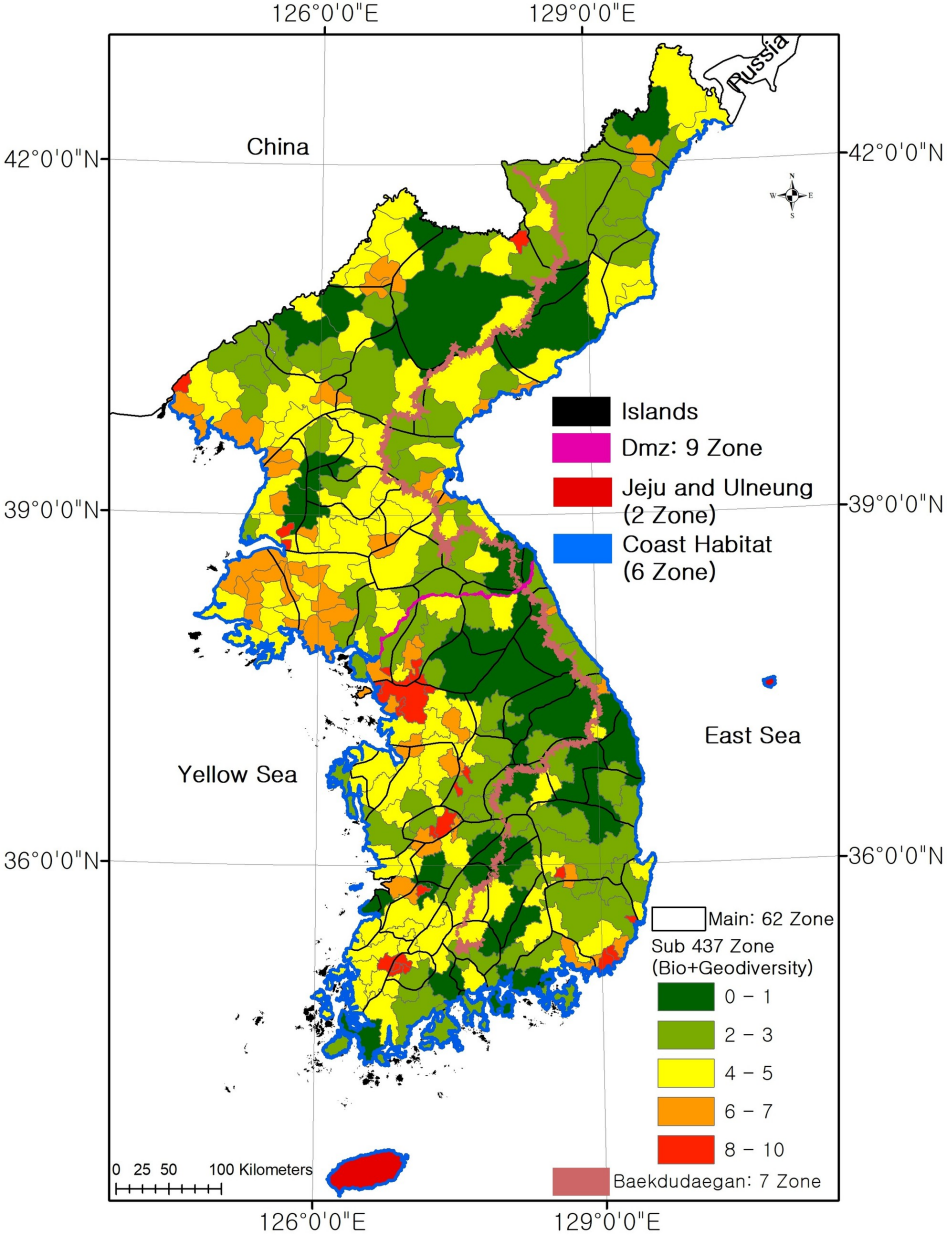
Habitat classification result in Korean Peninsula (base map data source: https://gadm.org/).

The six categories of terrestrial habitats––Baekdudaegan, DMZ, islands, coastal habitats, and two specific island habitats (Jeju Island and Ulleung Island)––were classified into 62 main habitats. These 62 main habitats were classified according to the mountain systems, elevations, and geological features. They were divided into habitats within the northern, central, and southern climate zones (Fig 5).

The sub-habitats, in which each main habitat type is subdivided in consideration of topographical and biological factor, were classified into 437 habitats. In addition, six habitats in Baekdudaegan, nine habitats in the DMZ, six habitats in coastal habitats, two specific island habitats, and other island habitats were additionally classified, resulting in 461 sub-habitats in total. More detailed classifications were not made because individual landforms play the role of habitats with respect to individual organisms and communities (category 4 in S3 File)

### Major factors contributing to landform classification

As a result of analyzing the main habitats, three habitats whose values were ≥ 1 were selected. The communality (h2) was 0.3 or higher for all habitats, indicating communality among the variables. h2 is a definition of common variance that ranges between 0 and 1, and if it is 0.3 or more, the commonality between variables is considered to be high. The total proportion of variance was 46% for rotated component (hereafter, abbreviated as RC) 1, 21% for RC2, and 16% for RC3. Thus, the explanatory power was 83% of the total variance (Table 4).

**Table 4:** Explanatory power of each factor.

Explanation	Main Habitats			Sub-habitats			
	RC1	RC2	RC3	RC1	RC2	RC3	RC4
SS loadings	5.02	2.36	1.73	3.51	2.59	1.56	1.51
Proportion of variance	0.46	0.21	0.16	0.32	0.24	0.14	0.14
Cumulative variance	0.46	0.67	0.83	0.32	0.55	0.70	0.83
Proportion explained	0.55	0.26	0.19	0.38	0.28	0.17	0.16
Cumulative Proportion	0.55	0.81	1	0.38	0.67	0.84	1.00

SS loadings, RC indicate sum of squared of loadings, rotated component, respectively.

The factor analysis result showed that RC1 had high temperature, warm index, and coldness index values, followed by elevation and maximum elevation values (Table 5). RC2 was the main habitat affected by slope, biodiversity, and geodiversity. For the RC3 factors, aspect was high. This result suggests that the main factors for the RC1 ecological area are weather factors, whereas, for RC2 and 3 factors, the local landform elements, slope, and aspect are the factors that influence the biological communities.

**Table 5:** Factor analysis results.

Variables	Main Habitats					Sub-habitats					
	RC1	RC2	RC3	h2	u2	RC1	RC2	RC3	RC4	h2	u2
**elev**	**0.76**	-0.5	-0.21	**0.88**	0.121	**0.63**	**0.63**	-0.19	-0.2	0.86	0.137
**elevmax**	**0.73**	-0.48	-0.19	**0.8**	0.201	0.45	**0.82**	-0.17	-0.19	0.94	0.059
**precipt**	-0.83	-0.36	-0.16	**0.85**	0.152	-0.81	0.15	-0.24	-0.14	0.75	0.249
**temper**	**0.96**	-0.18	-0.19	**0.99**	0.013	**0.89**	0.37	-0.16	-0.12	0.97	0.032
**biodiv**	-0.23	**0.73**	-0.09	**0.59**	0.409	-0.17	-0.29	-0.33	**0.69**	0.7	0.301
**geodiv**	0.18	**0.7**	-0.23	**0.58**	0.424	0.07	-0.03	0.22	**0.86**	0.8	0.2
**cold**	**0.94**	-0.11	-0.27	**0.97**	0.028	**0.92**	0.3	-0.2	-0.09	0.98	0.024
**cont**	-0.57	-0.07	0.68	**0.79**	0.21	-0.72	0.06	0.51	0.1	0.8	0.202
**warm**	**0.95**	-0.25	-0.12	**0.97**	0.029	0.12	-0.91	-0.06	0.03	0.85	0.152
**slope**	-0.33	**0.78**	0.28	**0.79**	0.21	-0.23	-0.59	0.44	0.39	0.75	0.249
**aspect**	-0.03	-0.05	**0.95**	**0.91**	0.091	-0.1	-0.12	**0.87**	0	0.78	0.225

RC, h2, u2 indicate rotated component, communality value between variables, unique variance value, respectively.

In the factor analysis, the sub-habitats were selected by four factors, and these factors explained 84% of the total variance. As a result of the sub-habitat factor analysis, the RC1 factor influenced by temperature, coldness index, and elevation. The RC2 factor influenced by elevation and maximum elevation. The RC3 factor influenced by aspect. The RC4 factor influenced by biodiversity and geodiversity habitats. Furthermore, elevation, biodiversity, geodiversity, coldness index, and aspect were found to influence the sub-habitat factors. The factor analysis results for the main and sub-habitats indicate that the environmental factors of aspect, slope, landform and biodiversity had greater effects on the local biological communities.

### Indicator species for landform types

**Fig 6:**
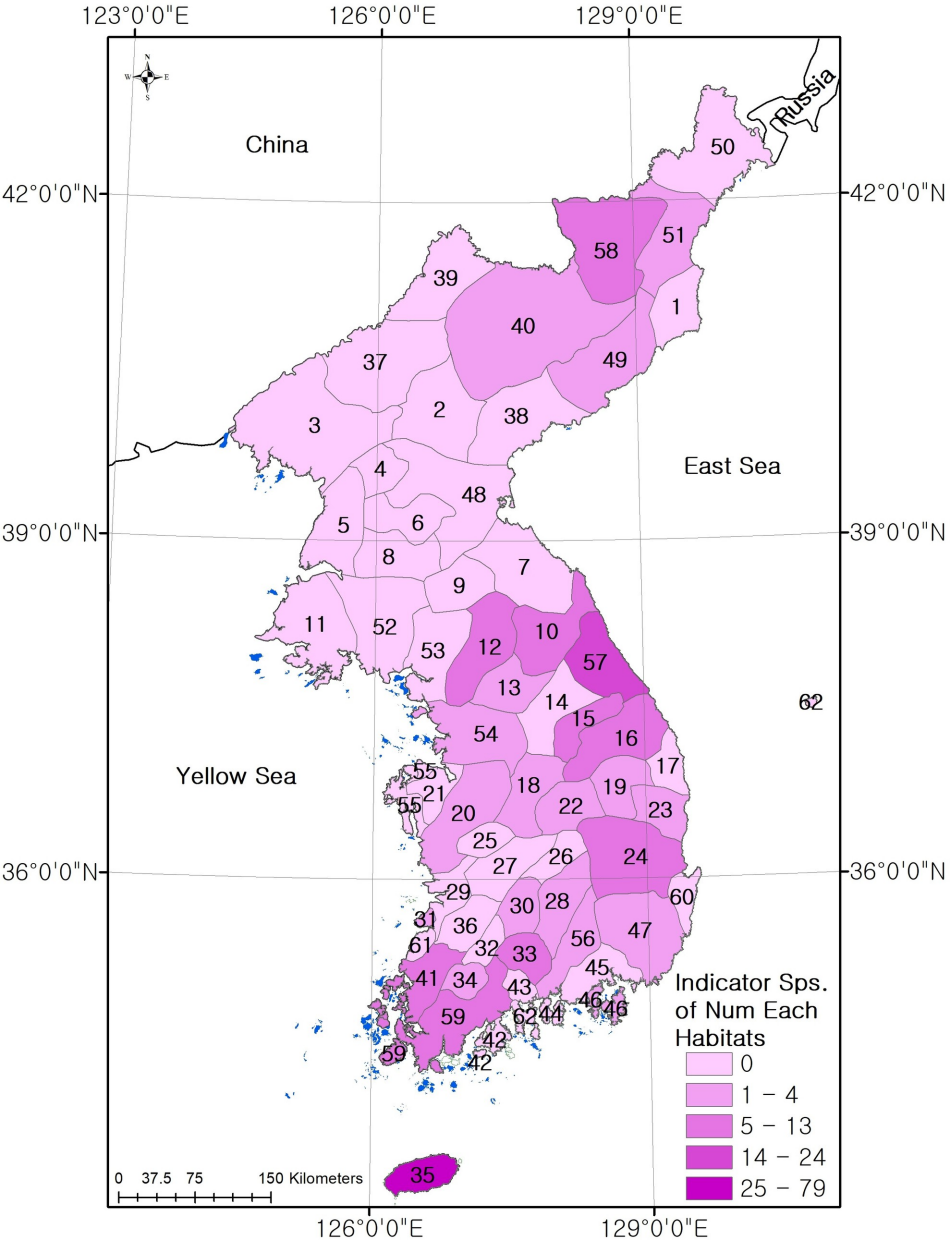
Number of indicator species for each major habitat region in Korean Peninsula (base map data source: Https://gadm.org/).

As a result of the indicator species analysis, statistically significant indicator species were determined for 29 of the 62 major landform types of the habitats in the Korean Peninsula (p<0.05, S4 File). The indicator species by habitat type and the indicator index by species are listed in Table 6. The indicator index and number of indicator species were high in Jeju Province, Gangwon Province, Mt. Baekdu, and Gaema Plateau. There were 79 indicator species representing the Jeju Province, and these included Jeju-endemic species, including *Sasa palmata* (Bean) E.G.Camus, *Peracarpa carnosa* var. *circaeoides* (F.Schmidt ex Miq.) Makino, and *Angelica japonica* A. Gray. There were 24 indicator species for the Gangwon Province, and these included *Veronica kiusiana* var. diamantiaca (Nakai) T. Yamaz., *Androsace cortusaefolia* Nakai, and *Bupleurum euphorbioides* Nakai, which were observed in the Baekdudaegan. There were 13 indicator species for the Mt. Baekdu and Gaema Plateau, including subantarctic species such as *Picea jezoensis* (Siebold & Zucc.) Carrière, *Larix gmelini* var. *principisru-prechtii*, and *Betula microphylla* var. *coreana* (Fig 6).

**Table 6:** Number of indicator species in each habitat.

Habitats	Indicator species number	Habitats	Indicator species number
Group 10	7	Group 34	1
Group 12	8	Group 35	79
Group 13	1	Group 40	2
Group 15	6	Group 41	6
Group 16	9	Group 46	2
Group 18	1	Group 47	4
Group 19	1	Group 49	1
Group 20	1	Group 51	1
Group 22	1	Group 54	1
Group 23	1	Group 56	1
Group 24	5	Group 57	24
Group 28	1	Group 58	12
Group 30	1	Group 59	12
Group 31	1	Group 501	5
Group 33	6		
Total	201 species		

## Discussion

### Considerations for classification method development

Classifying landforms into ecological spatial units can explain trends in the distributions of organisms and habitats across the Korean Peninsula; this differs from academic research on either morphogenic processes or geomorphological causes. In particular, the landform formation process, which is the focus of the field of geomorphology, is an analysis and interpretation of the Cenozoic quaternary ice age and periglacial environments. Thus, it is difficult to generalize it to the relationships with biological habitats.

The classification of landforms as habitats requires a classification system that can explain the presence/absence of animals and plants to the unit of species according to the spatial scale. Organisms may occupy a surface continuously or temporarily, but not every species is present in every topographic space. In particular, plants are more dependent on the habitat environment than animals that can migrate. Therefore, to reflect this, spatial units for landform classification were established in this study.

DEM stores numerical data for continuous heights of landforms and is a representative dataset used to extract landform types. DEM can be used to classify landforms, such as slopes, alluvial landforms, and plains. TPI method of using DEM belongs to the category of slope landform classification because it classifies spatial scopes, ranging from mountain peaks and ridges to the lowest point above sea level. This method is appropriate for classifying sloping landforms, but it has the problem of extracting landforms differently from the actual landforms because the classification result is affected by the resolution and the range of adjacent cells [9, 46]. To address this problem, Jasiewicz and Stepninski [47] used a geomorphon classification method, which analyzes the landform distributions as patterns among the cells and margins, and other studies have used a method of adjusting the clustering threshold [22, 48–55]. However, a pattern classification method using geomorphic surface similarity or statistical classification methods have limitations in classifying landforms created by polygenetic processes because the landforms created by polygenetic processes are rarely distributed in a continuous catena from high to low elevations [1, 2, 56, 57].

These methods tend to be consistent as a whole at the small scale of the national unit, but when it is expanded to the larger scale of regional unit, the resulting landforms may differ from the actual landforms, even though the statistical significance is high. Therefore, to improve the limitations of the geomorphometric landform classification method, a logical method for extracting the habitats and actual landforms from satellite images and land use maps must be applied.

As explained above, when extracting landform elements as vessels for holding biodiversity, the geomorphometric analysis technique cannot reflect the habitats of organisms sufficiently [58–63]. Furthermore, the conditions and data (satellite images, land use map, and various field survey data) for explaining the morphogenic mechanism of the landform extraction process need to be supplemented. The macroscopic distribution trends for vegetation belts and the habitats of animals with large home ranges can be identified by classifying large landforms or the landforms within national units. However, in order to establish the landform basis for a biological habitat system, it must be linked with the species distribution patterns affected by the medium or smaller habitats that can be identified [64]. Landform classification needs to include biodiversity and habitat distribution, for which a methodology that reflects the distribution characteristics of biological factors at a spatial scale is necessary [65].

Landforms can be considered environmental factors that determine the characteristics of species habitats according to the spatial scale and location. Linking landform types with species habitats ranging from broad trends in the national distribution of biota down to the level of small habitats can contribute to our understanding of the spatial distribution patterns of species and biodiversity conservation [16, 57]. Therefore, this study employed the supplementary data (satellite images, land use map, and various field survey data) required for the classification of small and medium-sized habitats in addition to DEM to enable landform classification across multiple habitat scales (Table 7).

**Table 7:** Data types for landform classification.

Data	Data Application	Source	Note
DEM	Mountain, Ridge, Valley, Piedmont, Basin, Drainage divide, River terrace, Coastal terrace, etc.	SRTM 30 m (https://earthexplorer.usgs.gov/)	Digital data
Digital geology map	Differential weathering landform, Habitat	Korea Institute of Geoscience and Mineral Resources (https://data.kigam.re.kr/)	
Digital topographic map	Largescale landform elements	Korea National Geographic Information Institute (http://map.ngii.go.kr/)	
Sentinel 2	Wetland, Lagoon, Delta, Sand dune, Tidal flat, Mt. Meadow, Stream sand bar, Land use, etc.	Santinel2 10 m (https://earthexplorer.usgs.gov/)	
Google Earth image	Karst landforms, Small wetland	Google Earth Pro 7.3.4	
ROK Land Use Map	Land use, Small irrigation pond, Irrigation canal, Paddy wetland, etc.	Korea Ministry of Environment (https://egis.me.go.kr/)	
DPRK Land Use Map	Land use, Small irrigation pond, Irrigation canal, Paddy wetland, etc.	Landsat8 30 m (https://earthexplorer.usgs.gov/)	
WorldClim data	Soil weathering zonation, Warm index, etc.	WorldClim Homepage (https://worldclim.org/)	
Algific talus slope	Algific talus slope and boreal plants information	Korea National Institute of Ecology [18], Published Papers [11, 19, 20]	Field survey data
National Ecosystem Survey	Coastal landforms, Biota, Biodiversity	Korean National Institute of Ecology [18]	
DPRK Biological Species and Distribution in Mt. Baekdu	Plateau landform and biota in Mt. Baekdu	Science & Technology publishing House [24–36]	
DPRK Flora and Fauna of Coreana	Habitat and biota in North Korea	Science & Technology publishing House [24–36]	
National Boundary Map	Coastal landform	Korea National Geographic Information Institute (http://map.ngii.go.kr/)	

Furthermore, general landform classification methods that rely on statistical analysis techniques, such as multivariate statistical analysis and maximum likelihood classification using the DEM, have the disadvantage of coinciding with the actual landform distribution and not being separate from it in the environment of the Korean Peninsula, which is geologically old and is subject to active morphogenic processes. Therefore, in this study, we incorporated research findings as well as previous experience concerning the geomorphological landscapes of the Korean Peninsula to the landform classification.

### Characteristics of landforms and flora in the Korean Peninsula

With respect to mountain landscapes, the bedrocks that have the largest effect on the geomorphological landscapes of the Korean Peninsula are granite and gneiss [57]. Granite mountain landscapes are generally called “mountains with rock block and soil” because of their many rock exposures. In areas where weathering has progressed, soils and piedmonts with a soil depth of more than 10 m are well developed [56]. In contrast, in gneiss mountain landscapes, the surface soil layer covers the entire landscape, and they are called “mountains covered with soil” [66].

The factors required for plant growth are temperature, precipitation, and organic matter. The factors affecting the growth of plants include elevation, soil moisture, and latitude, which affect the habitats and distributions of animals and plants [1, 4, 6]. There is a difference in soil moisture between concave and convex slopes on the surface. Xerophilic vegetation mainly lives on the convex slope, whereas hydrophilic vegetation mainly lives on the concave slope. Due to these surface conditions, coniferous trees are outcompeted by broadleaf trees. In Korean Peninsula, for example, natural maintenance of the *Pinus densiflora* forest was possible only in the restricted sites such as rock outcroppings, weathered rocks, ridgetops, and the sandy or pebble shores of streams where can escape competition with oak forests composing the late successional stage in the region [67]. Therefore, the establishment of strategies for conservation and management of biological habitats should be established in consideration of macro-environmental factors such as temperature, precipitation and topographic factors such as the shape of slopes, existence of water systems. The hierarchical habitat type classification system derived in this study can be used to establish a management strategy according to the spatial scale.

Indicator species were not found for some areas in North Korea except Mt. Baekdu and Gaema Plateau, or for some parts of South Korea because of limitations in the survey method, selection of target species, periodic biota surveys, and scope [24–36]. The reason for this is that natural environment survey collected data randomly for the purpose of investigating biological resources in South Korea, without limiting the target species and specific areas. On the other hand, periodic biological surveys in North Korea were conducted on a limited basis, and a relatively small number of flora data were used for the analysis due to restrictions on information accessibility.

### Proposal for conservation of wildlife habitats

When there is no habitat or the existing habitat is damaged, species become extinct or seek out a new habitat. The factors threatening species habitats include developmental activities by human and climate change. On the Korean Peninsula, disruptions to biological habitats are ongoing because of urban development in South Korea and the expansion of farmlands into forests, wetlands, and tidelands to address food insecurity in North Korea.

When landforms as habitats are damaged by human development or natural disasters, their restoration to their original condition may be impossible or infeasible because of time and cost considerations. Therefore, landforms must be preserved because damage to landforms means semi-permanent loss of species habitats.

The conservation value of landforms as habitats can be classified into absolute conservation areas, transition areas, and areas for coexistence with humans. The absolute conservation area is a threatened area where important biota, such as endangered and endemic species, would disappear without human intervention. Transition areas are at the boundary between absolute conservation area and areas for coexistence with humans, where human development pressure is applied. Therefore, the habitat functions can be maintained to some degree if appropriate replacement and conservation measures are applied when the habitats are developed by humans, although they are required for inhabitation of biota. Transition areas should be conserved because habitat fragmentation and disconnection among suitable habitat patches degrades the habitat function [68, 69]. The area for coexistence with humans is an area where the landforms are damaged and can be developed for human economic activities and residence.

Based on this concept, the conservation values of landform elements for conservation of species habitats were classified into (I) absolute conservation areas, (II) transition areas, and (III) areas for coexistence with humans (Table 8).

**Table 8:** Conservation criteria for each of the landform categories.

Landforms (Habitats)	Human Impact	Geodiversity	Biodiversity	⇒	Conservation Value
Ⅰ	None	High	High	→	Ⅰ
		Low	High	→	Ⅰ
		Low	Low	→	Ⅱ
Ⅰ	Moderate	Middle	Middle	→	Ⅱ
Ⅰ	High	Low	Low	→	Ⅲ
Ⅱ	None	High	High	→	Ⅰ
		Low	High	→	Ⅱ
		Low	Low	→	Ⅲ
Ⅱ	Moderate	Middle	Middle	→	Ⅱ
Ⅱ	High	Low	Low	→	Ⅲ
Ⅲ	None	High	High	→	Ⅰ
Ⅲ	Moderate	Middle	Middle	→	Ⅲ
Ⅲ	High	Low	Low	→	Ⅲ

The absolute conservation areas are (I) topographic spaces that are impossible to recover once damaged, where endangered species and endemic species can live. Some endangered species and endemic species live in transition areas (II), but common species live there in general. The area for coexistence with humans (III) is a topographic space inhabited by species that are ubiquitous across the Korean Peninsula. The areas where anthropogenic land use is ongoing were classified as III because they were already degraded and lost their function as species habitats (S5 File).

## Conclusion

The habitat spaces for wildlife species in the Korean Peninsula were classified, and the ecological areas were categorized into habitats types through the classification of landform elements. To analyze these landforms, they were classified by applying raster modeling, map algebra, and spatial query techniques. The landforms were classified into 7 Category 1 types of mountains, plains, alluvial landforms, coastal landforms, islands, Baekdudaegan, and DMZ, 16 Category 2 types, 36 Category 3 types, and 63 Category 4 types. Based on this landform classification system, the habitat spaces for species were classified into 62 main habitat types and 437 sub-habitat types.

The significance test results showed that temperature, geodiversity, biodiversity, slope angle, and aspect were the main factors that influenced the classification of landform habitat types. The indicator species analysis for each habitat type showed that the number of indicator species was high in Jeju Province, Gangwon Province, and Gaema Plateau.

Topographic spaces as biological habitats are being damaged by climate change and human development. When landforms are damaged, the biota must shift their habitat or face threats to their survival. Therefore, we proposed a conservation value classification system consisting of grades I, II, and III based on our classification of landforms to address landform conservation.

## Supporting information

S1 File Major landforms for habitats in Korean peninsula. (PDF)

S2 File Landform classification procedures by categories. (PDF)

S3 File Landform classification result maps. (PDF)

S4 File Indicator species for each habitats. (PDF)

S5 File Conservation grade of landform for each scale. (PDF)

S1 Data (PDF)

## References

[1] Tricart J, Cailleux A. Introduction to Climatic Geomorphology. Longman; 1972.

[2] Duchaufour R. Pedology: Pedogenesis and classification. Springer Netherlands; 1982. doi: 10.1007/978-94-011-6003-2

[3] Allison RJ. Applied Geomorphology: Theory and Practice. John Wiley & Sons; 2002.

[4] Büdel J. Climatic geomorphology. Princeton, NJ: Princeton University Press; 1982.

[5] Strahler AH, Strahler AN. Modern Physical Geography, 4th Edition. 4th edition. New York: Wiley; 1992.

[6] Tricart J, KiewietdeJonge CJ. Ecogeography and Rural Management: A Contribution to the International Geosphere-Biosphere Programme. Longman Scientific & Technical; 1992.

[7] Lidmar-Bergström K, Olvmo M. Plains, steps, hilly relief and valleys in northern Sweden—review, interpretations and implications for conclusions on Phanerozoic tectonics. Uppsala: Sveriges geologiska undersökning; 2015.

[8] Hyeun J. Hangeul Sangyeongpyo. Seoul, Korea: Peulbit publising house; 2000

[9] Mo Y, Lee DK, Song K, Kim HG, Park SJ. Applying Topographic Classification, Based on the Hydrological Process, to Design Habitat Linkages for Climate Change. Forests. 2017;8: 466. doi: 10.3390/f8120466

[10] Bae K-H, Kim J-S, Cho H-J, Yun C-W, Cho Y-C. Syngeographical Characteristics of Forest Vegetation in Limestone Areas, Mt. Deokhang, Kangwondo. Korean Journal of Environment and Ecology. 2014;28: 161–170. doi: 10.13047/KJEE.2014.28.2.161

[11] Kim J-S, Chung J-M, Kim J-H, Lee W, Lee B-Y, Pak J-H. Floristic study and conservation management strategies of algific talus slopes on the Korean peninsula. Korean J Pl Taxon. 2016;46: 213–246. doi: 10.11110/kjpt.2016.46.2.213

[12] Kong W. Geoecological analysis of the Korea alpine and subalpine plants and lands. J Environ Sci. 1999;11: 243–246.

[13] Kong W. Impact of climate change on arctic-alpine and endemic plants at their global southernmost distributional limit in Korea. Price MF(ed.), Global Change in Mountain Regions. UK: Sapiens Publishing; 2006. pp. 174–175.

[14] Chukwuocha A, Ac-Chukwuocha N, Osuagwu J. Filtering the Shuttle Radar Topographic Mission (SRTM) and the Advanced Spaceborne Thermal Emission and Reflection (ASTER) Digital Elevation Model (GDEM) Data in the Milder Terrains. 11th International Conference of the African Association of Remote Sensing of the Environment, At Kampala, Uganda; 2016.

[15] Wendi D, Liong S-Y, Sun Y, Doan CD. An innovative approach to improve SRTM DEM using multispectral imagery and artificial neural network. Journal of Advances in Modeling Earth Systems. 2016;8: 691–702. 10.1002/2015MS000536

[16] Wang D, Laffan SW, Liu Y, Wu L. Morphometric characterisation of landform from DEMs. International Journal of Geographical Information Science. 2010;24: 305–326. doi: 10.1080/13658810802467969

[17] Kim NS, Lee MB. Landform Classification Using Geomorphometric Analysis. Journal of the Geomorphological Association of Korea. 2004;11: 47–60.

[18] Korea National Institute of Ecology. 4th Nationwide Natural Resources survey report. Seocheon, Korea: Korea National Institute of Ecology; 2019.

[19] Kim J, Chung J, Lee B, Park J. Phytogeographic Gravity and Conservation of the Paleorefugia, Algific Talus in Korea. Proceedings of Korean Society of Forest Science. 2006; 337–339.

[20] Kim J-S, Yun J-H. A Study on the Vegetation Structure of Algific Talus in Korea. Korean Journal of Environment and Ecology. 2013;27: 357–368.

[21] Peltier LC. The Geographic Cycle in Periglacial Regions as It is Related to Climatic Geomorphology. Annals of the Association of American Geographers. 1950;40: 214–236. doi: 10.2307/2561059

[22] Doke A, Pardeshi SD, Pardeshi SS, Das S. Identification of morphogenetic regions and respective geomorphic processes: a GIS approach. Arab J Geosci. 2018;11: 20. doi: 10.1007/s12517-017-3358-5

[23] WorldClim. WorldClim dataset. Available: https://www.worldclim.org/

[24] Im R, Gwak J, Kim H. Flora of coreana(5). Pyongyang, North Korea: Science publishing house; 1975.

[25] Im R. Flora of coreana(6). Pyongyang, North Korea: Science publishing house; 1976.

[26] Im R, Hong G, Kim Y, Gwak J, Lee Y, Hwang H. Flora of coreana(1). Pyongyang, North Korea: Science & Technology publishing house; 1996.

[27] Im R. Flora of coreana(4). Pyongyang, North Korea: Science & Technology publishing house; 1998.

[28] Im R. Flora of coreana(7). Pyongyang, North Korea: Science & Technology publishing house; 1999.

[29] Im R. Flora of coreana(8). Pyongyang, North Korea: Science & Technology publishing house; 2000.

[30] Im R, Lee Y. Flora of coreana(9). Pyongyang, North Korea: Science & Technology publishing house; 2000.

[31] Do B, IM R. Book of the flora(2). Pyongyang, North Korea: Science publishing house; 1976.

[32] Kim L, Gil J. Fauna of coreana(fish 1). Pyongyang, North Korea: Science & Technology publishing house; 2006.

[33] Kim L, Gil J. Fauna of coreana(fish 2). Pyongyang, North Korea: Science & Technology publishing house; 2007.

[34] Kim L, Gil J. Fauna of coreana(fish 3). Pyongyang, North Korea: Science & Technology publishing house; 2008.

[35] Kim L, Han G. Fauna of coreana(amphibian reptile). Pyongyang, North Korea: Science & Technology publishing house; 2009.

[36] Kim L, Gil J. Fauna of coreana(bird 2). Pyongyang, North Korea: Science & Technology publishing house; 2011.

[37] Korea National Institute of Biological Resources. National list of indigenous species of Korean peninsula. Incheon, Korea: Korea National Institute of Biological Resources; 2019.

[38] Bailey RG. Mesoscale: Landform Differentiation. In: Bailey RG, editor. Ecosystem Geography. New York, NY: Springer; 1996. pp. 105–119. doi: 10.1007/978-1-4612-2358-0_8

[39] Bailey RG. Ecoregion-Based Design for Sustainability. New York: Springer-Verlag; 2002. doi: 10.1007/b97607

[40] Pullar D, Low Choy S, Rochester W. Ecoregion classification using a Bayesian approach and model-based clustering. MODSIM05—International Congress on Modelling and Simulation: Advances and Applications for Management and Decision Making, Proceedings. Modelling and Simulation Society of Australia and New Zealand Inc. (MSSANZ); 2005. pp. 1560–1566. Available: https://espace.library.uq.edu.au/view/UQ:7d03259

[41] Zimmermann N. Zimmermann´s Topographic Position Index(TPI). 2000. Available: https://www.wsl.ch/staff/niklaus.zimmermann/progs.html

[42] Weiss A. Topographic position and landforms analysis. Poster presentation presented at: ESRI user conference; 2001; San Diego, CA.

[43] De Reu J, Bourgeois J, Bats M, Zwertvaegher A, Gelorini V, De Smedt P, et al. Application of the topographic position index to heterogeneous landscapes. Geomorphology. 2013;186: 39–49. doi: 10.1016/j.geomorph.2012.12.01544

[44] Peck JE. Multivariate Analysis for Community Ecologists: Step-by-step Using PC-ORD. MjM Software Design; 2010.

[45] Cáceres MD, Jansen F, Dell N. Package ‘indicspecies.’ 2020. Available: https://vegmod.github.io/software/indicspecies

[46] Park SJ, Yu KB. The Optimal Grid Resolution to Interpret the Spatial Structure of Geomorphological Processes over the Landscape. Journal of The Korean Geomorphological Association. 2004;11: 113–136.

[47] Jasiewicz J, Stepinski TF. Geomorphons—a pattern recognition approach to classification and mapping of landforms. Geomorphology. 2013;182: 147–156. doi: 10.1016/j.geomorph.2012.11.005

[48] Saadat H, Bonnell R, Sharifi F, Mehuys G, Namdar M, Ale-Ebrahim S. Landform classification from a digital elevation model and satellite imagery. Geomorphology. 2008;100: 453–464. doi: 10.1016/j.geomorph.2008.01.011

[49] Mokarram M, Roshan G, Negahban S. Landform classification using topography position index (case study: salt dome of Korsia-Darab plain, Iran). Model Earth Syst Environ. 2015;1: 40. doi: 10.1007/s40808-015-0055-9

[50] Karagülle D, Frye C, Sayre R, Breyer S, Aniello P, Vaughan R, et al. Improved Hammond’s landform classification and method for global. 2017 [cited 2 May 2020]. Available: https://webcache.googleusercontent.com/search?q=cache:XvAmWY5paVQJ:https://ir.library.oregonstate.edu/downloads/4x51hk80p+&cd=16&hl=ko&ct=clnk&gl=kr

[51] Piloyan A, Konečný M. Semi-Automated Classification of Landform Elements in Armenia Based on SRTM DEM using K-Means Unsupervised Classification. Quaestiones Geographicae. 2017;36: 93–103. doi: 10.1515/quageo-2017-0007

[52] Camiz S, Poscolieri M. An approach to DEM analysis for landform classification based on local gradients. Earth Sci Inform. 2018;11: 287–305. doi: 10.1007/s12145-018-0337-7

[53] Iwahashi J, Kamiya I, Matsuoka M, Yamazaki D. Global terrain classification using 280 m DEMs: segmentation, clustering, and reclassification. Progress in Earth and Planetary Science. 2018;5: 1. doi: 10.1186/s40645-017-0157-2

[54] Goes ER, Brown CJ, Araújo TC. Geomorphological Classification of the Benthic Structures on a Tropical Continental Shelf. Front Mar Sci. 2019;6. doi: 10.3389/fmars.2019.00047

[55] Zhao K, Deng Z. Landform classification for community siting: A case study in Quxian county, China. J Mt Sci. 2015;12: 1025–1037. doi: 10.1007/s11629-014-3069-2

[56] Oh KS. Origin of Banded Bt Horizons in Sandy Deposits. The Korean journal of quaternary research. 1989;3: 35–45.

[57] Oh KS. Methodological approach for the large scale mapping on morpho-pedological milieu in Korea. Journal of the Geomorphological Association of Korea. 1996;3: 1–27.

[58] Pennock DJ, Zebarth BJ, De Jong E. Landform classification and soil distribution in hummocky terrain, Saskatchewan, Canada. Geoderma. 1987;40: 297–315. doi: 10.1016/0016-7061(87)90040-1

[59] Meybeck M, Green P, Vörösmarty C. A New Typology for Mountains and Other Relief Classes: An Application to Global Continental Water Resources and Population Distribution. Mountain Research and Development. 2001;21: 34–45.

[60] Kim NS, Lee MB. Landform Classification Using Geomorphometric Analysis. Journal of the Geomorphological Association of Korea. 2004;11: 47–60.

[61] Evans D, Williard K, Schoonover J. Comparison of Terrain Indices and Landform Classification Procedures in Low-Relief Agricultural Fields. Journal of Geospatial Applications in Natural Resources. 2016;1: 1–17.

[62] Jiang L, Ling D, Zhao M, Wang C, Liang Q, Liu K. Effective Identification of Terrain Positions from Gridded DEM Data Using Multimodal Classification Integration. ISPRS International Journal of Geo-Information. 2018;7: 443. doi: 10.3390/ijgi7110443

[63] Garcia GPB, Grohmann CH. DEM-based geomorphological mapping and landforms characterization of a tropical karst environment in southeastern Brazil. Journal of South American Earth Sciences. 2019;93: 14–22. doi: 10.1016/j.jsames.2019.04.013

[64] Wright RG, Murray MP, Merrill T. Ecoregions as a level of ecological analysis. Biological Conservation. 1998;86: 207–213. doi: 10.1016/S0006-3207(98)00002-0

[65] Mokarram M, Sathyamoorthy D. Relationship between landform classification and vegetation (case study: southwest of Fars province, Iran). Open Geosciences. 2016;8: 302–309. doi: 10.1515/geo-2016-0027

[66] Oh KS. Cryogenic structures in superficial formation and associated periglacial morpho-climatic milieu in Korean Peninsula. Journal of The Korean Geomorphological Association. 2006;13: 1–17.

[67] Lee CS, Chun YM, Lee H, Pi JH, Lim CH. Establishment, Regeneration, and Succession of Korean Red Pine (*Pinus densiflora* S. et Z.) Forest in Korea. Conifers. IntechOpen; 2018. doi: 10.5772/intechopen.80236

[68] Liu J, Wilson M, Hu G, Liu J, Wu J, Yu M. How does habitat fragmentation affect the biodiversity and ecosystem functioning relationship? Landscape Ecol. 2018;33: 341–352. doi: 10.1007/s10980-018-0620-5

[69] Wilson MC, Chen X-Y, Corlett RT, Didham RK, Ding P, Holt RD, et al. Habitat fragmentation and biodiversity conservation: key findings and future challenges. Landscape Ecol. 2016;31: 219–227. doi: 10.1007/s10980-015-0312-3

