# Supplementary material for: Hierarchical landform delineation for the habitats of biological communities on the Korean Peninsula
Source: PLoS One. 2021 Nov 5;16(11):e0259651. doi: 10.1371/journal.pone.0259651 (PMC8570509; doi:10.1371/journal.pone.0259651)
Supplement: S1 File — (PDF) [file pone.0259651.s001.pdf]

**S1**. Major landforms for habitats in Korean Peninsular.

| Landforms | Major Types | |
|---|---|---|
| mountains | 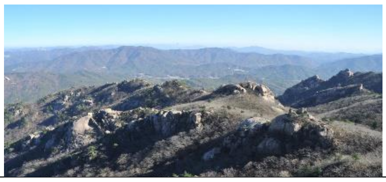 Granite Mountain | 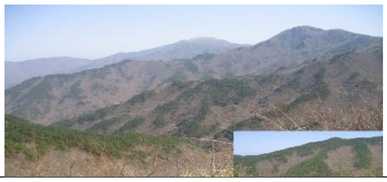 Gneiss Mountain |
| Plateau and Flat | 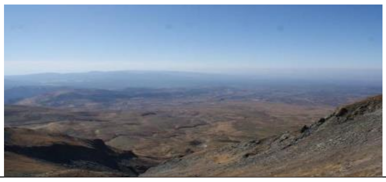 Magma Plateau | 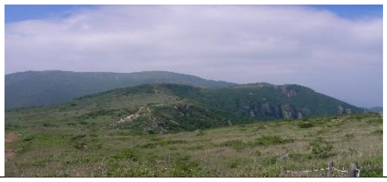 High Flat |
| Basin | 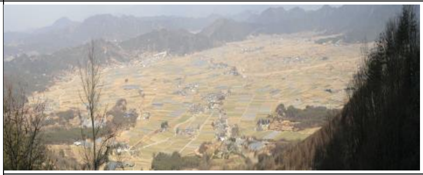 Inland Erosional Basin | 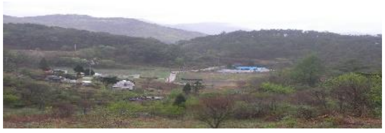 Limestone Basin |
| Piedmont | 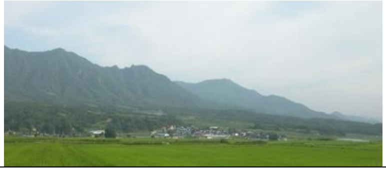 Piedmont | 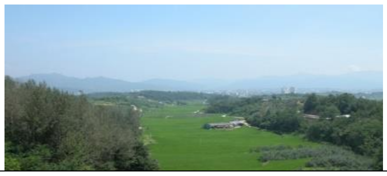 Flat Valley in Piedmont |
| Rock Block and Cliff | 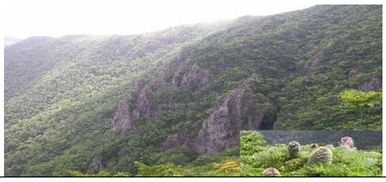 Mountain Cliff | 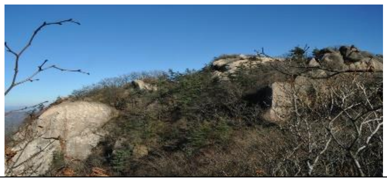 Rock Block |
| Wind hole | 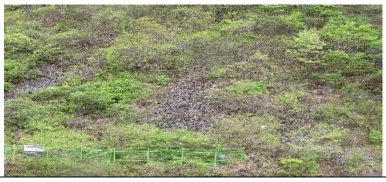 Algific Talus | 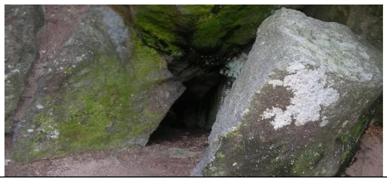 Wind Hole |
| Caldera | 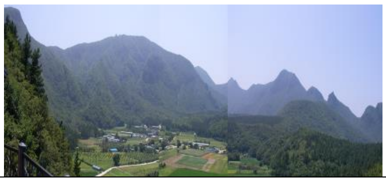 Caldera(Ulneung island) | 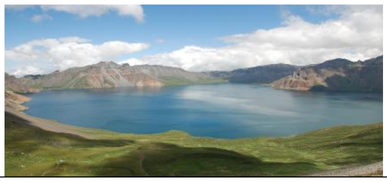 Caldera(Cheonji Mt, Baekdu) |
| | Caldera | |

| | | |
|---|---|---|
| Coastal Landforms and Delta | 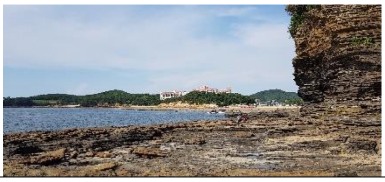 | 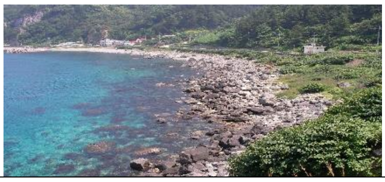 |
| | Rock Beach | Bounder Beach |
| | 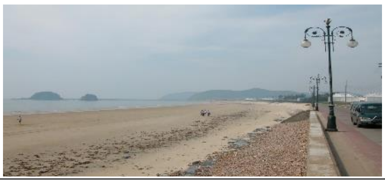 | 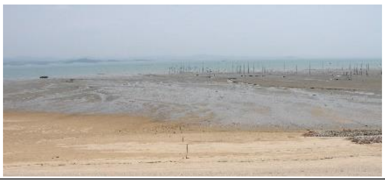 |
| | Sand Beach | Mud and Sand Beach |
| | 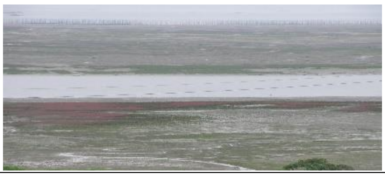 | 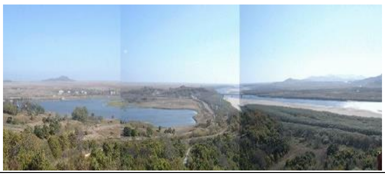 |
| | Mudflat Beach | Delta |
| | 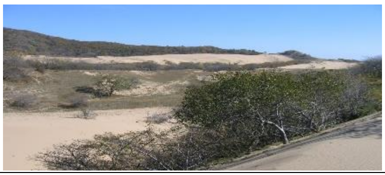 | 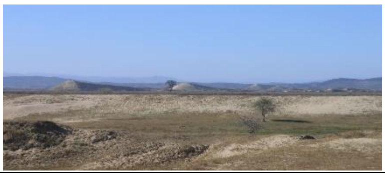 |
| | Sand dune | Sand dune |
| Mountain Bog and Wetland | 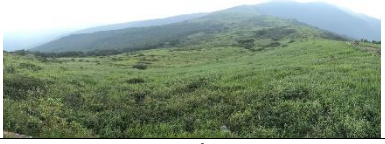 | 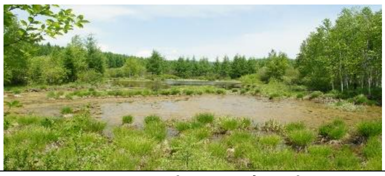 |
| | Mountain Bog | Mountain Wetland |
| Irrigation Pond | 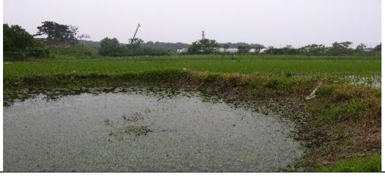 | 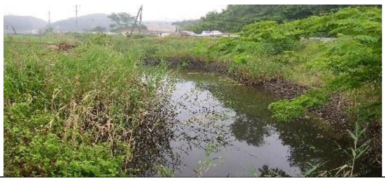 |
| | Small Irrigation Pond | |
| Irrigation Canal | 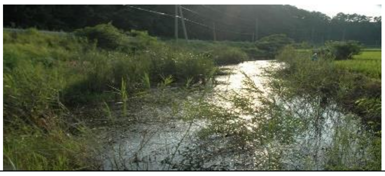 | 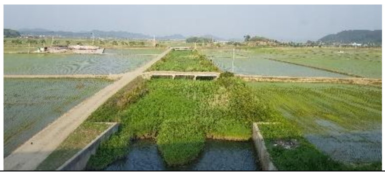 |
| | Irrigation Canal | |
| Fluvial Landforms | 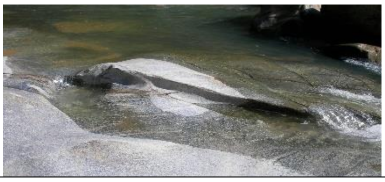 | 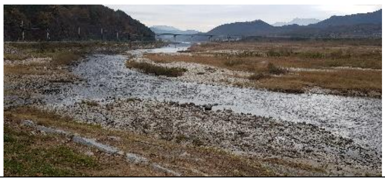 |
| | Rocky Channel | Sand and Gravel Channel |

| | | |
|---|---|---|
| | 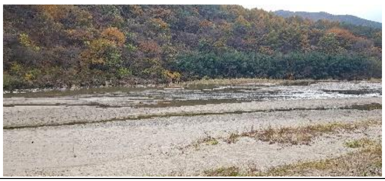 | 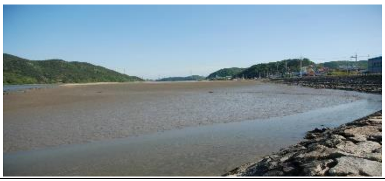 |
| | Sand Channel | Silt and Mud Channel |
| Stream and Paddy Field Wetland | 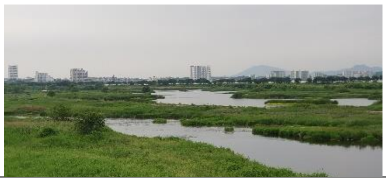 | 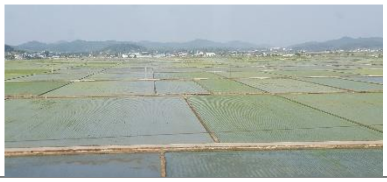 |
| | Inner Wetland in Stream | Paddy Field Wetland |
| Lake Wetland | 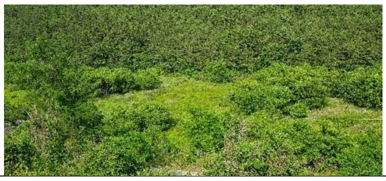 | 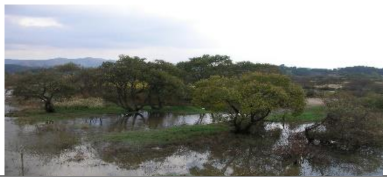 |
| | Tributary Inflow Wetland in Lake | Tributary Inflow Wetland in Lake |
| Baekdudaegan and DMZ | 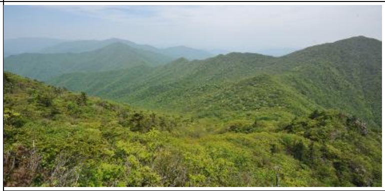 | 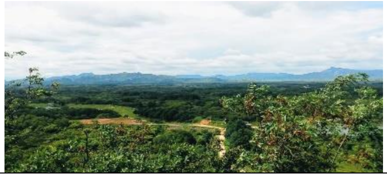 |
| | Baekdudaegan | DMZ Ecozone |
