# Supplementary material for: Hierarchical landform delineation for the habitats of biological communities on the Korean Peninsula
Source: PLoS One. 2021 Nov 5;16(11):e0259651. doi: 10.1371/journal.pone.0259651 (PMC8570509; doi:10.1371/journal.pone.0259651)
Supplement: S2 File — (PDF) [file pone.0259651.s002.pdf]

S2. Landform classification procedures by categories.

Category 1

① Mountain and Plains

Category_1	Classification	
**1:5,000,000**	**Relief**	**Elevation**
Mountains	91 <= Relief < 1,200	elev >= 100
Plains	0 <= Relief < 91	elev < 100
**Map algebra**		
Mountain	*(91 <= Relief < 1,200) and (elev >= 100)*	
Plains	*(0 <= Relief < 91) and (elev < 100)*	

② Fluvial Landform

Category_1	Classification	
1:5,000,000	Threshold number(facc)	Stream Order
Fluvial Landform	Facc >= 1,000,000	1st == Order
**Hydrological modelling and Spatial query**		
Fluvial Landform	*facc >= 1,000,000 → (Select == 1st order)*	

③ Coastal Landform

Category_1	Classification
**1:5,000,000**	**Administration Data**
Coastal Landform	Coastal line == Administration Data
**Spatial query**	
Coastal Landform	*Select == Coastline*

④ Islands Landform

Category_1	Classification
**1:5,000,000**	**Administration Data**
Island	Island Polygon == Administration Data
**Spatial query**	
Islands Landform	*Select == Island*

⑤ Baekdudaegan

Category_1	Classification	
**1:5,000,000**	Threshold number(facc)	**History**
Baekdudaegan	(1st == Order) intersect with Watershed	Sangyeongpyo(a mountain chart))<br>== Daegan

**Hydrological modelling and Spatial query**	
Baekdudaegan	*facc >= 2,000 → (Select == 1st Order) and (intersect with watershed) and (Sangyeongpyo == Daegan)*

⑥ Demilitarized Zone

Category_1	Classification
**1:5,000,000**	**Geopolitics**
DMZ Zone	North and South Truce Line<br>Width of Military Demarcation Line is less 2km

**Headup digitizing: Military Demarcation Line**	
Headup digitizing	*headup digitizing with google earth image*

Category 2

① Mountain and Plains

Category_2	Classification			
**1:1,000,000**	**Relief**	**Elevation/slope**	**Threshold number(facc)**	**Landcover and NDVI**
High Elevation Mt.	250 <= Relief < 1,200	elev >= 1,000	-	-
Middle Elevation Mt.	140 <= Relief < 250	500 <= elev <1,000	-	-
Piedmont	91 <= Relief < 140	100 < elev < 500	-	-
Drainage Divide	-	-	facc >= 100,000 and watershed	-
Water and Lake	-	-	-	Water Body and area >= 100㎢
Wetland in Lake		slope <= 5		Tributary Inflow and – 0.2 < ndvi < 0.3
Alluvial Plains	0 <= Relief < 10	elev <= 30	-	
Undulating Hills	10 <= Relief < 91	30 < elev <= 100	-	

**Map algebra, Hydrological modelling, Spatial query and Headup digitizing**	
High Elevation Mt.	*(250 <= Relief < 1,200) and (Elev >= 1,000)*
Middle Elevation Mt.	*(140 <= Relief < 250) and (500 <= Elev < 1,000)*
Piedmont	*(91 <= Relief < 140) and (100 < Elev < 500)*
Drainage Divide	*facc >= 100,000 → watershed*

Water and Lake	*(landcover == Water Body) and (landcover == lake and area >= 100㎢)*
Wetland in Lake	*(landcover == lake and area >= 100) and (select == endpoint) and (slope <= 5) and (-0.2 < NDVI < 0.3) and headup digitizing*
Alluvial Plains:	*(0 <= Relief < 10) and (elev <= 30)*
Undulating Hills	*(10 <= Relief < 91) and (30 < elev <= 100)*

② Fluvial Landform

Category_2	Classification		
**1:1,000,000**	**Sea direction**	**Earth's Crust**	**Tectonic/Sealevel/geology**
Uplift Coast	East	Continent	Uplift
Rias Coast	West and South	Continent	Sea Level Rise
Volcanic Coast	East and South Sea	Ocean or Continent	Volcanic rock

Map algebra and Spatial query

Uplift Coast	*(direction == East) and (select == Continent) and (select == tectonic)*
Rias Coast	*(direction == west) and (select == Continent) and (select == Sea Level Rise)*
Volcanic Coast	*((direction == east sea) or (direction == south sea)) and ((select == Continent) or (select == Continent)) and (select == Volcanic rock)*

③ Coastal Landform

Category_2	Classification		
**1:1,000,000**	**Sea direction**	**Earth's Crust**	**Tectonic/Sealevel**
Island	West and South	Continent	Sea Level Rise
Volcanic Island	East and South	Ocean or Continent	Volcanic rock

Map algebra and Spatial query

Island	*((direction == west) or (direction == south)) and (select == continent) and (select == Sea Level Rise) and (Select == Island)*
Volcanic Island	*((direction == east) or (direction == south)) and (select == continent) or (elect == Ocean) and (select == Volcanic rock) and (Select == Island)*

Category_2	Classification		
**1:1,000,000**	**Sea direction**	**Earth's Crust**	**Tectonic/Sealevel**
Island	West and South	Continent	Sea Level Rise
Volcanic Island	East and South	Ocean or Continent	Volcanic rock

Map algebra and Spatial query

Island	*((direction == west) or (direction == south)) and (select == continent) and (select == Sea Level Rise) and (Select == Island)*

Volcanic Island	*((direction == east) or (direction == south)) and (select == continent) or (elect == Ocean) and (select == Volcanic rock) and (Select == Island)*

Category 3

① Mountain Landform

Category_3	Classification			
**1: 50,000 ~ 25,000**	**Geology/Tectonic**	**Relief and Elev,Slope,Coast line**	**Threshold number(facc)**	**Landcoer and NDVI**
Mt. Granite	Granite	Condition in Category_2	-	-
Mt.Gneiss Series	Gneiss Series		-	-
Mt.Tertiary Layer	Tertiary Layer		-	-
Mt.Limestone	Limestone		-	-
Mt.Pyroclast	Pyroclast		-	-
Volcanic Mountain	Volcanic Rock	-	-	-
Lava Plateau	Basalt	elev > 800 and Relief <= 300	-	100 < Area < 13,000
High Flat	Except volcanic Rock	Elev > 800 and Relief <= 300	-	5 < Area < 100
Limestone Basin	Limestone	Relief <= 100	-	
Isolated Mountain	-	elev >= 800 including Summit	-	-
Drainage Divide	-	-	facc >= 15.000 and watershed	
Water and Lake	-	-	-	Water Body and 50 <= area <= 100㎢
Wetland in Lake		slope <= 5		Tributary Inflow and -0.2 <NDVI < 0.3
Inland Erosion Basin	Granite with intersect Faultline	Relief <= 100 and Coastline >= 5,000m	-	-
Caldera Basin	Collapsed Crater	Relief <= 10 and elev >= 600	-	-

**Map algebra, Hydrological modelling, Spatial query and Headup digitizing**	
Mt. Granite	*Geology == granite and Mt. in* Category_2
Mt.Gneiss Series	*Geology == Gneiss Series and Mt. in* Category_2
Mt.Tertiary Layer	*Geology == Tertiary Layer and Mt. in* Category_2
Mt.Limestone	*Geology == Limestone and Mt. in* Category_2

Mt.Pyroclast	*Geology == Pyroclast and Mt. in* Category_2
Volcanic Mountain	*Geology == Volcanic Rock and Mt. in* Category_2
Lava Plateau	*Geology == Basalt and (Elev > 800 and Relief <= 300) and (100 < Area < 13,000)*
High Flat	*Geology ne Basalt and (Elev > 800 and Relief <= 300) and (5 < Area < 100)*
Limestone Basin	*Geology == Limestone and Relief <= 100, headup digitizing with Google Image*
Isolated Mountain	*Focalmax(Elev > 800) and elev > 800*
Drainage Divide	*facc >= 15,000 → watershed*
Water and Lake	*(landcover == Water Body) and (landcover == lake and (50 <= area <= 100)*
Wetland in Lake	*(landcover == lake and (50 <= area <= 100) and (select == endpoint) and (slope <= 5) and (-0.2 < NDVI < 0.3), headup digitizing*
Inland Erosion Basin	*Geology == Granite and interslect with faultline and Relief <= 100 and Coastline >= 5,000*
Caldera Basin	*Geology == Volcanic Rock an Relief <= 10 and elev >= 600, headup digitizing with Google Image*

② Plain Landform

Category_3	Classification		
**1: 50,000 ~ 25,000**	**Relief and Elev, slope**	**River mouth Distance form Coastline**	**Landcover and NDVI**
Coastal Plains	Relief <= 91 and elev <= 30	*Coastline < 5,000*	-
Inland Plains	Relief > 91 and 30 <= elev <= 100	*Coastline >= 5,000*	-
Delta Plains	Relief <= 10 and elev <= 5 and Slope < 5	*River mouth and Coastline == 0*	Wetland and Vegetation and Seawater
Water and Lake			Water Body and 10 <= area < 50
Wetland in Lake			Tributary Inflow and -0.2 <NDVI < 0.3
Low Gentle Slope	10 <= relief < 91 and 30 <= elev <= 100	-	-

**Map algebra, Spatial query and Headup digitizing**	
Coastal Plains	*(Relief <= 91 and Elev <= 30) and Coastline < 5,000*
Inland Plains	*(10 < Relief > 91 and 30 < Elev <= 100) and Coastline >= 5,000*
Delta Plains	*(Relief <= 10 and elev <= 5 and Slope < 5) and* Select == 5 or 6st order → intersect == Coastline = 0 → Select == water, wetland, grassland

Water and Lake	*(landcover == Water Body) and (landcover == lake and (10 <= area < 50)*
Wetland in Lake	*(landcover == lake and (10 <= area < 50) and (select == endpoint) and (slope <= 5) and (-0.2 < NDVI < 0.3), headup digitizing*
Low Gentle Slope	*(10 <= relief < 91) and (30 < elev <= 100)*

③ Fluvial Landform

Category_3	Classification			
**1: 50,000 ~ 25,000**	**Relief and Slope, Elev**	**Threshold number(facc)/ Stream Order**	**Geology /Tectonic**	**Category_2/landcover**
Rocky Channel	relief > 91 and Slope > 10	facc >= 15.000 and 1~2st Order	All Bed Rock/Uplift	High Elevation Mt.
Sand and Gravel Channel	10 < relief <= 91 and 10 < elev <= 100 and 5 < Slope <= 10	facc >= 15.000 and 3~4st Order	Granite	Middle Elevation Mt.
Silt and Mud Channel	relief > <= 10 and elev <= 10 and Slope <= 5	facc >= 15.000 and 5~6st Order	All Bed Rock	Alluvial Plains
Water and Lake				Water Body and 10 <= area < 50
Wetland in Lake				Tributary Inflow and –0.2 < NDVI < 0.3

**Map algebra, Hydrological modelling and Spatial query**	
Rocky Channel	*(Relief > 91) and (slope > 10) and (elev > 100) and (facc >= 15,000 → Select == 1~2st Order) and (geology == all Rock and Uplift) and High Elevation Mt.*
Sand and Gravel Channel	*(10 < Relief <= 91) and (5 < slope <= 10) and (10 < elev <= 100) and (facc >= 15,000 → Select == 3-4st Order) and geology == granite and Middle Elevation Mt.*
Silt and Mud Channel	*(Relief <= 10) and (slope <= 5) and (elev <= 10) and (facc >= 15,000 → Select == 5-6st Order) and geology == all BedRock and Alluvial Plains*
Water and Lake	*(landcover == Water Body) and (landcover == lake and (10 <= area < 50)*
Wetland in Lake	*(landcover == lake and (10 <= area < 50) and (select == endpoint) and (slope <= 5) and (-0.2 < NDVI < 0.3), headup digitizing*

④ Coastal Landform

Category_3	Classification				
**1: 50,000 ~ 25,000**	**Sea direction/Headland /Coast type**	**Geology**	**Sea Energy**	**Landcover**	**Tectonic/Sealevel/geology**
Rocky Coast	East/West and Headland	Gneiss, Pyroclast	Wave		Uplift
Sand Coast	East and Bay Beach/ West and headland	Granite	Wave	Sand	Uplift
Sand and Mud Coast	West/Headland Beach	Granite	Wave and Tide		Sealevel rise
Sand and Gravel Coast	East/South and Headland Bay and beach	Granite	Wave		Uplift
Mud Coast	West	Granite, Gneiss	Tide	Tidalflat	Sealevel rise
Mixed Coast	South	Gneiss, Pyroclast	Wave and Tide		Middle

**Map algebra and Spatial query**	
Rocky Coast	*(direction == East or direction == West) and Headland and (Geology == Gneiss or Geology == pyroclast) and Sea engergy == Wave and tectonic == uplift*
Sand Coast	*((direction == East and Coastype == Bay) or (direction == West and Coastype == haedland)) and (Geology == Granite) and Sea engergy == Wave and tectonic == uplift and select = sand*
Sand and Mud Coast	*((direction == West and Coastype == headland beach) and and (Geology == Granite) and (Sea engergy == Wave and Sea engergy == tide) and (select==SeaLevelRise)*
Sand and Gravel Coast	*((direction == East or direction == South) and (Coastype == headland Bay or Coastype == Beach) and (Geology == Granite) and (Sea engergy == Wave) and tectonic == uplift*
Mud Coast	*((direction == West) (Geology == Granite or Gniess)) and (Sea engergy == Tide) and select == mudlfat and (select == Sea Level Rise)*
Mixed Coast	*((direction == south) and (geology == gneiss or pyroclast) and (Sea engergy == Wave and Sea engergy == tide)*

Category 4

① Mountains

Category_4	Classification							
**Under 1:5,000**	**Relief/Elev**	**Slope TPI**	**Geology / tectonic**	**Area (㎢)**	**Threshold number(facc)/ Stream Order**	**Landcover and NDVI**	**Satellite / SWIR**	**Category_1**
Magma Plateau	Relief <= 300 and elev > 800	-	Basalt	40 < Area < 100	-	Grassland	-	-
Highland Flat	Relief <= 300 and Elev > 800	-	Except volcanic Rock	Area < 5	-	Grassland	-	-
Karst Basin	30 < Relief <= 300	-	Limestone	Area < 5	-	-	Google Headup	-
Karst Flat	Relief <= 30	-	Limestone	Area < 15	-	-	Google Headup	-
Wetland Mt.	Relief <= 30 and Magma Plateau and Highland Flat	-		-	-	Wetland and NDVI < 0	-	-
Wind Hole	Field Survey Data							
Piedmont	90 < Relief < 140 and 100 < elev < 500	-	Granite	Area < 5	-	-	-	-
Ridge	-	10 < S TPI < 700		-	-	-	-	-
Valley	-	-626< S TPI < -120		-	-	-	-	-
Rock Block	elev >= 200 and Slope > 7	10 < S TPI < 700	-	-	-	Barren	0 < NDVI < 0.07 and SWIR > 1.57㎛,, Talus, Google Headup	Mountain
Mountain Cliff	Slope > 40	-626< S TPI < -120	Faultline	-	-	-	-	Mountain

Ridge Saddle	-	-120 < S TPI < 10	-	-	-	-	-	-
Crator	Volcanic Crator							
Mountain Bog	Field Survey Data							
Doline Wetland	Relief <= 20	-	Limestone	-	-	Wetland and NDVI < 0	Google Headup	-
Drainage Divide	-	-		-	Facc >= 3,000	-	-	-
Water and Lake	-	-		-		Water Body and 5 < area < 10	-	-
Wetland in Lake						tributary inflow and -0.2 NDVI 0.3		

**Map algebra, Hydrological modelling, Spatial query and Headup digitizing**	
Magma Plateau	*(Relief <= 300 and elev > 800) and Geology == basalt and (40 < Area < 100) and landcover == grassland*
Highland Flat	*Relief <= 300 and elev > 800) and Geology ne basalt and Area < 5) and landcover == grassland*
Karst Basin	*30 < Relief <= 300 and geology == limestone and Area < 5, Headup digitizing with google*
Karst Flat	*Relief <= 30 and geology == limestone and area < 15, Headup digitizing with google*
Wetland Mt.	*Relief <= 30 and Magma Plateau and Highland Flat and landcover == wetland and NDVI < 0*
Wind Hole	*Field Survey data*
Piedmont	*90 < Relief < 140 and 100 < elev < 500 and geology == granite and Area < 5*
Ridge	*10 < S TPI < 700*
Valley	*-626< S TPI < -120*
Rock Block	*(elev >= 200 and Slope > 7) and (10 < S TPI < 700) and landvoer == barren and 0 < NDVI < 0.07 and SWIR > 1.57μm and select == mountians,*<br>*Talus Headup digitizing with google and*
Mountain Cliff	*Slope > 40 and (-626< S TPI < -120) and geology == faultline and select == mountain*
Ridge Saddle	*-120 < S TPI < 10*
Crator	*Geology == Volcanic crator*
Mountain Bog	*Field Survey Data*
Doline Wetland	*Relief <= 20 and Geology == limestone and landcover == Wetland and NDVI < 0*
Drainage Divide	*Facc >= 3,000 → waterbasin*
Water and Lake	*(landcover == Water Body) and (landcover == lake and (5 < area < 10)*
Wetland in Lake	*(landcover == lake and (5 < area < 10) and (select == endpoint) and (slope <= 5) and (-0.2 < NDVI < 0.3), headup digitizing*

② Plains

Category_4	Classification					
**Under 1:5,000**	**Relief/Elev/slope**	**Distance form Coastline**	**Geology**	**Area(㎢)**	**NDVI**	**Landcover**
Bar and Grassland	Relief <= 90 and Elev < 100	-	Granite	-	-	Sand Bar or Grassland
Waterway and Wetland	Relief <= 90 and Elev < 100	-	-	-	NDVI < 0	Canal or Paddy Field Wetland
Small irrigation pond and Canal	Relief <= 90 and Elev < 100	-	-	-	NDVI < 0	Dumbeon or waterway
Small Inland Plains	Relief <= 100 and 30 <= elev <= 100	over 5,000	-	Area<20	-	Paddy Field Wetland
Small Coastal Plains	Relief <= 91 and elev <= 30	under 5,000	-	Area<20	-	Paddy Field Wetland
Inland Wetland	Relief <= 100 and 30 <= elev <= 100	over 5,000	-	-	NDVI < 0	Wetland or Paddy Field Wetland
Isolated Low Hills	10 <= relief < 91 and 30 <= elev <= 100 not ridge	-	-	Area<1	-	Vegetation
Low Relief Gentle Slope	10 <= relief < 91 and 30 <= elev <= 100 and slope < 7	-	Granite	Area<20	-	Dry Field
Paddy Field Wetland	-	-	-	-	NDVI < 0	Paddy Field Wetland
Water and Lake	-	-	-	Area<5	-	Water Body
Wetland in Lake	-	-	-	-	-0.2 < NDVI < 0.3	tributary inflow

**Map algebra, Spatial query and Headup digitizing**	
Bar and Grassland	*(Relief <= 90 and Elev < 100) and geology == granite and (landcover == sandbar or landcover == grassland)*
Waterway and Wetland	*(Relief <= 90 and Elev < 100) and NDVI < 0 and (landcover == canal or landcover == lPaddy Field Wetland)*
Small irrigation pond and Canal	*Relief <= 100 and (1 <= elev <= 100) and NDVI < 0 and (landcover == Dumbeong or landcover == waterway)*

Small Inland Plains	*Relief <= 100 and (30 <= elev <= 100) and coastline > 5,000 and area < 20 and landcover == Paddy Field Wetland*
Small Coastal Plains	*Relief <= 91 and elev <= 30 and coastline < 5,000 and Area < 20 and landcover == Paddy Field Wetland*
Inland Wetland	*Relief <= 100 and (30 <= elev <= 100) and coastline > 5,000 and (landcover == Paddy Field Wetland or landcover == Wetland)*
Isolated Low Hills	*((10 <= relief < 91) and (30 <= elev <= 100) ne ridge) and area < 1 and landcover == vegetatioon*
Low Relief Gentle Slope	*(10 < Relief < 91) and (30 < elev < 100) and slope < 7 and geology == granite and area < 5 and landcover == Dry Field*
Paddy Field Wetland	*landcover == Paddy Field Wetland*
Water and Lake	*Area < 5 and landcover == Water Body and area < 5*
Wetland in Lake	*(landcover == lake and (area < 5) and (select == endpoint) and (slope <= 5) and (-0.2 < NDVI < 0.3), headup digitizing*

③ Fluvial Landform

Category_4	Classification						
**Under 1:5,000**	**Relief/Elev/slope**	**Slope TPI**	**Stream levee Distance/ geology**	**Area /count**	**Threshold number(facc)/ Stream Order**	**Landcover**	**Geololgy / tectonic**
River Terrace	160 <= Relief <= 207 and Elev < 100	0 < S_TPI < 40	out_SDT < 250	-	-	Sand Bar and Grassland	-
Alluvial Island	-	-	Granite	Area>0.1	-	Bar within Steam Channel	-
Riparian Wetland	-	S_TPI < 0	in_SDT < 100	Area>2	-	wetland within Steam Channel	-
Riverside Wetland	Relief <= 10	S_TPI < 0	out_SDT < 100	Area<100	-	External Wetland and Paddy Field Wetland	-
Braided Stream Channel	Relief <= 5	-	Stream channel	Count>10	-	Bar in within Steam Channel	-
Bar	-	-	Stream channel	Count<10		Bar within Steam Channel	-
Riverside Land	Relief <= 5	-	Stream channel	-	-	river levee and Stream channel	-
Stream and Lake	-	-	out_SDT < 200	-	-	water body	-

Wetland in Lake	-	-	-	area<2	tributary Inflow	-	-
Fluvial Cliff	Slope > 40	-626< S TPI < -120	-	-	-	Google headup	Terrace Cliff/Faultline and Incised Stream

**Map algebra, Hydrological modelling and Spatial query**	
River Terrace	*(160 <= Relief <= 207) and Elev < 100 and (160 <= Relief <= 207) and out_SDT < 250 and (landcover == Sand bar or landcover == grassland)*
Alluvial Island	*Geology == granite and Area > 0.1 and (Landcover == Bar within Steam Channel)*
Riparian Wetland	*S_TPI < 0 and in_SDT < 100 and Area > 2 and (landcover == water body or and landcover == barren or landcover == grassland*
Riverside Wetland	*Relief <= 10 and S_TPI < 0 and out_SDT < 100 and Area < 100 and(landvoer == wetland or landcover == paddy field wetland)*
Braided Stream Channel	*Relief <= 5 and select == stream channel and Count > 10 and landcover == bar*
Bar	*select == stream channel and Count < 10 and landcover == bar*
Riverside Land	*Relief <= 5 and select == stream channel and landcover == barren*
Stream and Lake	*out_SDT < 500 and landcover == water body*
Wetland in Lake	*landcover == lake and (area < 5) and (select == endpoint) and (slope <= 5) and (-0.2 < NDVI < 0.3), headup digitizing*
Fluvial Cliff	*Slope > 40 and (-626< S TPI < -120) and geology == faultline and select == incised Stream*

④ Coastal Landform

Category_4	Classification				
**Under 1:5,000**	**Relief/Elev**	**Geology /Tectonic**	**Wave /Tide/wind**	**Coastal Line Distance / landform**	**Landcover/area㎢**
Coastal Terrace	7 <= Relief <= 70 and 10 <= Elev <= 90	Pyroclast and Sedimentary Rock/Uplift	Wave	CLD < 250	area < 2
Rocky Beach	-	Gneiss, Pyroclast/Uplift	Wave	Headland	area < 2
Sand Beach	-	Granite	Wave	East and West/Beach and Headland	Sand beach, area < 2
Sand and Mud Beach	-	Granite	Wave/Tide	West Bay/Beach	Sand Beach, Tidalflat, area < 2
Sand and Gravel Beach	-	Granite	Wave	Headland/ East and South Beach	Sand Beach, area < 2
Tidalflat	-	-	Tide	West	Tidalflat, area < 2
Mixed Coast	-	-	Wave/Tide	South Beach	Tidalflat, area < 2
Sandune Wetland	-	-	Wave	East and West	Saltmarsh, area < 2
Sand Dune	-	-	Wave/wind	East and West/Beach	Sanddune, area < 2

Salt Marsh	-	-	Tide	West Beach	Saltmarsh, area < 2
Lagoon	-	-	Wave	East	Lagoon, area < 2
Coastal Cliff	-	-	Wave	East/West and Headland	Google headup
Headland	-	gneiss, pyroclast,uplift	Wave	East/West convex	area < 2

**Map algebra and Spatial query**	
Coastal Terrace	*7 <= Relief <= 70 and 10 <= Elev <= 90 and (geology == pyroclast or geology == sedimentary rock) and tectonic == uplift and sea energy == wave and CLD < 250 and area < 2*
Rocky Beach	*(geology == Gneiss or geology == Pyroclast) and tectonic == Uplift and sea energy == wave and coastal landform == headland and area < 2*
Sand Beach	*geology == Granite and ernergy == wave and (direction == east or (direction == west and coastype == beach and coastal landform == headland beach)) and landcover == sand and area < 2*
Sand and Mud Beach	*((direction == East or direction == South) and (Coastype == headland Bay or Coastype == Beach) and (Geology == Granite) and (Sea engerge == Wave) and tectonic == uplift and area < 2*
Sand and Gravel Beach	*((direction == East or direction == South) and (Coastype == headland Bay or Coastype == Beach) and (Geology == Granite) and (Sea engerge == Wave) and tectonic == uplift and area < 2*
Tidalflat	*((direction == West) (Geology == Granite or Gniess)) and (Sea engerge == Tide) and select == mudlfat and (select==SeaLevelRise)and area < 2*
Mixed Coast	*((direction == south) and (geology == gneiss or pyroclast) and (Sea engerge == Wave and Sea egerge == tide) and area < 2*
Sandune Wetland	*sea energy == wave and (direction == east or direction == west) and landcover == saltmarsh and area < 2*
Sand Dune	*(sea energy == wave and strong wind) and (direction == east or direction == west) and landcover == sanddune and area < 2*
Salt Marsh	*direction == west and sea energy == tide and landcover == saltmarsh and area < 2*
Lagoon	*sea energy == wave and direction == east and landcover == Lagoon and area < 2*
Coastal Cliff	*sea energy == wave and ((direction == east or west) and headland), headup digitizing with google*
Headland	*tectonic == uplift and sea energy == wave and coastline == east or west convex and geology ==gneiss or geology == pyroclast) and area < 2*

⑤ Islands

Category_4	Classification				
**Under 1:5,000**	**Relief/Elev**	**Coast shape/area**	**Geology /Tectonic/slope**	**Wave and Tide**	**Landcover**
Mud Beach		Small Bay, area < 2	Granite Pyroclast	Tide	TidalFlat
Salt Marsh		Backward Sanddune, area < 1	Granite Pyroclast	Wave and Tide	Saltmarsh
Sand Beach		Small bay, area < 1	Granite Pyroclast	Wave	Sand beach

Sanddune		Sand beach, area < 1	Granite Pyroclast	Wave	SandDune
Lagoon		Backward Sanddune, area < 1	Granite Pyroclast	Wave	Lagoon
Tidal Flat		Small Bay, area < 1	Granite Pyroclast	Tide	Tidalflat
Cliff		Headland, area < 1	Faultline/slope	Wave	-
Coastal Terrace	0 <= Relief <= 70 and 1 <= Elev <= 50	area < 1			

Map algebra and Spatial query

Mud Beach	*coastline == concave and area < 2 and (geology == granite or geology == gneiss or geology == pyroclast) and sea energy = tide and landcover == tidalflat*
Salt Marsh	*area < 1 and (geology == granite or geology == gneiss or geology == pyroclast) and (sea energy = tide or sea energy == wave) and landcover == saltmarsh*
Sand Beach	*coastline == concave and area < 1 and (geology == granite or geology == pyroclast) and sea energy = wave and landcover == sand beach*
Sanddune	*coastline == concave and area < 1 and geology == granite and sea energy == wave and landcover == sanddune*
Lagoon	*sea energy == wave and area < 1 and (geology == granite or geology == pyroclast) and sea energy == wave and landcover = water and lagoon*
Tidal Flat	*coastline == concave and area < 1 and ea energy == tide and landcover == tidalflat*
Cliff	*coastline == convex and geology == faultline and slope > 40 and sea energy == wave*
Coastal Terrace	*0 <= Relief <= 70 and 5 <= Elev <= 50 and (geology == pyroclast or geology == sedimentary rock) and tectonic == uplift and sea energy == wave and area < 2*

⑥ Baekdudaegan Ecozone

Category_4	Classification	
**Under 1:5,000**	**Geology**	**Elevation**
Baekdudaegan Ecozone	7 Geological Zone base on 6 Bedrock(Granite, Gneiss series, Limestone, Basalt)	elev >= 350

Map algebra and Spatial query

Baekdudaegan 1,2,3 Ecozone	*geology == granite and elev >= 350 → 1,2,3 ecozone*
Baekdudaegan 4,5 Ecozone	*geology == limestone and elev >= 350 → 4,5 ecozone*
Baekdudaegan 6 Ecozone	*geology == gneiss series and elev >= 350 → 6 ecozone*
Baekdudaegan 7 Ecozone	*geology == basalt and elev >= 350 → 7 ecozone*

⑦ DMZ Ecozone

Category_4	Classification
**Under 1:5,000**	**Flow Accumulation**
DMZ Ecozone	Facc > 2,000 and Basins
**Map algebra and Spatial query**	
DMZ Ecozone 1	Facc > 2,000 → flow accumulation == watershed 1
DMZ Ecozone 2	Facc > 2,000 → flow accumulation == watershed 2
DMZ Ecozone 3	Facc > 2,000 → flow accumulation == watershed 3
DMZ Ecozone 4	Facc > 2,000 → flow accumulation == watershed 4
DMZ Ecozone 5	Facc > 2,000 → flow accumulation == watershed 5
DMZ Ecozone 6	Facc > 2,000 → flow accumulation == watershed 6
DMZ Ecozone 7	Facc > 2,000 → flow accumulation == watershed 7

⑧ Biodiversity and Geodiversity, Weathering Tendency in Korea Peninsular

<table>
<tr><th>Category</th><th colspan="2">Criteria</th></tr>
<tr><td rowspan="2">Biodiversity and Geodiversity</td><td>Data</td><td>Biodiversity: 160 Thousands Biota in Korea Peninsular → Shannon's index
Geodiversity: Elevation, Slope, Relief, Curvature, TPI, Landform classifications → Shannon's index → Geodiversity</td></tr>
<tr><td>Correlation</td><td>Total Cor. 0.39 with Geodiversity
Over 0.039 of Biodiversty Cor. 0.93 with Geodiversity</td></tr>
<tr><td rowspan="6">Weathering</td><td>Data</td><td>Climate: Bioclim data, Warm's index, Coldindex, Continentality,
Elevation: DEM, TWI, Solar radiation
Geology: Weathering Difference each Bedrock</td></tr>
<tr><td rowspan="5">Weathering Tendency</td><td>Shallow mechanical Weathering
([temper] <= 9) and ([precipt00] <= 1000) and ([dem] >= 600)</td></tr>
<tr><td>Deep mechanical deep Weathering
([temper] <= 9) and ([Precipt00] >= 1000) and ([Precipt00] <= 2200) and ([dem] >= 600) and (([Geol] = 2) or ([Geol] = 4) or ([Geol] = 11) or ([Geol] = 12) or ([Geol] = 17) or ([Geol] = 28) or ([Geol] = 27) or ([Geol] = 32))</td></tr>
<tr><td>Mechanical W > Chemical Weathering
([temper] <= 9) and ([precipt00] <= 1000) and ([dem] >= 600)</td></tr>
<tr><td>Chemical W > Mechanical Weathering
(([temper] >= 11) and ([temper] <= 32)) and (([precipt00] >= 1110) and ([precipt00] <= 2200))</td></tr>
<tr><td>Deep Chemical Weathering
((([temper] >= 9) and ([temper] <= 32)) and (([precipt00] >= 1110) and ([precipt00] <= 2200)) and (([Geol] = 2) or ([Geol] = 4) or ([Geol] = 11) or ([Geol] = 12) or ([Geol] = 17) or ([Geol] = 28) or ([Geol] = 27) or ([Geol] = 32))) and ([Wi100] >= 87)</td></tr>
</table>
