# Supplementary material for: Hierarchical landform delineation for the habitats of biological communities on the Korean Peninsula
Source: PLoS One. 2021 Nov 5;16(11):e0259651. doi: 10.1371/journal.pone.0259651 (PMC8570509; doi:10.1371/journal.pone.0259651)

S3. Landform classification result maps.

Category 1

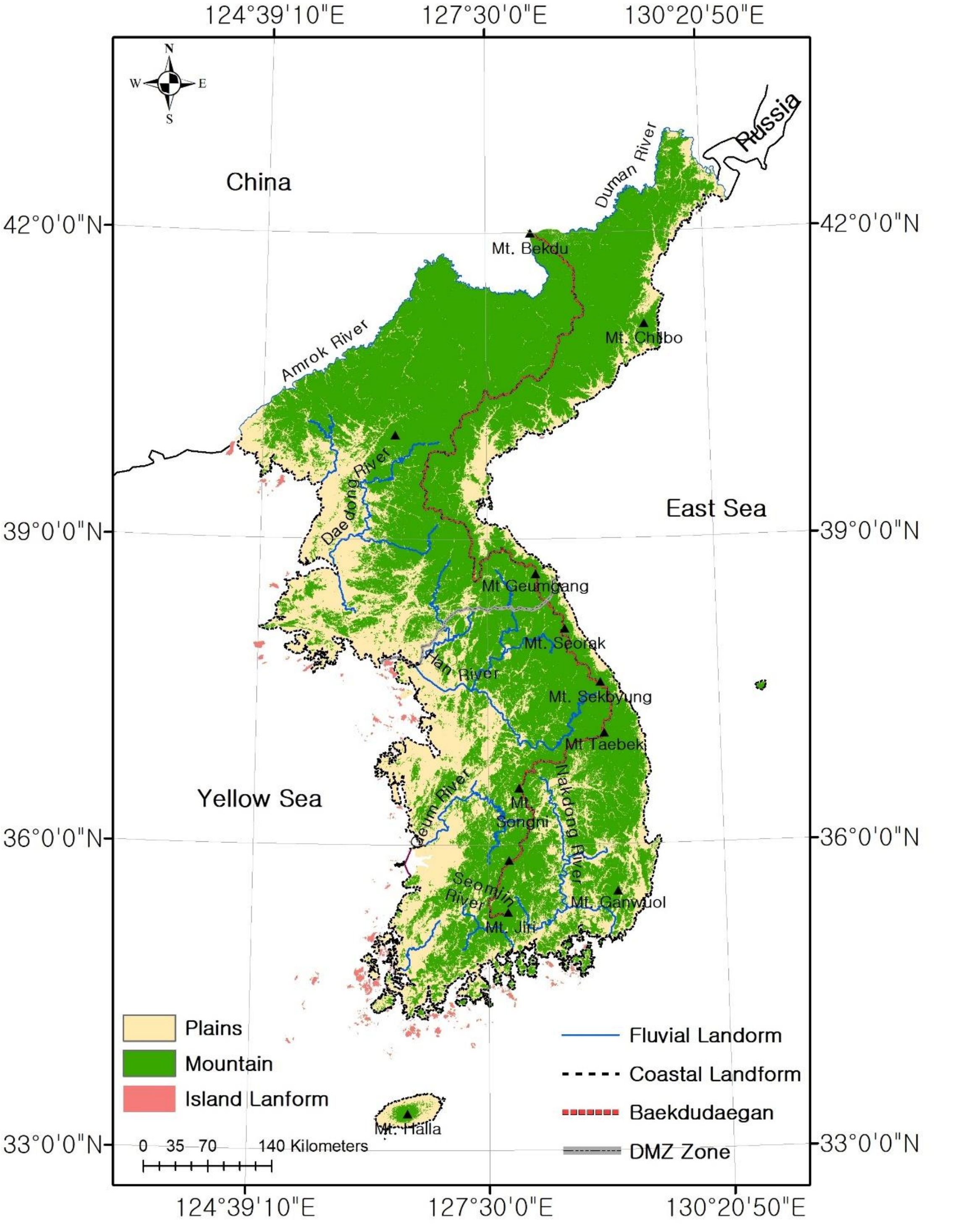

Category 2

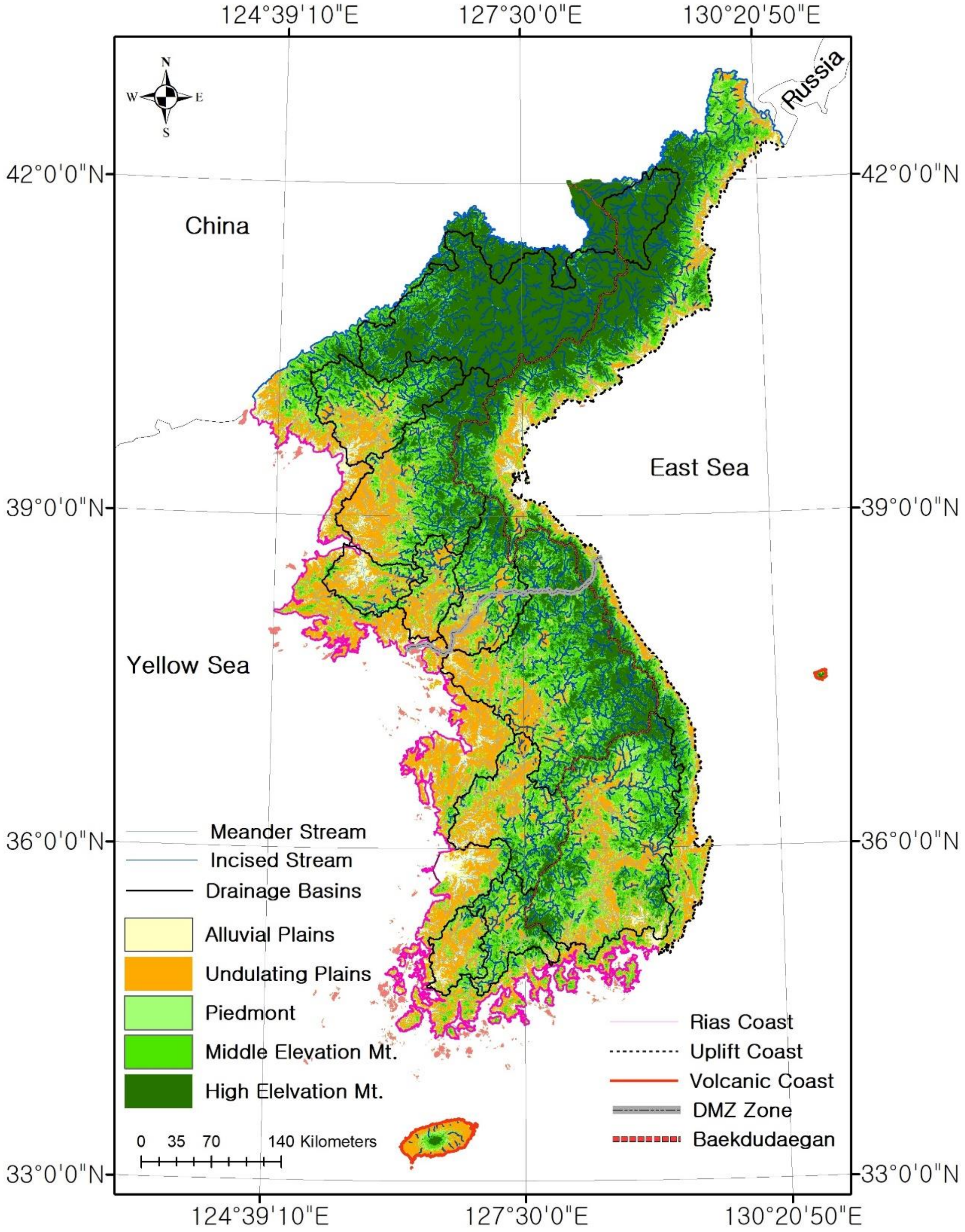

124°39'10"E
127°30'0"E
130°20'50"E
42°0'0"N
39°0'0"N
36°0'0"N
33°0'0"N
N
S
W
E
Russia
China
East Sea
Yellow Sea
Meander Stream
Incised Stream
Drainage Basins
Alluvial Plains
Undulating Plains
Piedmont
Middle Elevation Mt.
High Elelvation Mt.
Rias Coast
Uplift Coast
Volcanic Coast
DMZ Zone
Baekdudaegan
0 35 70 140 Kilometers

Elevation

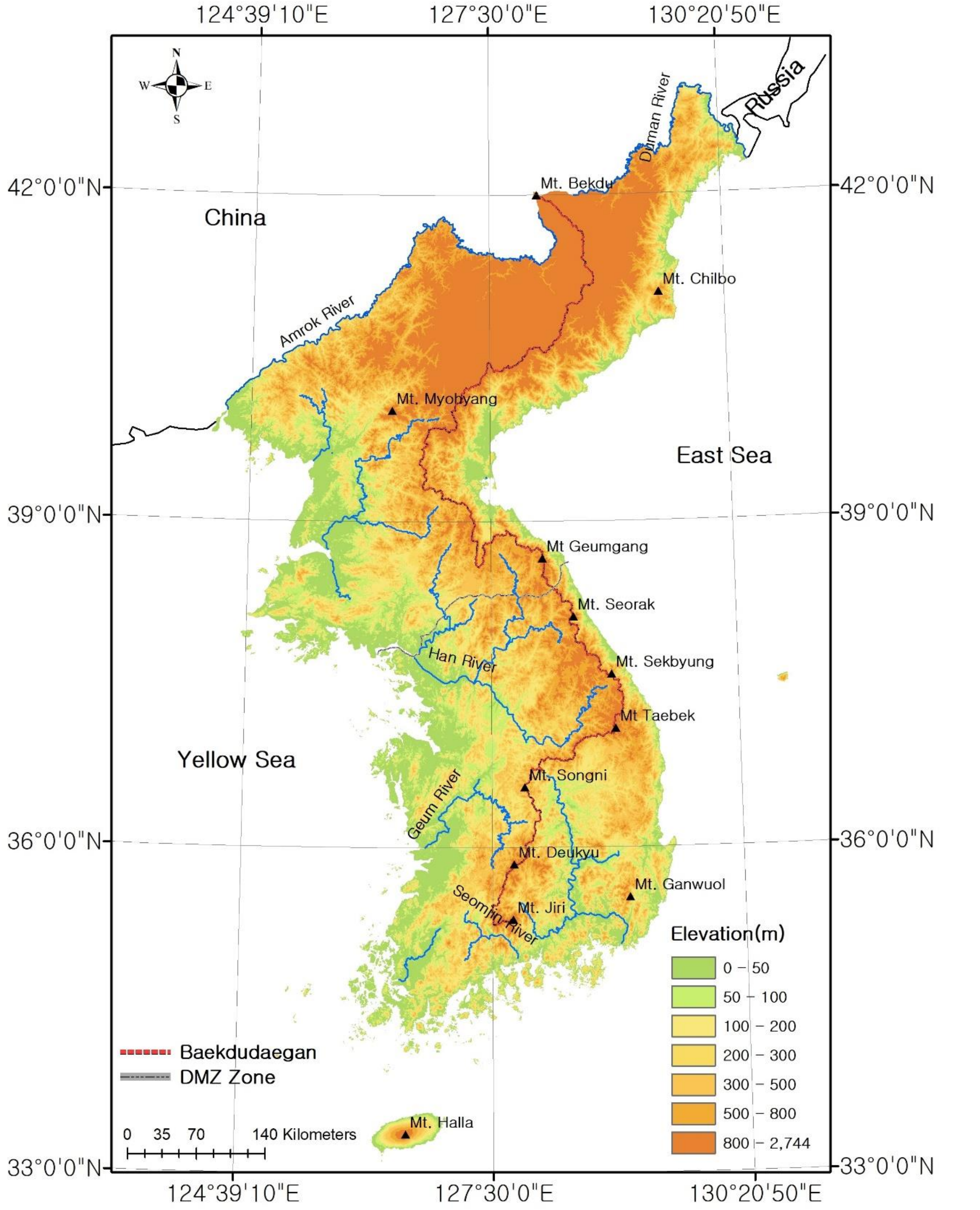

124°39'10"E
127°30'0"E
130°20'50"E
42°0'0"N
39°0'0"N
36°0'0"N
33°0'0"N
N
S
W
E
Russia
Duman River
China
Mt. Bekdu
Mt. Chilbo
Amrok River
Mt. Myohyang
East Sea
Mt Geumgang
Mt. Seorak
Han River
Mt. Sekbyung
Mt Taebek
Yellow Sea
Mt. Songni
Geum River
Mt. Deukyu
Mt. Ganwuol
Seomjin River
Mt. Jiri
Elevation(m)
0 – 50
50 – 100
100 – 200
200 – 300
300 – 500
500 – 800
800 – 2,744
Baekdudaegan
DMZ Zone
Mt. Halla
0 35 70 140 Kilometers

Weathering

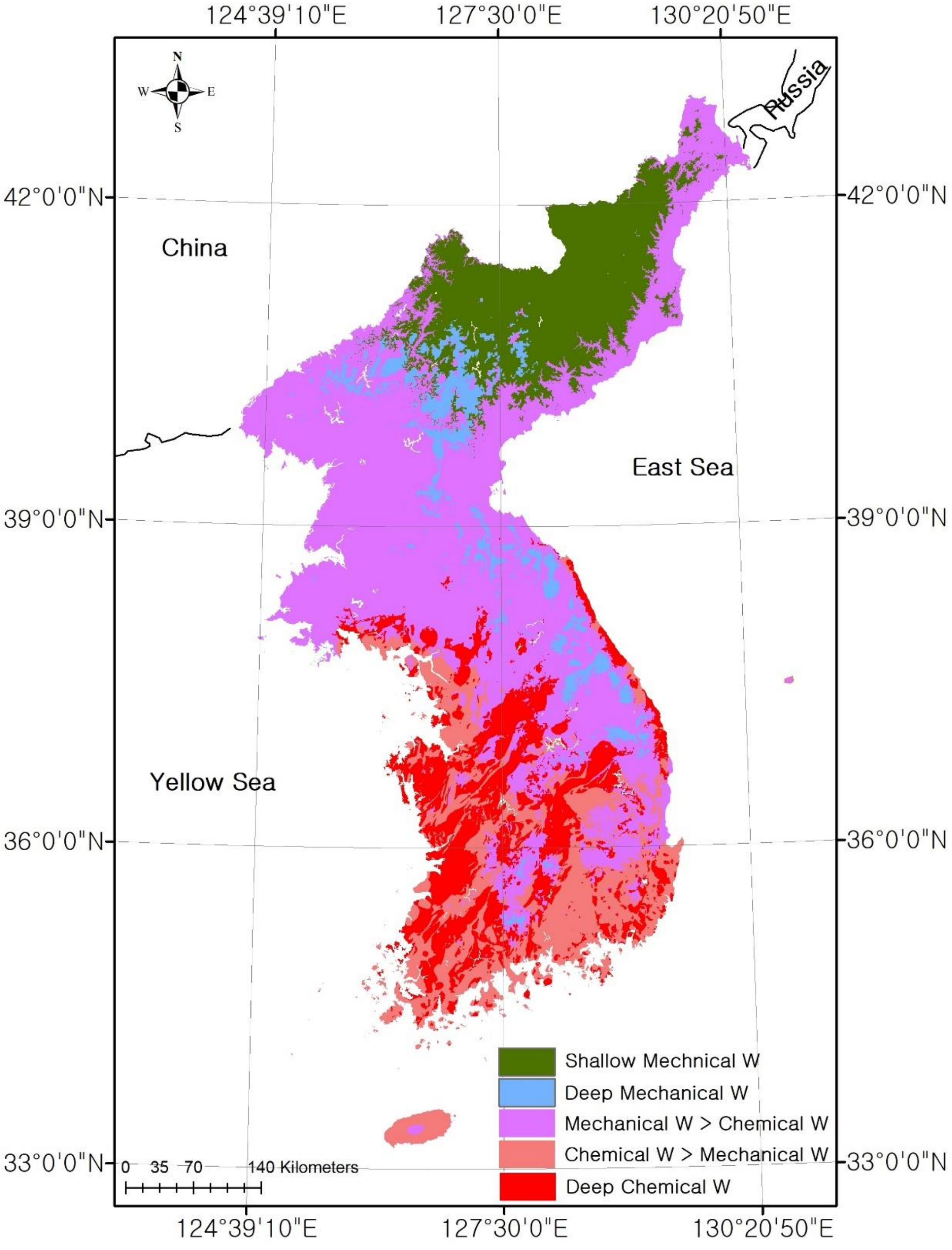
124°39'10"E
127°30'0"E
130°20'50"E
42°0'0"N
39°0'0"N
36°0'0"N
33°0'0"N
N
W
E
S
China
Russia
East Sea
Yellow Sea
0 35 70 140 Kilometers
Shallow Mechnical W
Deep Mechanical W
Mechanical W > Chemical W
Chemical W > Mechanical W
Deep Chemical W

Biodiversity

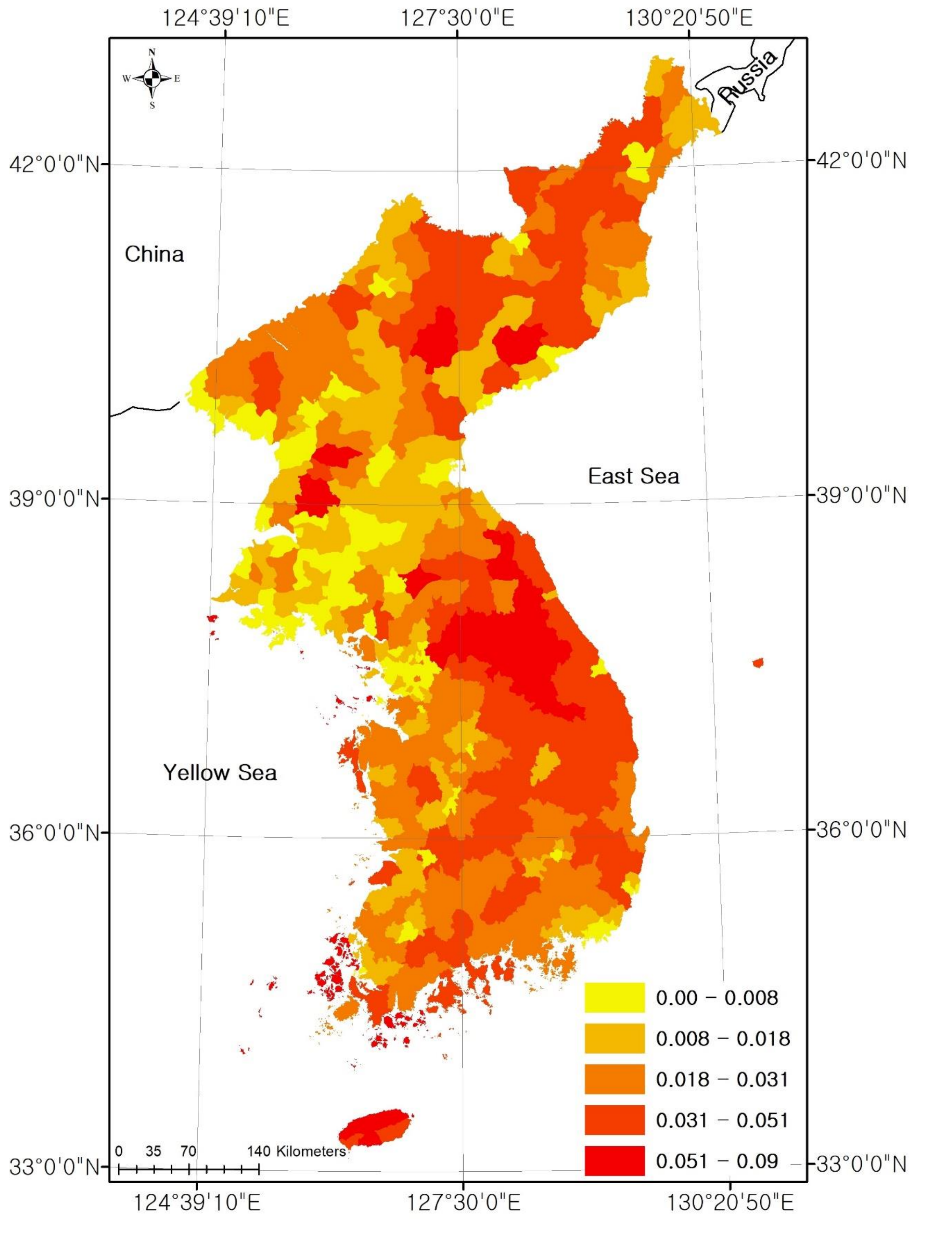

Geodiversity

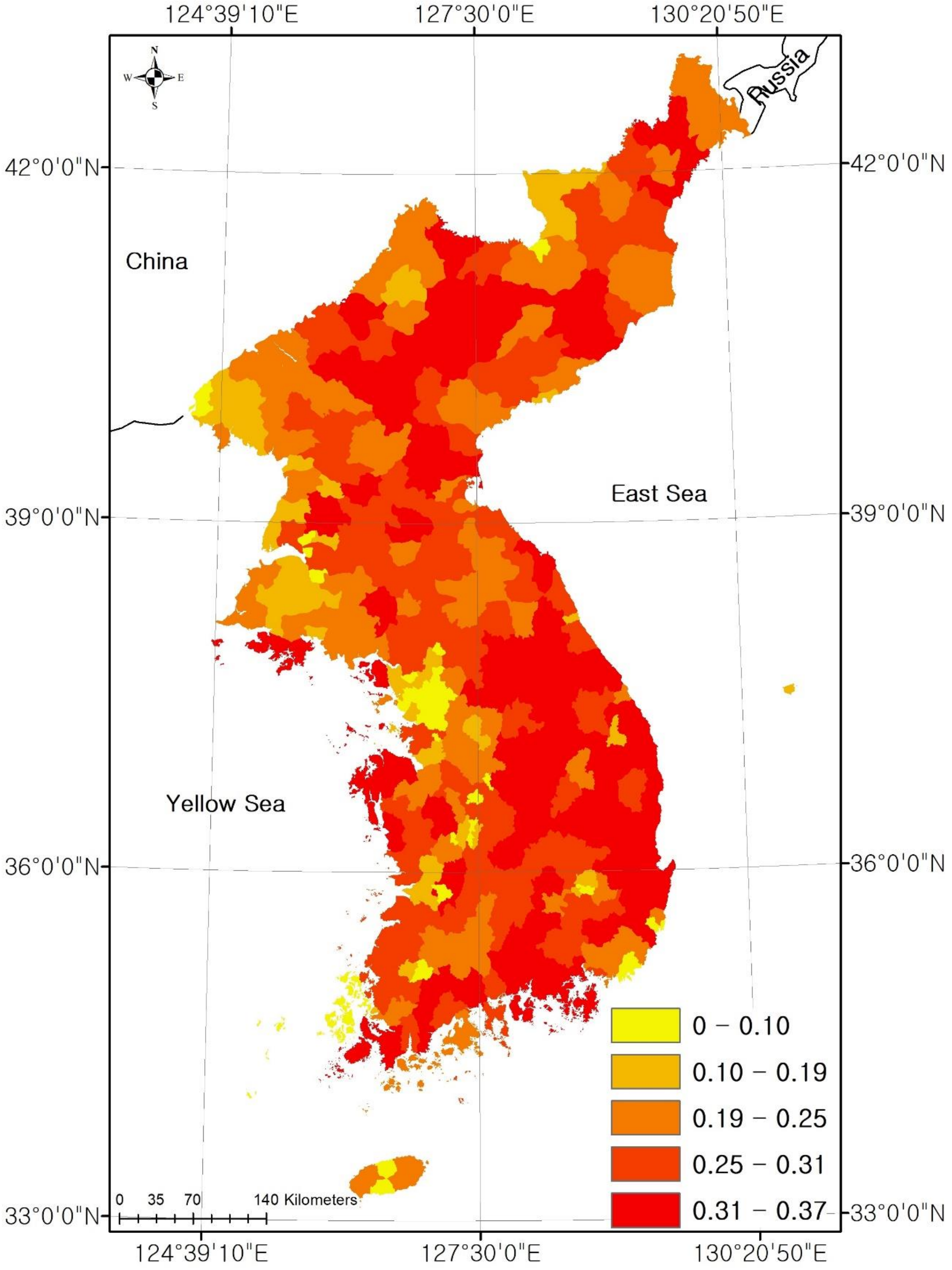

Index area for each categories

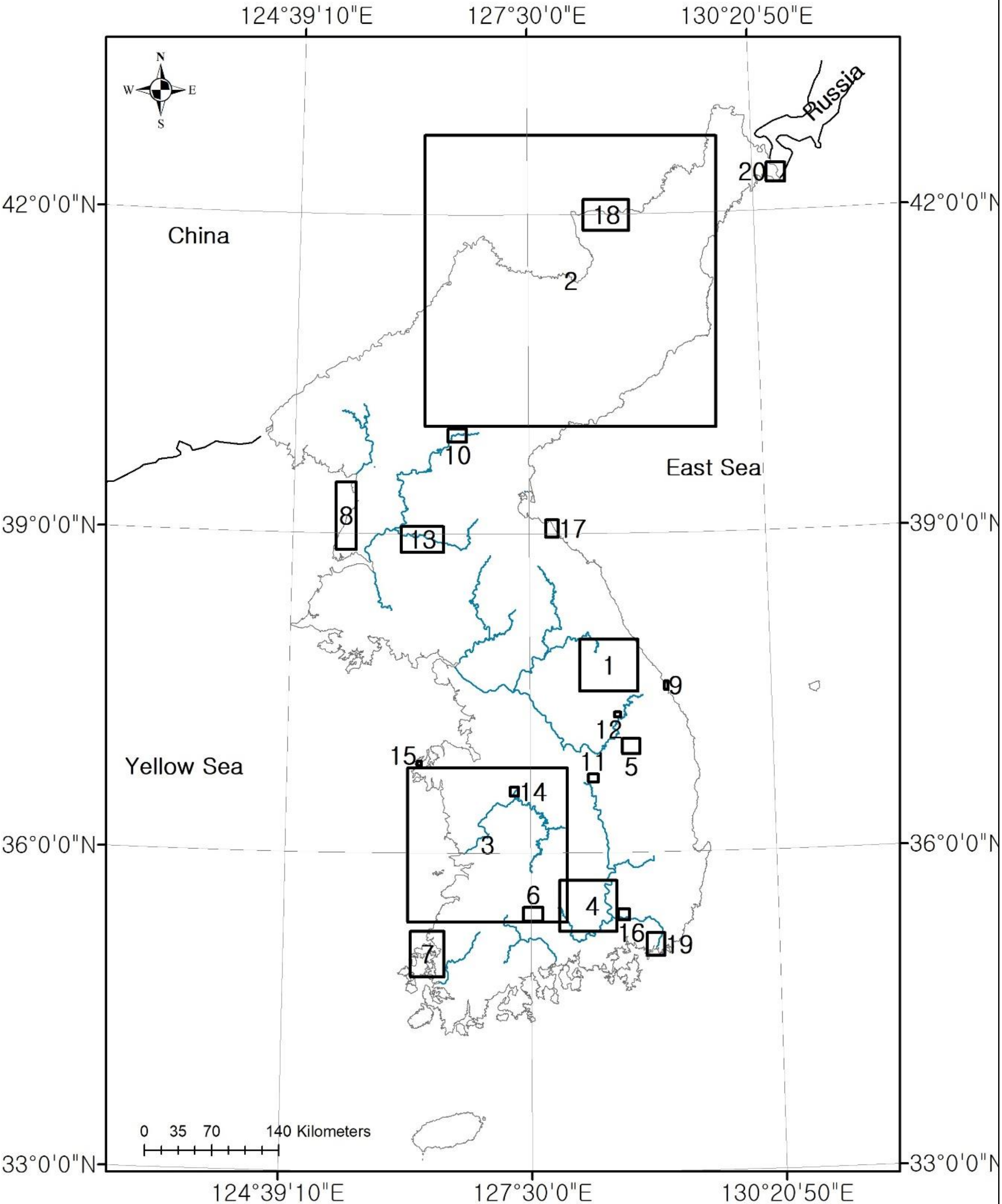

1. Category 3: Mt. Seolak

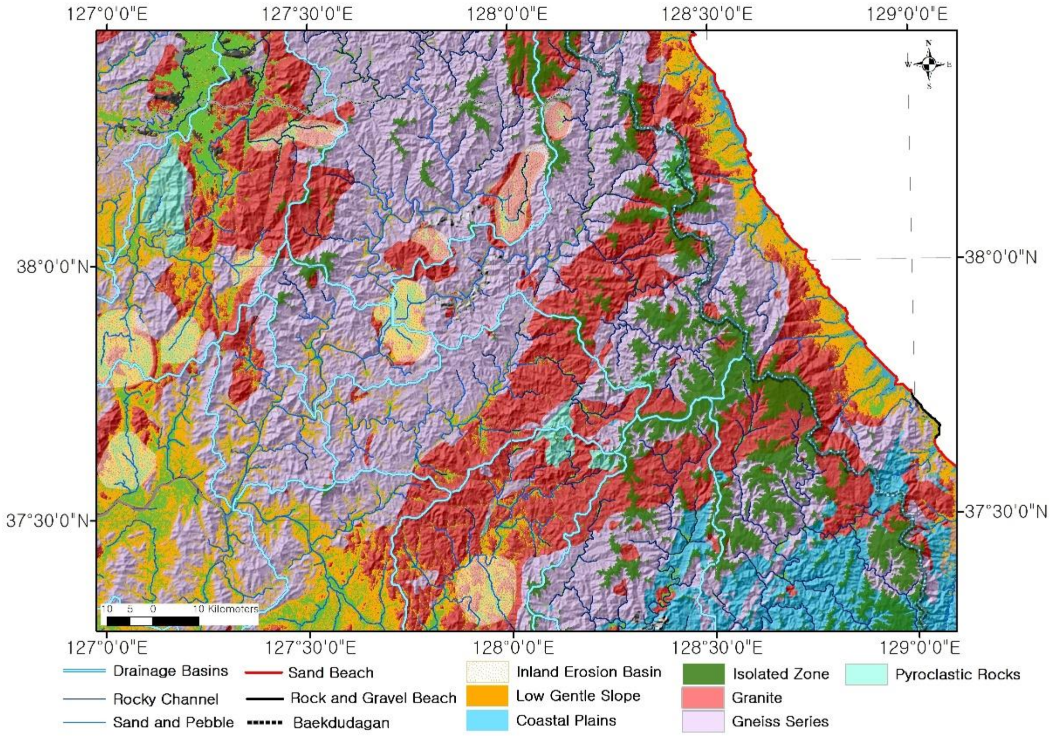

2. Category 3: Gaema Platuae and Mt. Baekdu

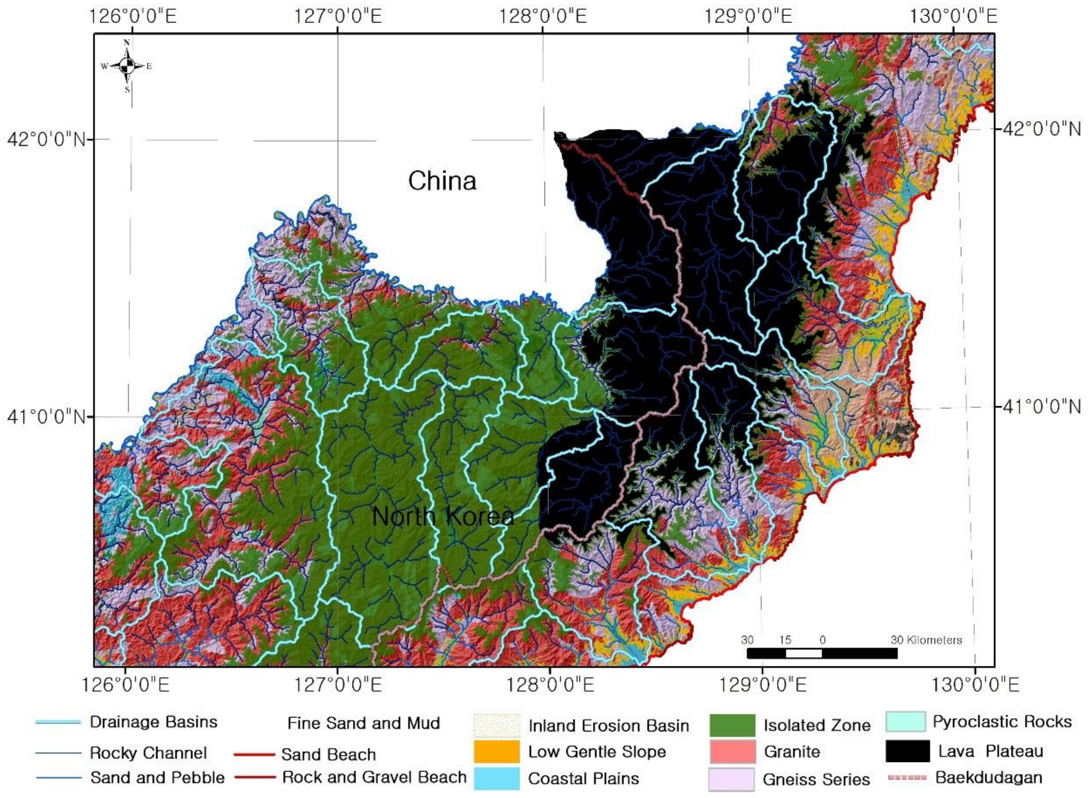

3. Category 3: Coastal plains, Hills, Basin

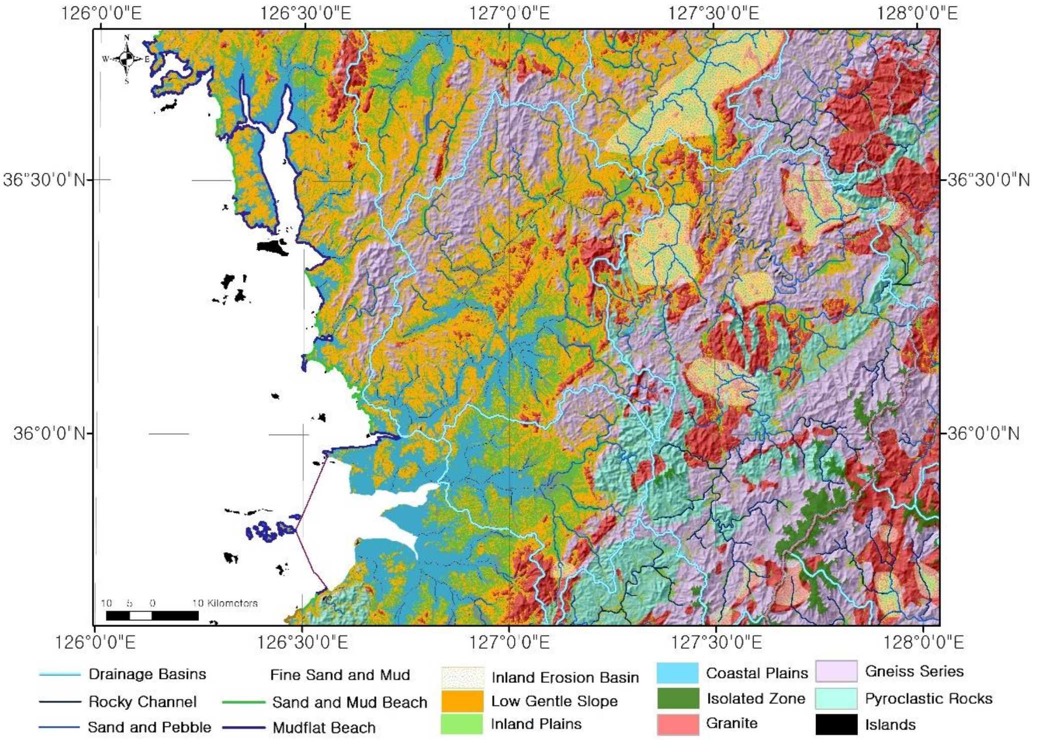

4. Category 3: Middle of Nakdong River

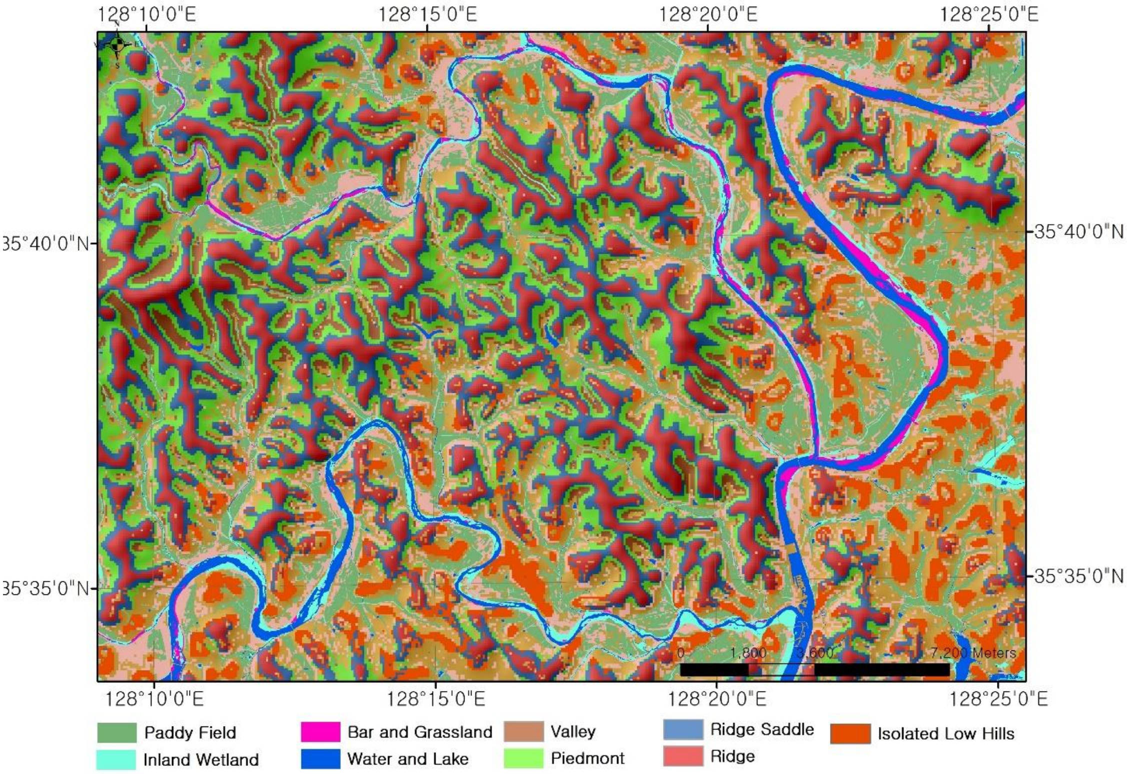

5. Category 3: Mt. Sobaek

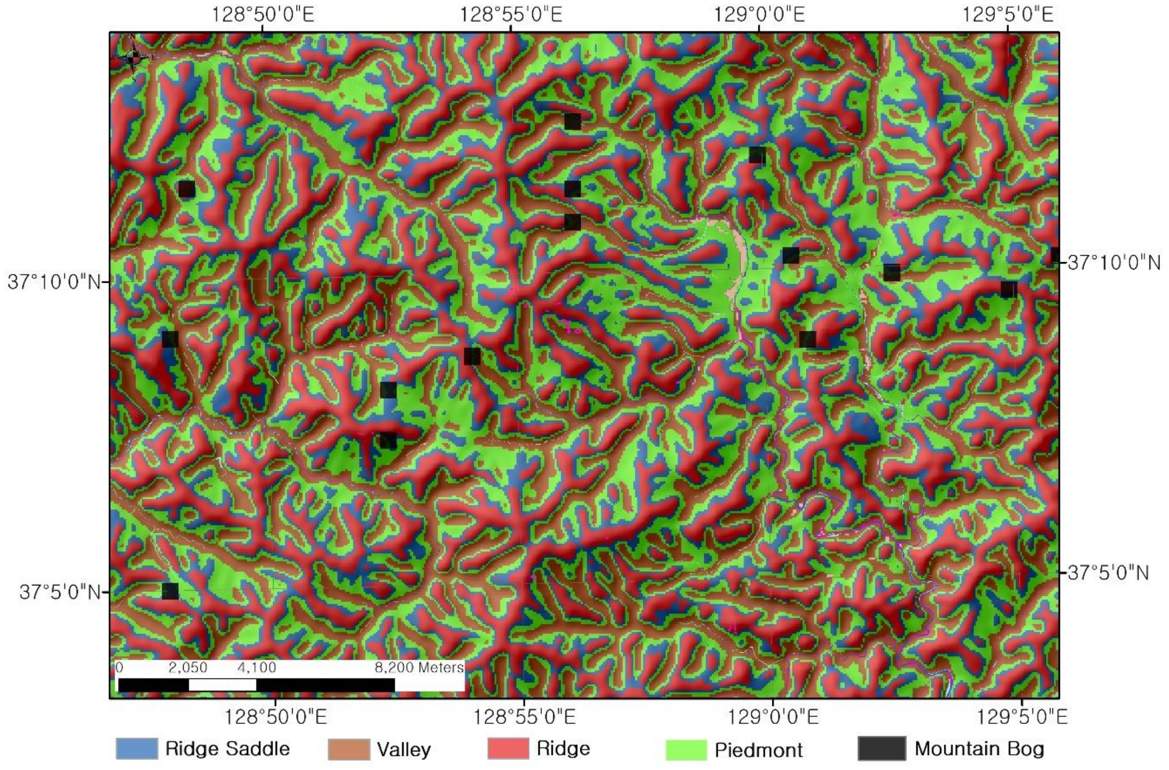

6. Category 3: Basin Unbong

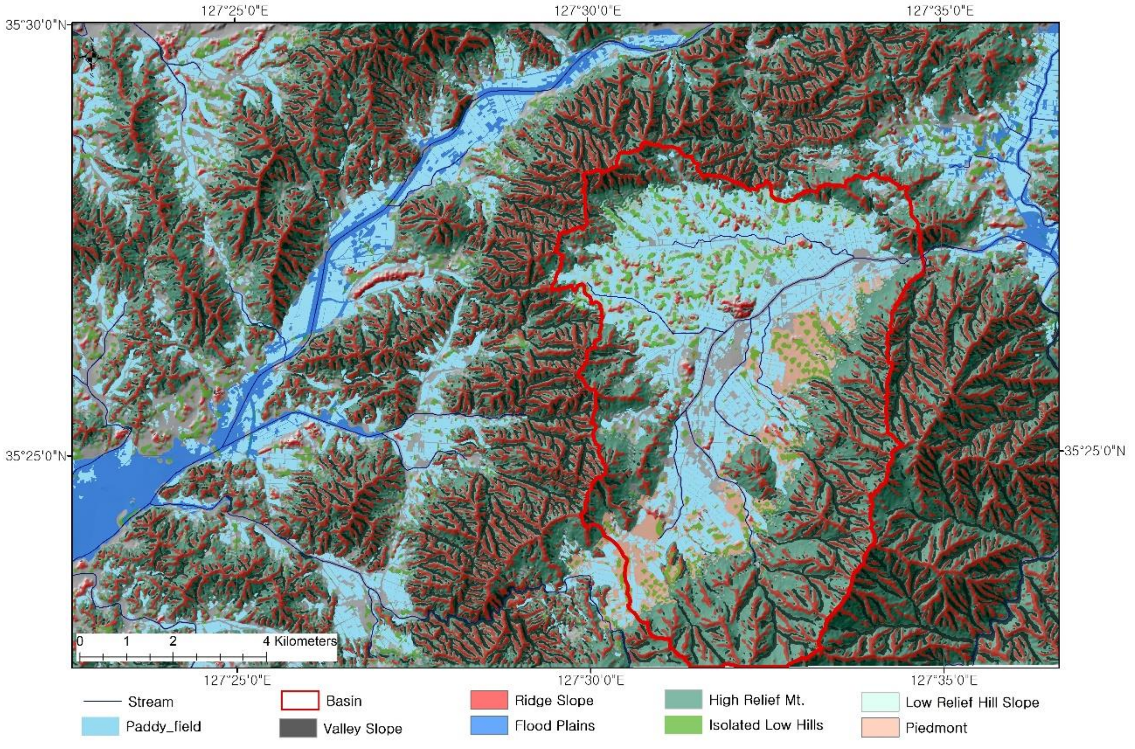

7. Category 3: Tidal flat, Hampyung

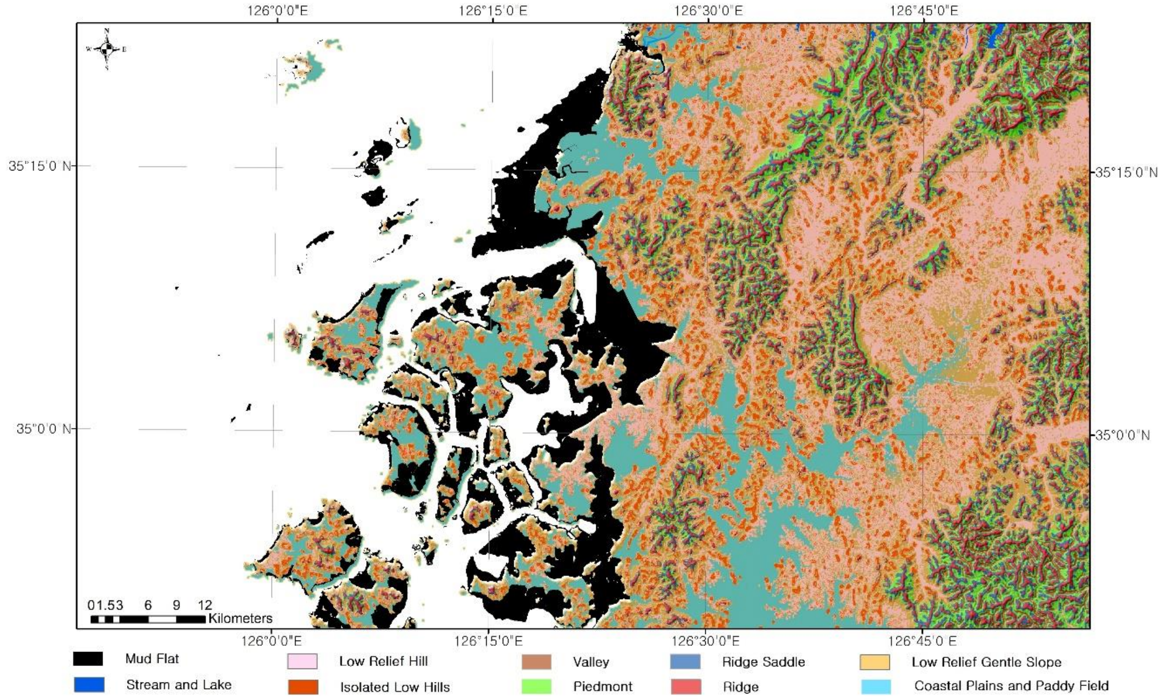

8. Category 3: Tidal flat, west coast of North Korea

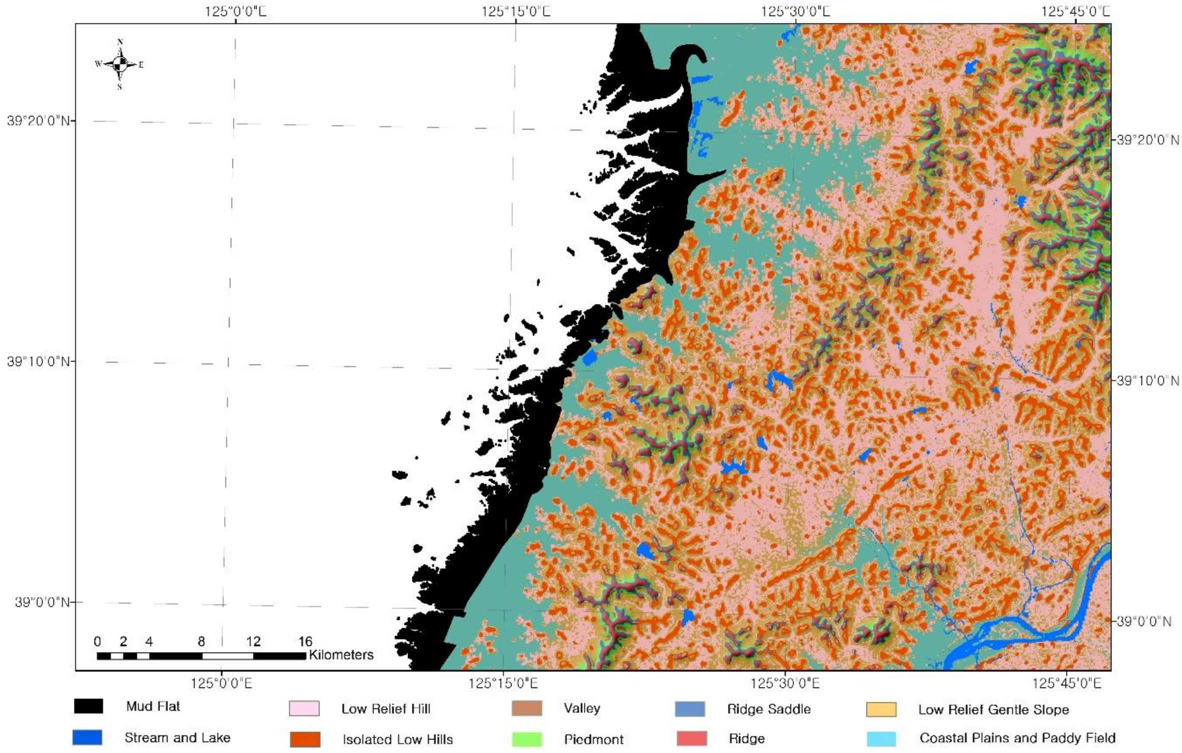

9. Category 4: Coastal Terrace, Jeongdongjin

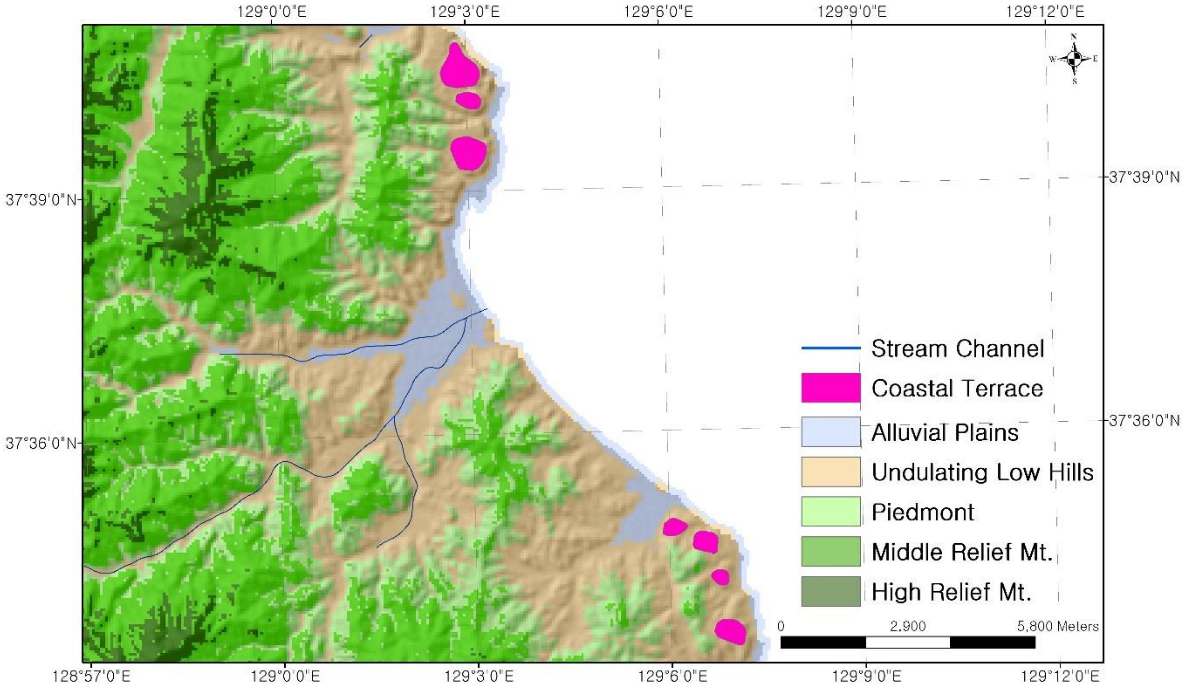

10. Category 4: Fluvial terrace

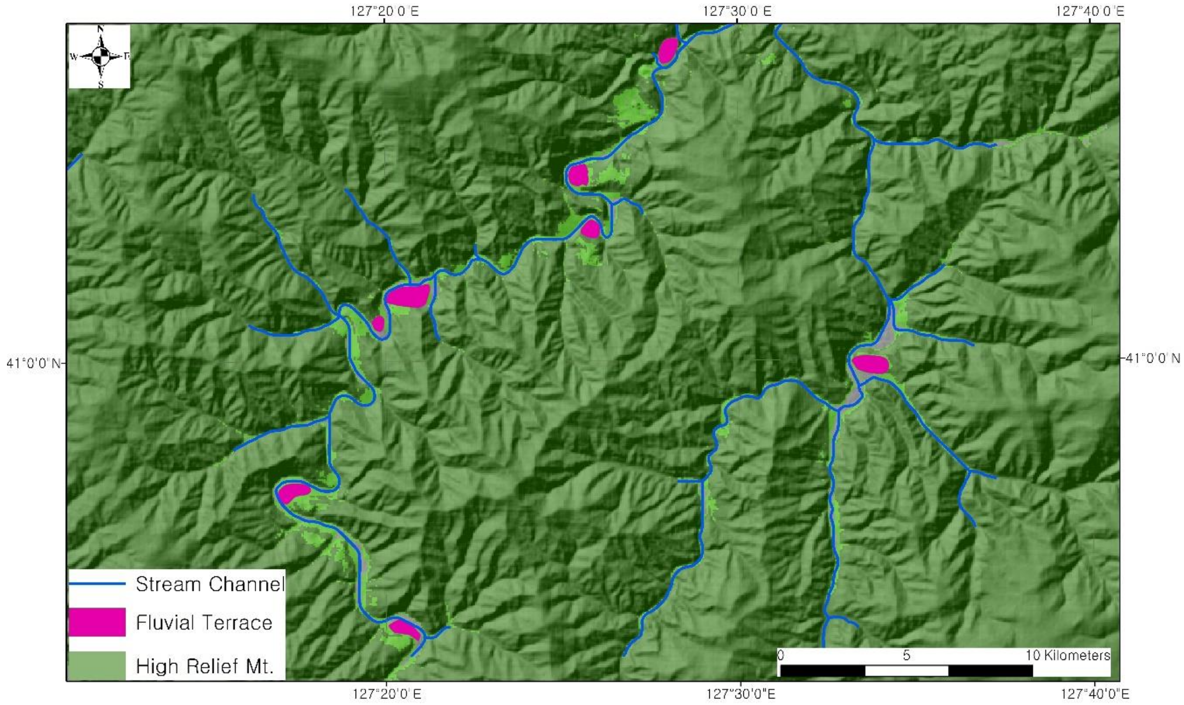

11. Category 4: Doline and Wetland

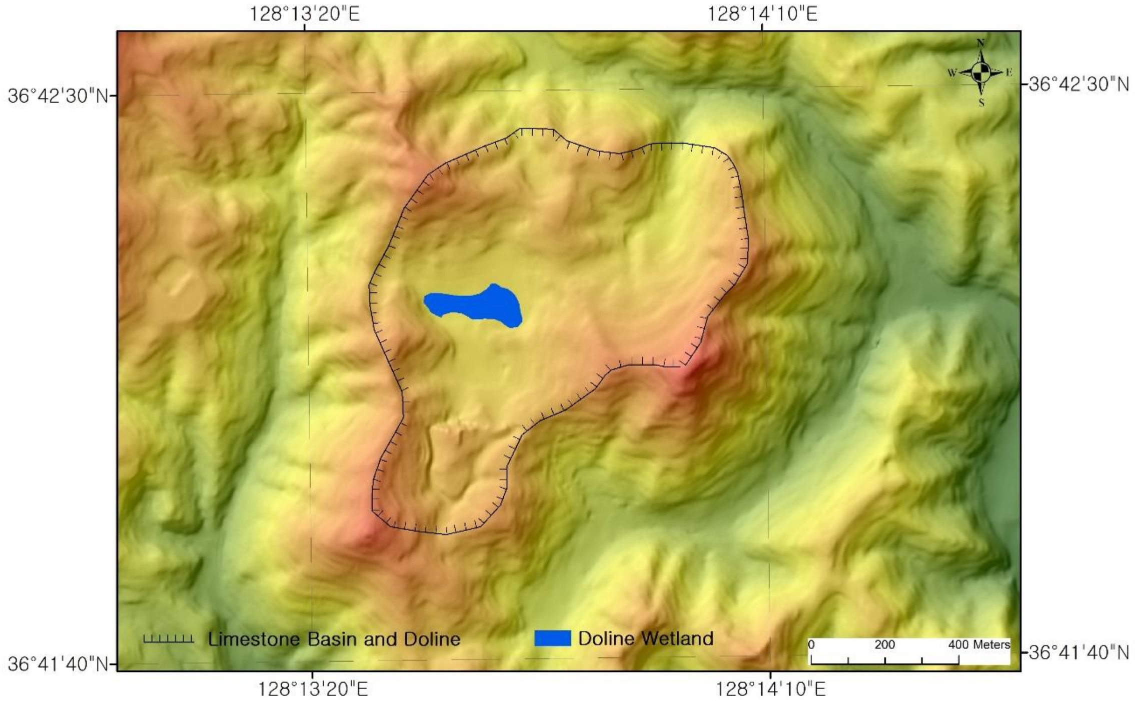

12. Category 4: Doline and Basin

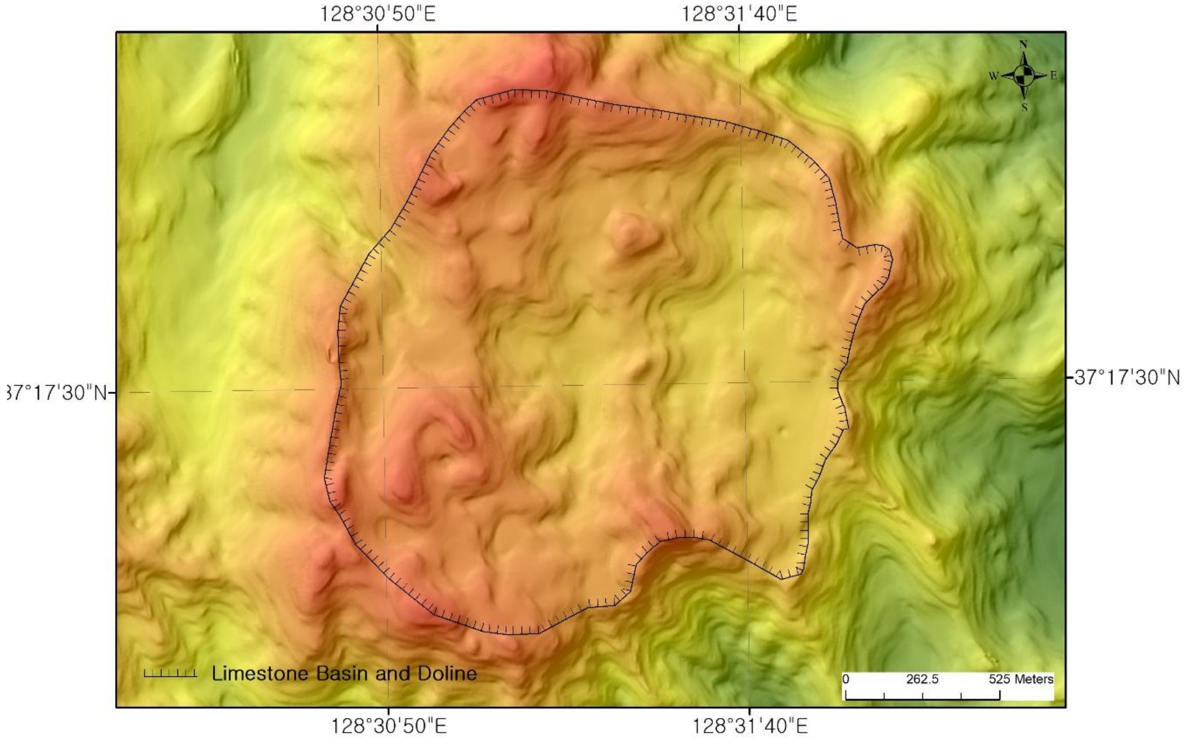

13. Category 4: Limestone Highland, Basin and Dolline

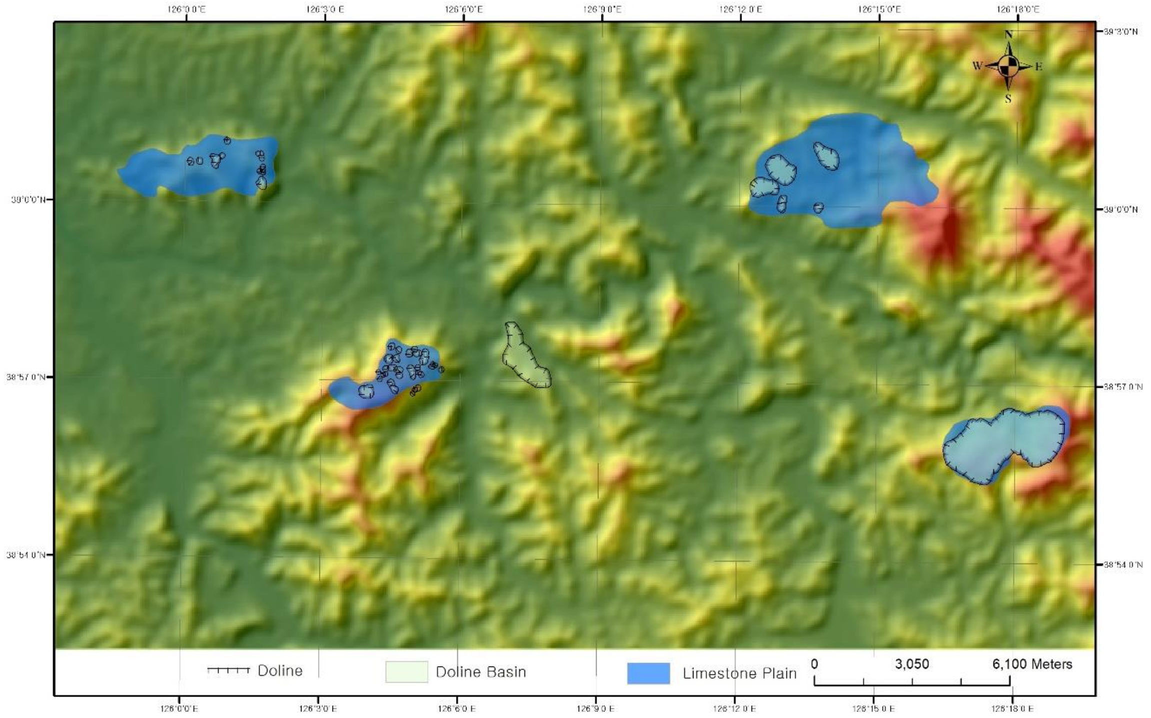

14. Category 4: Inland wetlands and Bar

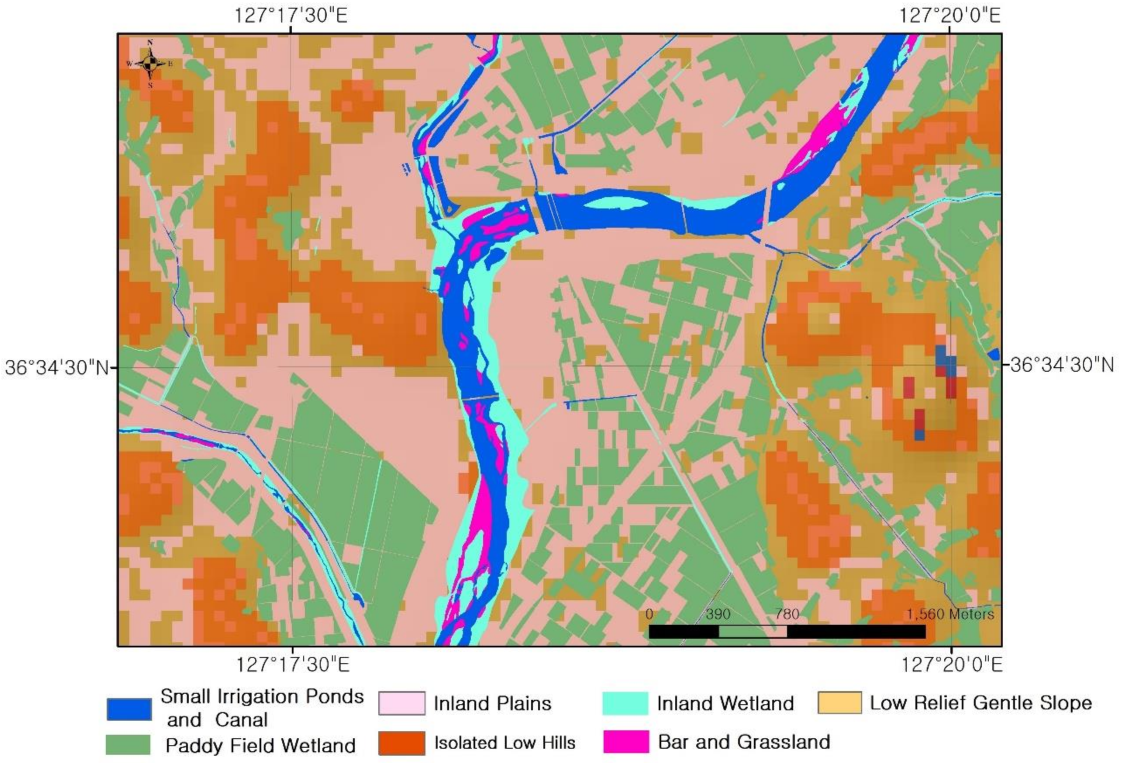

15. Category 4: Irrigation ponds, Channel, Paddy field wetland

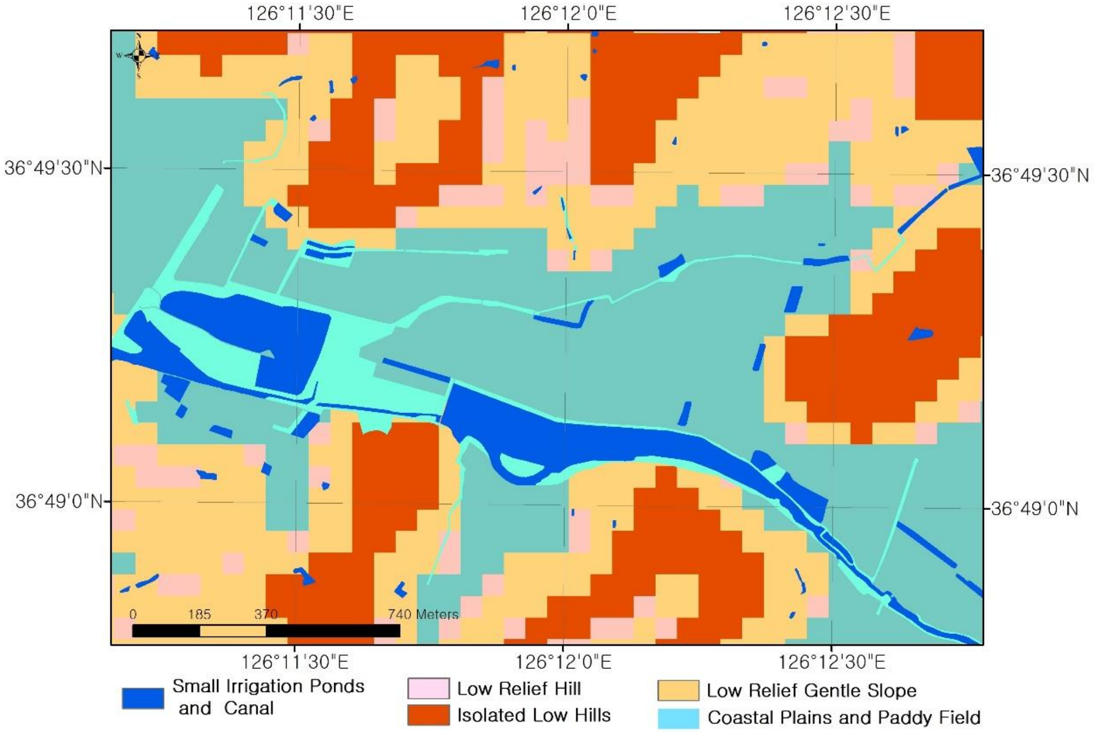

16. Category 4: Sandbar, Wetlands

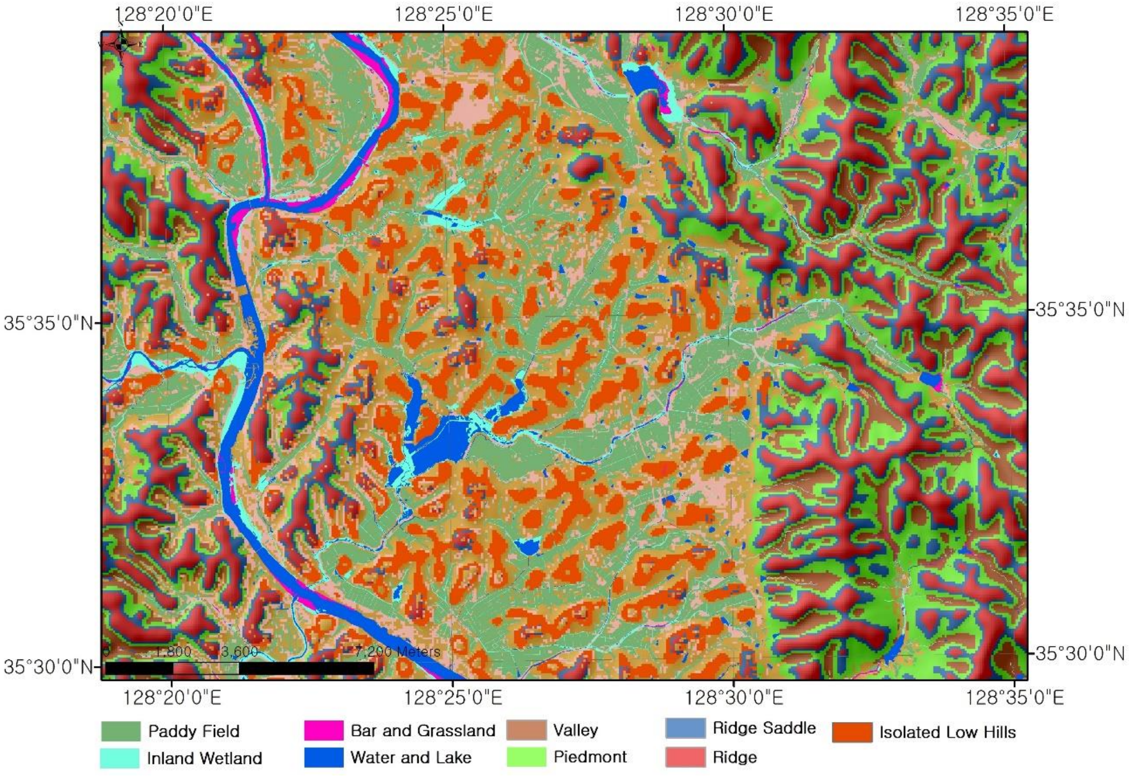

17. Category 4: Lagoon, Sand dune, Sand beach

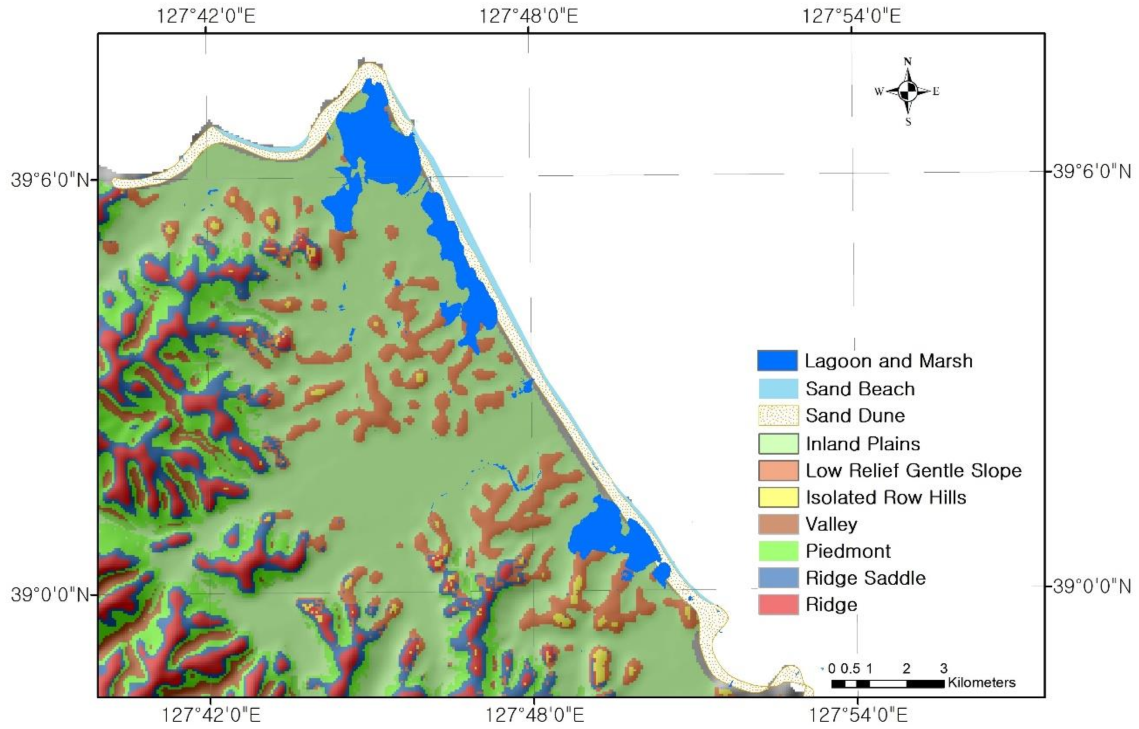

18. Category 4: Mountain wetlands, Gaema Plateau

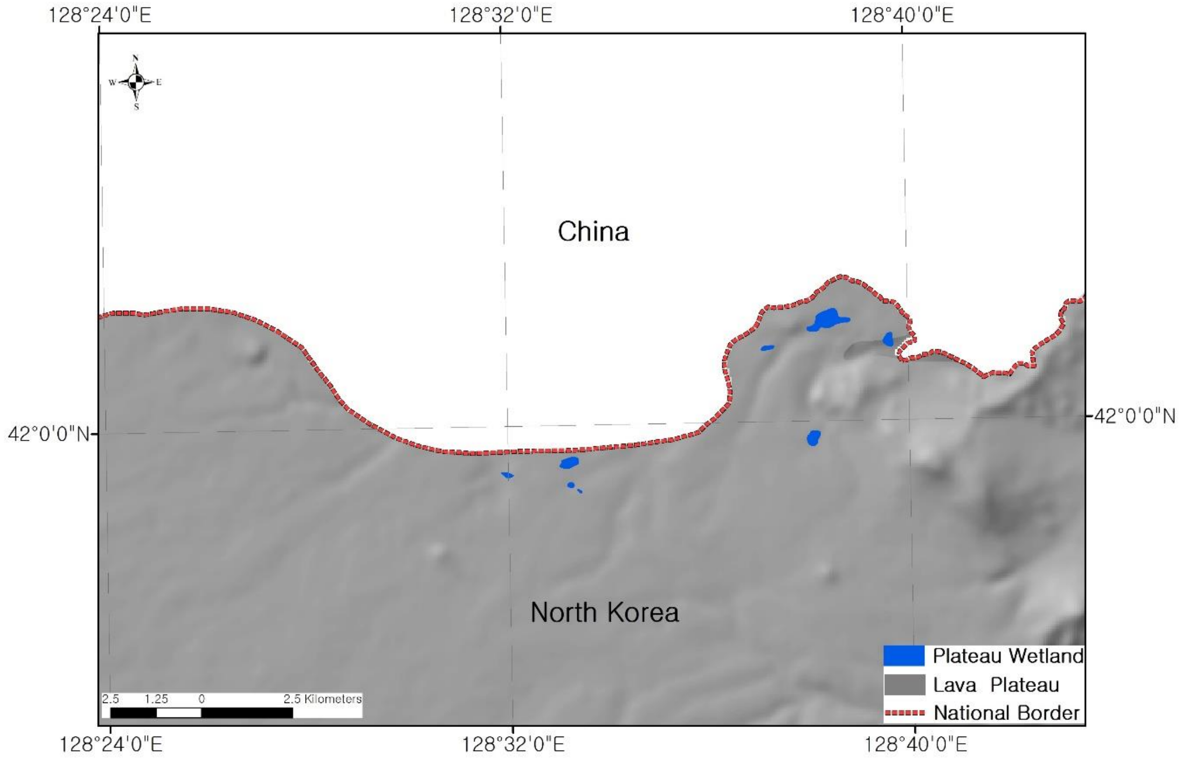

19. Category 4: Delta, Mouth of Nakdong

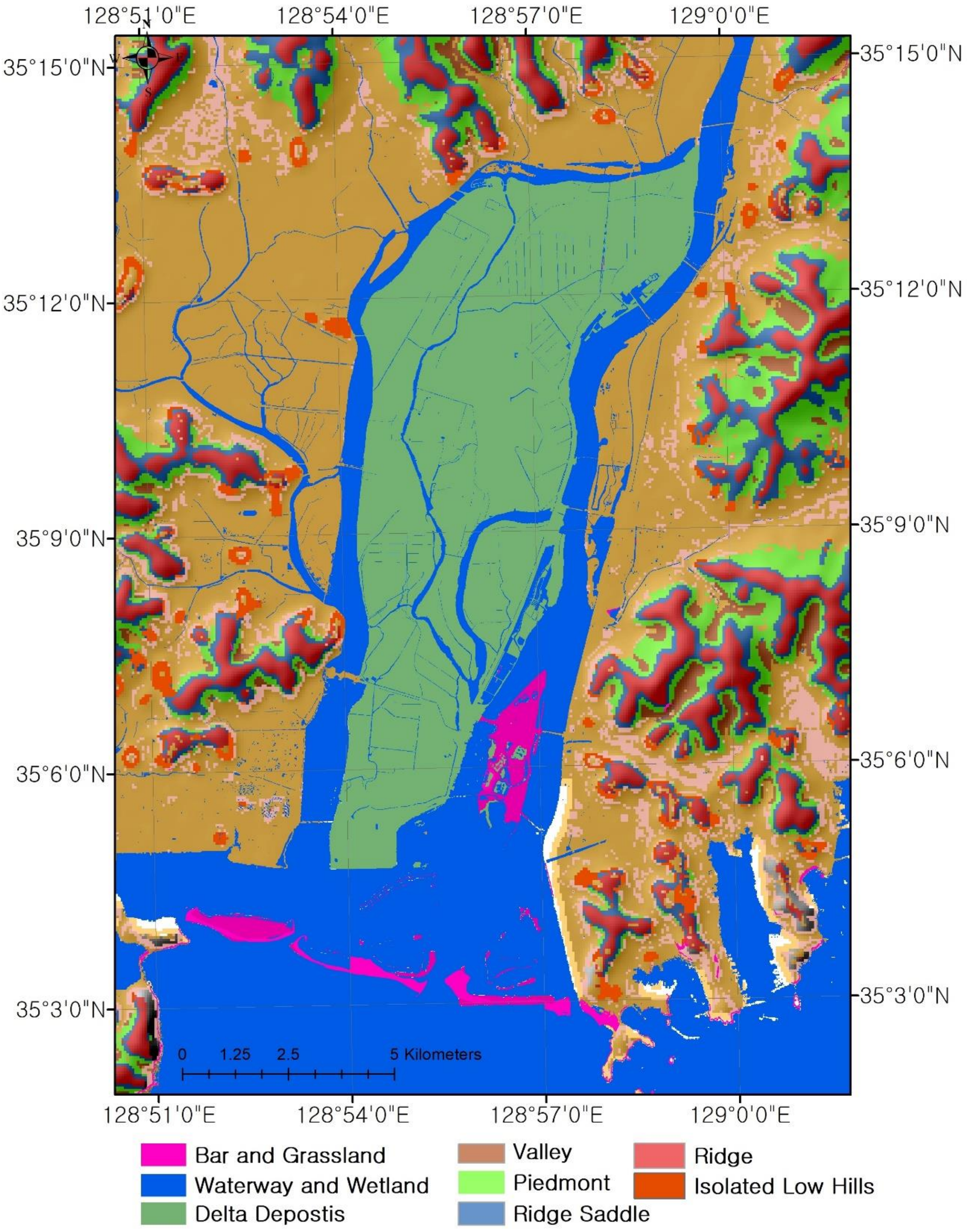

20. Category 4: Delta, Mouth of Duman

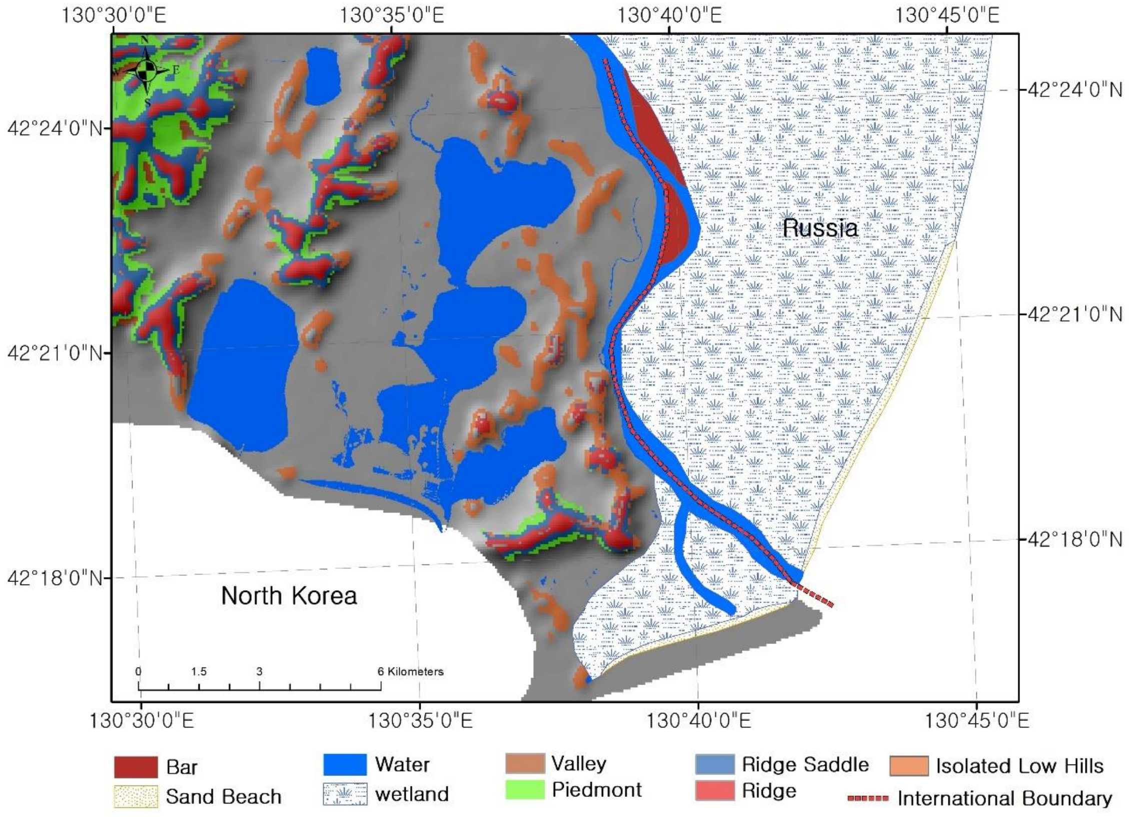

Habitats for biological Communities

6 Categories, 461 Habitats

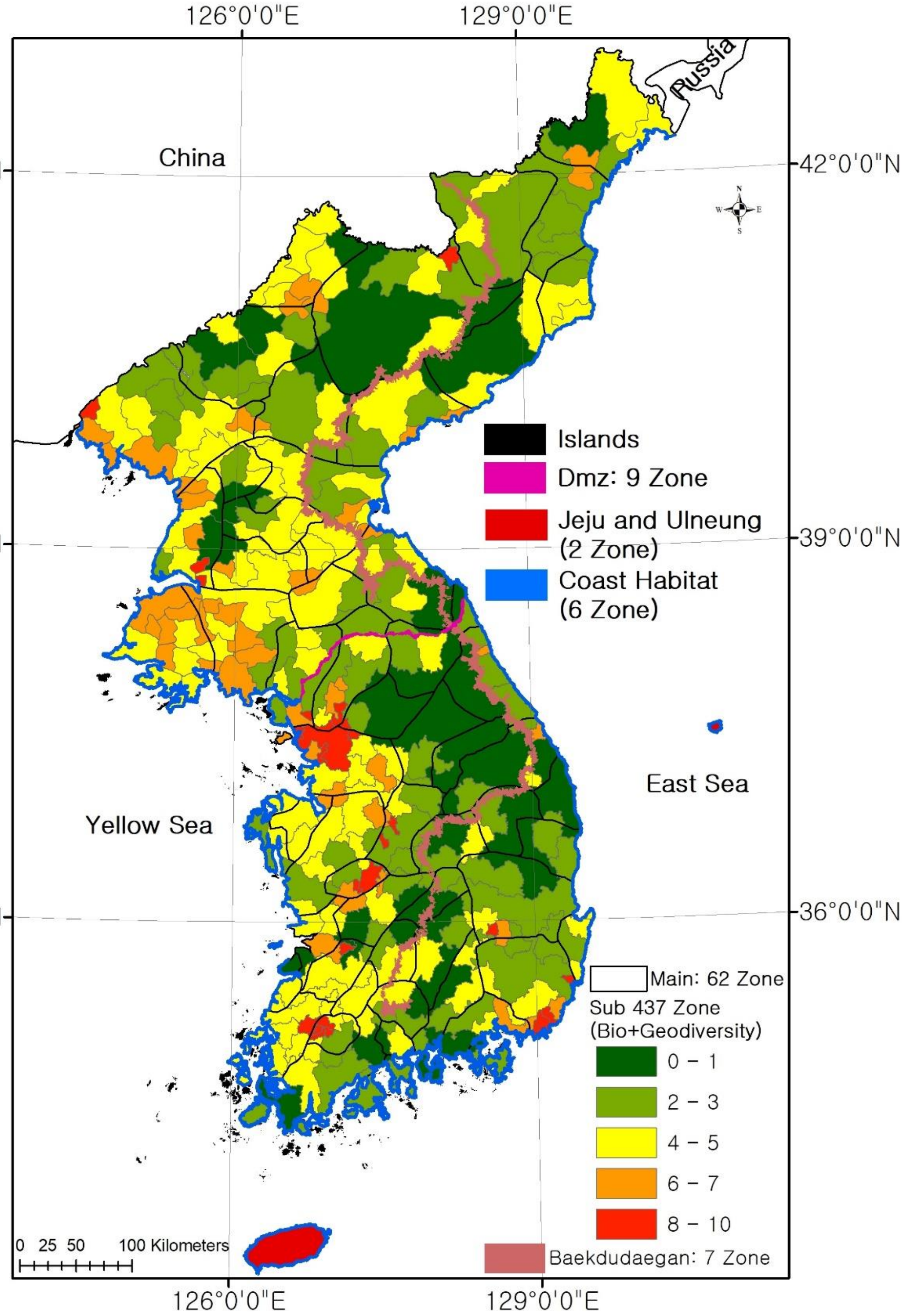

Supplement: S3 File — (PDF) [file pone.0259651.s003.pdf]
