# Supplementary material for: Hierarchical landform delineation for the habitats of biological communities on the Korean Peninsula
Source: PLoS One. 2021 Nov 5;16(11):e0259651. doi: 10.1371/journal.pone.0259651 (PMC8570509; doi:10.1371/journal.pone.0259651)
Supplement: S4 File — (PDF) [file pone.0259651.s004.pdf]

S4. Indicator species for each habitats.

Indicator Species	Significance level	
Group 10 Indicator species 7	stat	p.value
Echinosophora koreensis (Nakai) Nakai	0.387	0.005 **
Epimedium koreanum Nakai	0.344	0.010 **
Sanicula rubriflora F.Schmidt ex Maxim.	0.338	0.005 **
Polygonatum sibiricum F. Delaroche	0.308	0.030 *
Neillia uekii Nakai	0.307	0.005 **
Sedum zokuriense Nakai	0.280	0.020 *
Paeonia obovata Maxim.	0.258	0.010 **
Group 12 Indicator species 8		
Berberis koreana Palib.	0.379	0.005 **
Mukdenia rossii (Oliv.) Koidz.	0.370	0.005 **
Forsythia saxatilis (Nakai) Nakai	0.356	0.030 *
Anemone amurensis KOM.	0.356	0.025 *
Viburnum burejaeticum	0.290	0.010 **
Aconitum pseudolaeve Nakai	0.258	0.015 *
Cirsium setidens (Dunn) Nakai	0.247	0.020 *
Clematis trichotoma Nakai	0.239	0.010 **
Group 13 Indicator species 1		
Prunus takesimensis Nakai	0.365	0.025 *
Group 15 Indicator species 6		
Lonicera chrysantha Turcz. ex Ledeb.	0.707	0.045 *
Lathyrus vaniotii H.Lév.	0.707	0.045 *
Spiraea trichocarpa Nakai	0.707	0.045 *
Oplopanax elatus	0.707	0.045 *
Corydalis maculata B.U.Oh & Y.S.Kim	0.295	0.040 *
Asarum versicolor (K.Yamaki) Y.N.Lee	0.277	0.030 *
Group 16 Indicator species 9		
Asplenium ruta-muraria L.	0.775	0.005 **
Pulsatilla tongkangensis Y. N. Lee & T. C. Lee	0.632	0.030 *
Saussurea chabyoungsanica H. T. Im	0.600	0.005 **
A. coreana	0.456	0.005 **
Viola mirabilis var. subglabra L.	0.447	0.025 *
Saxifraga octopetala Nakai	0.359	0.020 *
Anemone koraiensis Nakai	0.321	0.005 **
Hypodematium glanduloso-pilosum (Tagawa) Ohwi	0.308	0.030 *
Lonicera subsessilis Rehder	0.284	0.015 *
Group 18 Indicator species 1		
Ajuga spectabilis Nakai	0.232	0.045 *
Group 19 Indicator species 1		
Megaleranthis saniculifolia Ohwi	0.32	0.005 **

Group 20 Indicator species 1		
Clematis brachyura Maxim.	0.252	0.025 *
Group 22 Indicator species 1		
Anemone reflexa Steph. ex Willd.	0.319	0.005 **
Group 23 Indicator species 1		
Delphinium maackianum Regel	0.313	0.02 *
Group 24 Indicator species 5		
Artemisia rubripes Nakai	0.309	0.005 **
Hosta minor (Baker) Nakai	0.306	0.005 **
Gueldenstaedtia verna (Georgi) Boriss.	0.303	0.030 *
Vicia chosenensis Ohwi	0.279	0.005 **
Iris koreana Nakai	0.259	0.035 *
Group 28 Indicator species 1		
Aconitum austrokoreense Koidz.	0.455	0.005 **
Group 30 Indicator species 1		
Ribes komarovii Pojark.	0.333	0.03 *
Group 31 Indicator species 1		
Dracocephalum rupestre Hance	0.707	0.025 *
Group 33 Indicator species 6		
Filipendula formosa Nakai	0.452	0.005 **
allopia koreana B.U.Oh & J.G.Kim	0.433	0.005 **
Parasenecio pseudotaimingasa (Nakai) B.U.Oh	0.433	0.005 **
Smilacina bicolor Nakai	0.408	0.005 **
Crepidiastrum koidzumianum (Kitam.) Pak & Kawano	0.387	0.005 **
Abies koreana	0.333	0.005 **
Group 34 Indicator species 1		
Coreanomecon hylomeconoides Nakai	0.315	0.01 **
Group 35 Indicator species 79		
Sasa palmata (Bean) E.G.Camus	1.000	0.005 **
Peracarpa carnosa var. circaeoides (F.Schmidt ex Miq.) Makino	1.000	0.005 **
Angelica japonica A.Gray	1.000	0.005 **
Pternopetalum tanakae (Franch. & Sav.) Hand.-Mazz.	0.894	0.005 **
Adonis multiflora Nishikawa & Koki Ito	0.894	0.005 **
Galeola septentrionalis Rchb.f.	0.894	0.005 **
Chionographis japonica (Willd.) Maxim.	0.894	0.005 **
Maackia fauriei (H.Lév.) Takeda	0.894	0.005 **
Strobilanthes oliganthus Miq.	0.894	0.005 **
Damnacanthus indicus C.F.Gaertn.	0.894	0.005 **
Gentiana scabra Bunge	0.866	0.005 **
Artemisia japonica var. hallaisanensis (Nakai) Kitam.	0.866	0.005 **
Schisandra repanda (Siebold & Zucc.) Radlk.	0.866	0.005 **
Wedelia prostrata Hemsl.	0.816	0.005 **

Aruncus aethusifolius (H.Lev.) Nakai	0.816	0.005 **
Pteris cretica L.	0.756	0.005 **
Cirsium rhinoceros (H.Lev. & Vaniot) Nakai	0.756	0.005 **
Mercurialis leiocarpa Siebold & Zucc.	0.756	0.005 **
Rhynchospermum verticillatum Reinw.	0.756	0.005 **
Euphorbia pekinensis Rupr.	0.750	0.005 **
Dendranthema coreanum (H.Lev. & Vaniot) Vorosch.	0.707	0.015 *
Microlepia strigosa (Thunb.) C.Presl	0.707	0.005 **
Rhododendron weyrichii Maxim.	0.707	0.005 **
Daphne kiusiana Miq.	0.671	0.005 **
Aster arenarius (Kitam.) Nemoto	0.671	0.005 **
Desmodium caudatum (Thunb.) DC.	0.667	0.005 **
Pollia japonica Thunb.	0.632	0.005 **
Cimicifuga biternata (Siebold & Zucc.) Miq.	0.612	0.005 **
Cardamine glechomifolia H.Lev.	0.612	0.005 **
Ranunculus crucilobus H.Lev.	0.612	0.005 **
Ligustrum foliosum Nakai	0.577	0.005 **
Rhamnus taquetii (H.Lev. & Vaniot) H.Lev.	0.567	0.005 **
Cleyera japonica Thunb.	0.555	0.005 **
Ficus erecta var. sieboldii (Miq.) King	0.555	0.005 **
Wahlenbergia marginata (Thunb.) A.DC.	0.535	0.005 **
Distylium racemosum Siebold & Zucc.	0.535	0.005 **
Neolitsea aciculata (Blume) Koidz.	0.535	0.005 **
Zanthoxylum ailanthoides Siebold & Zucc.	0.516	0.005 **
Ardisia crenata Sims	0.516	0.005 **
Elaeagnus submacrophylla Servett.	0.516	0.005 **
Ligularia taquetii (H.Lev. & Vaniot) Nakai	0.500	0.030 *
Berberis amurensis var. quelpaertensis (Nakai) Nakai	0.474	0.005 **
Cimicifuga japonica (Thunb.) Spreng.	0.471	0.005 **
Ophiopogon jaburan (Siebold) Lodd.	0.471	0.005 **
Idesia polycarpa Maxim.	0.471	0.005 **
Verbena officinalis	0.459	0.005 **
Machilus japonica Siebold & Zucc.	0.459	0.005 **
Eurya emarginata (Thunb.) Makino	0.459	0.005 **
Actinodaphne lancifolia (Siebold & Zucc.) Meisn.	0.459	0.005 **
Corydalis decumbens (Thunb.) Pers.	0.447	0.005 **
Ainsliaea apiculata Sch.Bip.	0.447	0.005 **
Sageretia thea (Osbeck) M. C. Johnst.	0.436	0.005 **
Viburnum odoratissimum var. awabuki (K.Koch) Zabel ex Rumpler	0.436	0.005 **
Primula modesta var. hannasanensis T.Yamaz.	0.433	0.005 **
Quercus acuta Thunb.	0.417	0.005 **
Ostrya japonica Sarg.	0.401	0.010 **

Dryopteris fuscipes C.Chr.	0.400	0.005 **
Castanopsis sieboldii (Makino) Hatus.	0.400	0.005 **
Litsea japonica (Thunb.) Juss.	0.400	0.005 **
Rhaphiolepis indica var. umbellata (Thunb.) Ohashi	0.400	0.005 **
Diplopterygium glaucum (Thunb. ex Houtt.) Nakai	0.392	0.005 **
Caesalpinia decapetala (Roth) Alston	0.392	0.005 **
Quercus myrsinifolia Blume	0.392	0.005 **
Kadsura japonica (L.) Dunal	0.392	0.005 **
Cinnamomum yabunikkei H.Ohba	0.392	0.005 **
Quercus glauca	0.378	0.005 **
Aster hayatae H.Lev. & Vaniot	0.371	0.005 **
Messerschmidia sibirica	0.365	0.005 **
Elaeagnus glabra Thunb.	0.365	0.005 **
Boehmeria pannosa Nakai & Satake	0.365	0.005 **
Cnidium japonicum Miq.	0.365	0.005 **
Ficus erecta	0.348	0.005 **
Rubus hirsutus Thunb.	0.343	0.005 **
Ilex crenata Thunb.	0.338	0.005 **
Asarum maculatum Nakai	0.338	0.005 **
Pittosporum tobira (Thunb.) W.T.Aiton	0.320	0.005 **
Juncus setchuensis var. effusoides Buchenau	0.306	0.015 *
Artemisia dubia Wall.	0.270	0.010 **
Rhynchosia volubilis Lour.	0.263	0.030 *
Group 40 Indicator species 2		
Lycopodium clavatum var. nipponicum	0.500	0.015 *
Astilboides tabularis (Hemsl.) Engl.	0.385	0.010 **
Group 41 Indicator species 6		
Vaccinium bracteatum Thunb.	0.566	0.005 **
Callicarpa mollis Siebold & Zucc.	0.430	0.005 **
Indigofera kirilowii f. albiflora Uyeki	0.369	0.005 **
Mallotus japonicus (L.f.) Müll.Arg.	0.349	0.005 **
Bletilla striata (Thunb.) Rchb.f.	0.298	0.040 *
Millettia japonica (Siebold & Zucc.) A.Gray	0.296	0.035 *
Group 46 Indicator species 2		
Rubus ribisoideus Matsum.	0.333	0.03 *
Pinellia tripartita (Blume) Schott	0.320	0.04 *
Group 47 Indicator species 4		
Lespedeza maritima Nakai	0.311	0.010 **
Vicia hirticalycina Nakai	0.305	0.005 **
Ligustrum japonicum Thunb.	0.290	0.005 **
Deutzia paniculata Nakai	0.277	0.035 *
Group 49 Indicator species 1		

Goodyera velutina Maxim. ex Regel	0.816	0.005 **
Group 51 Indicator species 1		
Fimbristylis dichotoma (L.) Vahl	0.577	0.02 *
Group 54 Indicator species 1		
Scutellaria insignis Nakai	0.289	0.01 **
Group 56 Indicator species 1		
Dunbaria villosa (Thunb.) Makino	0.269	0.01 **
Group 57 Indicator species 24		
Veronica kiusiana var. diamantiaca (Nakai) T.Yamaz.	0.676	0.005 **
Androsace cortusaefolia Nakai	0.632	0.005 **
Bupleurum euphorbioides Nakai	0.539	0.005 **
Hanabusaya asiatica (Nakai) Nakai	0.527	0.005 **
Aristolochia manshuriensis Kom.	0.447	0.025 *
Patrinia rupestris (Pall.) Juss.	0.415	0.005 **
Acer ukurunduense Trautv. & C.A.Mey.	0.408	0.005 **
Rosa koreana Kom.	0.405	0.010 **
Forsythia ovata Nakai	0.405	0.005 **
Scabiosa tschiliensis Gruning	0.402	0.005 **
Menyanthes trifoliata	0.400	0.040 *
Leontopodium japonicum Miq.	0.390	0.005 **
Clematis fusca var. coreana (H.Lev.) Nakai	0.390	0.005 **
Salvia chanryoenica Nakai	0.378	0.005 **
Cardamine flexuosa	0.358	0.005 **
Acer tegmentosum	0.344	0.005 **
Lilium cernuum Kom.	0.341	0.005 **
Thalictrum rochebrunianum var. grandisepalum (H.Lév.) Nakai	0.327	0.005 **
Saxifraga punctata L.	0.316	0.030 *
Viola diamantiaca Nakai	0.307	0.005 **
Saussurea macrolepis (Nakai) Kitam.	0.253	0.010 **
Scopolia japonica Maxim.	0.250	0.020 *
Cirsium pendulum Fisch. ex DC.	0.244	0.035 *
Scrophularia koraiensis Nakai	0.224	0.045 *
Group 58 Indicator species 12		
Melampyrum roseum var. hirsutum Beauverd	0.707	0.005 **
Lentinus lepideus (Fr.)Fr.	0.707	0.005 **
A. rufinerve Nak.	0.707	0.005 **
Potentilla fruticosa var. mandshurica Maxim.	0.707	0.010 **
Betula fusenensis	0.707	0.005 **
Picea jezoensis (Siebold & Zucc.) Carrière	0.707	0.005 **
Betula microphylla var. coreana	0.707	0.005 **
Sorbus alnifolia (Siebold & Zucc.) K.Koch	0.612	0.005 **
Salix orthostemma	0.612	0.005 **

Cardamine trifida (Lam. ex Poir.)	0.577	0.010 **
Lycopodium alpinum L.	0.577	0.010 **
Euphrasia retrotricha	0.365	0.035 *
Group 59 Indicator species 12		
Dendropanax morbiferus H.Lév.	0.440	0.005 **
Rubus corchorifolius L.f.	0.371	0.005 **
Rhus succedanea L.	0.362	0.005 **
Nanocnide japonica Blume	0.360	0.005 **
Neolitsea sericea (Blume) Koidz.	0.352	0.005 **
Lindera sericea (Siebold & Zucc.) Blume	0.336	0.005 **
Pteris multifida Poir.	0.318	0.005 **
Rhus sylvestris Siebold & Zucc.	0.316	0.005 **
Meliosma myriantha Siebold & Zucc.	0.303	0.005 **
Chloranthus fortunei (A.Gray) Solms	0.296	0.005 **
Arisaema ringens (Thunb.) Schott	0.286	0.005 **
Hepatica insularis Nakai	0.285	0.005 **
Group 501 Indicator species 5		
Lycoris uydoensis M.Y.Kim	1.000	0.010 **
Hosta yingeri S.B.Jones	1.000	0.010 **
Euonymus chibai Makino	0.577	0.040 *
Dumasia truncata Siebold & Zucc.	0.577	0.015 *
Silene capitata Kom.	0.408	0.050 *
