# Supplementary material for: Hierarchical landform delineation for the habitats of biological communities on the Korean Peninsula
Source: PLoS One. 2021 Nov 5;16(11):e0259651. doi: 10.1371/journal.pone.0259651 (PMC8570509; doi:10.1371/journal.pone.0259651)
Supplement: S5 File — (PDF) [file pone.0259651.s005.pdf]

S5. Conservation grade of landform for each scale.

<table>
<tr><th>Category 1</th><th rowspan="2">Grade</th><th>Category 2</th><th rowspan="2">Grade</th><th>Category 3</th><th rowspan="2">Grade</th><th>Category 4</th><th rowspan="2">Grade</th></tr>
<tr><th>1:5,000,000</th><th>1:1,000,000</th><th>1: 50,000 ~ 25,000</th><th>Under 1:5,000</th></tr>
<tr><td rowspan="20">Mountains</td><td rowspan="20">Ⅰ,Ⅱ</td><td rowspan="6">High Elevation Mt.</td><td rowspan="6">Ⅰ</td><td rowspan="2">Mt. Granite</td><td rowspan="2">Ⅰ</td><td>Magma Plateau</td><td>Ⅰ</td></tr>
<tr><td rowspan="2">Highland Flat</td><td rowspan="2">Ⅰ</td></tr>
<tr><td rowspan="2">Mt. Gneiss Series</td><td rowspan="2">Ⅰ</td></tr>
<tr><td rowspan="2">Karst Basin</td><td rowspan="2">Ⅰ</td></tr>
<tr><td rowspan="3">Mt. Tertiary Layer</td><td rowspan="2">Ⅱ</td></tr>
<tr><td rowspan="3">Karst Flat</td><td rowspan="3">Ⅰ</td></tr>
<tr><td rowspan="8">Middle Elevation Mt.</td><td rowspan="9">Ⅰ</td><td rowspan="3">Ⅰ</td></tr>
<tr><td rowspan="2">Mt. Limestone</td></tr>
<tr><td rowspan="2">Wetland Mt.</td><td rowspan="2">Ⅰ</td></tr>
<tr><td rowspan="2">Mt. Pyroclast</td><td rowspan="3">Ⅰ</td></tr>
<tr><td rowspan="2">Wind Hole</td><td rowspan="2">Ⅰ</td></tr>
<tr><td rowspan="4">Volcanic Mt.</td></tr>
<tr><td rowspan="3">Ⅰ</td><td>Piedmont</td><td>Ⅱ (landuse Ⅲ)</td></tr>
<tr><td rowspan="3">Ridge</td><td rowspan="3">Ⅰ</td></tr>
<tr><td rowspan="6">Piedmont</td></tr>
<tr><td rowspan="5">Ⅱ (landuse Ⅲ)</td><td rowspan="2">Lava Plateau</td><td rowspan="2">Ⅰ</td></tr>
<tr><td rowspan="2">Valley</td><td rowspan="2">Ⅰ</td></tr>
<tr><td rowspan="2">High Flat</td><td rowspan="2">Ⅰ</td></tr>
<tr><td rowspan="2">Ridge Saddle</td><td rowspan="2">Ⅰ</td></tr>
<tr><td>Limestone Basin</td><td>Ⅰ</td></tr>
</table>

<table>
<tr><td rowspan="16"></td><td rowspan="16"></td><td></td><td></td><td rowspan="2"></td><td rowspan="3"></td><td rowspan="3">Rock Block</td><td rowspan="3">Ⅰ</td></tr>
<tr><td rowspan="8">Drainage Divide</td><td rowspan="8">Ⅰ</td></tr>
<tr><td rowspan="2">Isolated Mountain</td></tr>
<tr><td rowspan="2">Ⅰ</td><td rowspan="2">Mountain Cliff</td><td rowspan="2">Ⅰ</td></tr>
<tr><td rowspan="3">Drainage Divide</td></tr>
<tr><td rowspan="2">Ⅰ</td><td>Crator</td><td>Ⅰ</td></tr>
<tr><td rowspan="2">Mountain Bog</td><td rowspan="2">Ⅰ</td></tr>
<tr><td rowspan="3">Water and Lake</td><td rowspan="3">Ⅰ,Ⅲ</td></tr>
<tr><td rowspan="3">Doline Wetland</td><td rowspan="3">Ⅰ</td></tr>
<tr><td rowspan="7">Water and Lake</td><td rowspan="7">Ⅰ</td></tr>
<tr><td rowspan="2">Wetland in Lake</td><td rowspan="2">Ⅰ</td></tr>
<tr><td rowspan="2">Drainage Divide</td><td rowspan="2">Ⅰ</td></tr>
<tr><td rowspan="2">Inland Erosion Basin</td><td rowspan="2">Ⅲ</td></tr>
<tr><td rowspan="2">Water and Lake</td><td rowspan="2">Ⅰ,Ⅲ</td></tr>
<tr><td rowspan="2">Caldera Basin</td><td rowspan="2">Ⅰ</td></tr>
<tr><td>Wetland in Lake</td><td>Ⅰ</td></tr>
<tr><td rowspan="8">Plains</td><td rowspan="8">Ⅱ, Ⅲ</td><td rowspan="5">Alluvial Plains</td><td rowspan="6">Ⅲ</td><td rowspan="3">Coastal Plains</td><td rowspan="3">Ⅲ</td><td>Bar and Grassland</td><td>Ⅱ</td></tr>
<tr><td>Waterway and Wetland</td><td>Ⅱ</td></tr>
<tr><td rowspan="2">Small irrigation pond and Canal</td><td rowspan="2">Ⅱ</td></tr>
<tr><td rowspan="3">Inland Plains</td><td rowspan="3">Ⅲ</td></tr>
<tr><td rowspan="3">Small Inland Plains</td><td rowspan="3">Ⅲ</td></tr>
<tr><td rowspan="3">Undulating Hills</td></tr>
<tr><td rowspan="2">Ⅱ (landc over</td><td rowspan="2">Delta Plains</td><td rowspan="2">Ⅱ</td></tr>
<tr><td>Small</td><td>Ⅲ</td></tr>
</table>

			Ⅲ)			Coastal Plains	
						Inland Wetland	Ⅰ
				Water and Lake	Ⅱ	Isolated Low Hills	Ⅱ
						Low Relief Gentle Slope	Ⅲ
				Wetland in Lake	Ⅰ	Paddy Field Wetland	Ⅲ
				Low Gentle Slope	Ⅲ	Water and Lake	Ⅱ,Ⅲ
						Wetland in Lake	Ⅱ
Fluvial Landform	Ⅰ, Ⅱ	Incised Meander Stream	Ⅰ	Rocky Channel	Ⅰ	River Terrace	Ⅱ
						Alluvial Island	Ⅲ
				Sand and Gravel Channel	Ⅰ	Riparian Wetland	Ⅱ
						Riverside Wetland	Ⅱ
				Silt and Mud Channel	Ⅱ,Ⅲ	Braided Stream Channel	Ⅱ
						Bar	Ⅱ
		Meander Stream	Ⅱ	Water and Lake	Ⅱ	Riverside Land	Ⅱ
						Stream and Lake	Ⅱ,Ⅲ
				Wetland in Lake	Ⅰ	Wetland in Lake	Ⅱ
						Fluvial Cliff	Ⅰ
Coastal Landform	Ⅰ, Ⅱ,Ⅲ	Uplift Coast	Ⅰ	Rocky Coast	Ⅰ	Coastal Terrace	Ⅰ
						Rocky Beach	Ⅰ
				Sand Coast	Ⅰ	Sand Beach	Ⅰ

						Sand and Mud Beach	Ⅱ
						Sand and Gravel Beach	Ⅰ
				Sand and Mud Coast	Ⅱ	Tidalflat	Ⅰ
						Mixed Coast	Ⅱ
		Rias Coast	Ⅰ (landcover Ⅱ,Ⅲ)	Sand and Gravel Coast	Ⅱ	Sandune Wetland	Ⅰ
						Sand Dune	Ⅰ
						Salt Marsh	Ⅰ
				Mud Coast	Ⅰ	Lagoon	Ⅰ
		Volcanic Coast	Ⅱ(land cover Ⅲ)			Coastal Cliff	Ⅰ
				Mixed Coast	Ⅱ	Headland	Ⅱ
						Mud Beach	Ⅰ
						Salt Marsh	Ⅰ
		Island	Ⅰ (landcover Ⅲ)	Island	Ⅰ (landcover Ⅲ)	Sand Beach	Ⅰ
						Sanddune	Ⅰ
Island	Ⅰ					Lagoon	Ⅰ
						Tidal Flat	Ⅰ
		Volcanic Island	Ⅰ (landcover Ⅲ)	Volcanic Island	Ⅰ (landcover Ⅲ)	Cliff	Ⅰ
						Coastal Terrace	Ⅰ
Baekdudaegan	Ⅰ	Baekdudaegan	Ⅰ	Baekdudaegan	Ⅰ	Baekdudaegan Ecozone	Ⅰ
DMZ	Ⅰ	DMZ	Ⅰ	DMZ	Ⅰ	DMZ Ecozone	Ⅰ
