# Supplementary material for: Hierarchical landform delineation for the habitats of biological communities on the Korean Peninsula
Source: PLoS One. 2021 Nov 5;16(11):e0259651. doi: 10.1371/journal.pone.0259651 (PMC8570509; doi:10.1371/journal.pone.0259651)
Supplement: S1 Data — (PDF) [file pone.0259651.s006.pdf]

Supplementary data 1. Major Landforms for Habitats in Korea Peninsular

Landforms	Major Types	
mountains	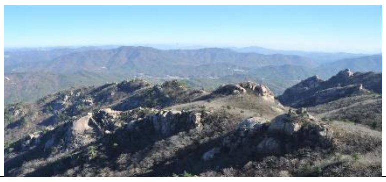	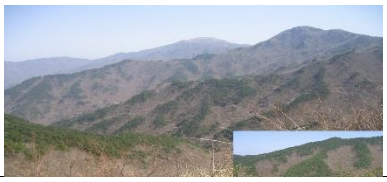
	Granite Mountain	Gneiss Mountain
Plateau and Flat	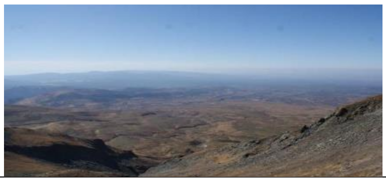	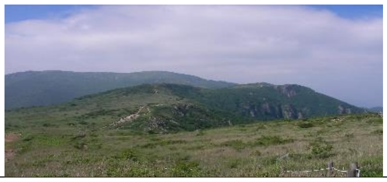
	Magma Plateau	High Flat
Basin	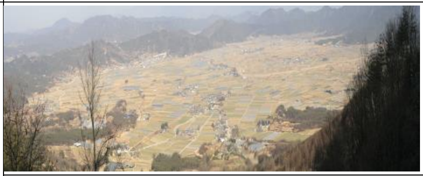	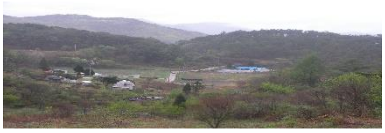
	Inland Erosional Basin	Limestone Basin
Piedmont	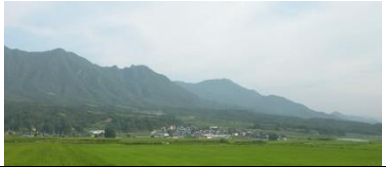	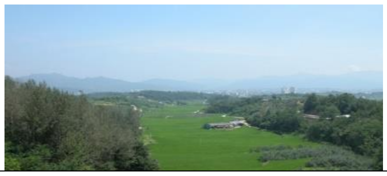
	Piedmont	Flat Valley in Piedmont
Rock Block and Cliff	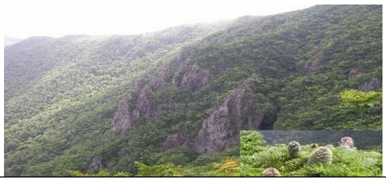	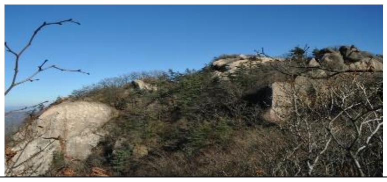
	Mountain Cliff	Rock Block
Wind hole	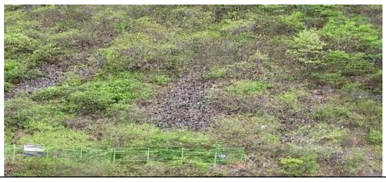	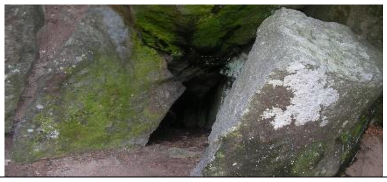
	Algific Talus	Wind Hole
Caldera	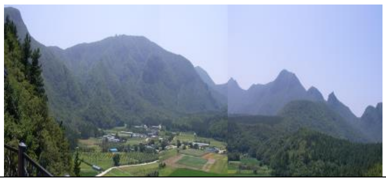	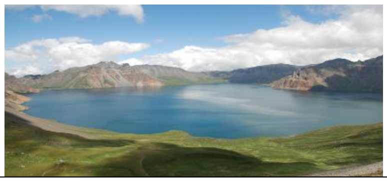
	Caldera(Ulneung island)	Caldera(Cheonji Mt, Baekdu)
	Caldera	

Coastal Landforms and Delta	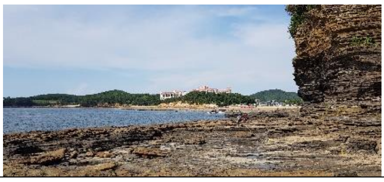	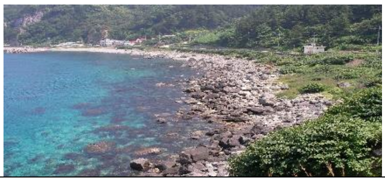
	Rock Beach	Bounder Beach
	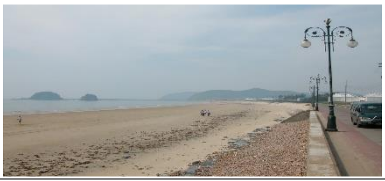	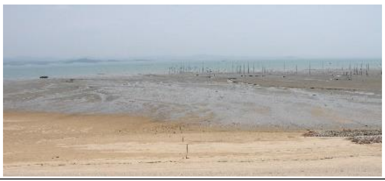
	Sand Beach	Mud and Sand Beach
	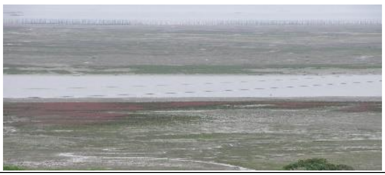	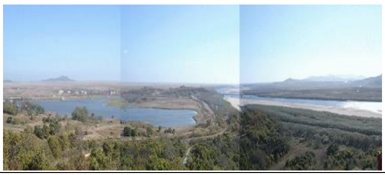
	Mudflat Beach	Delta
	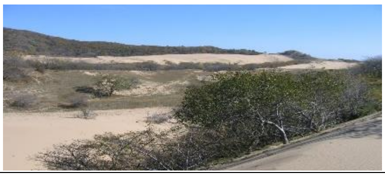	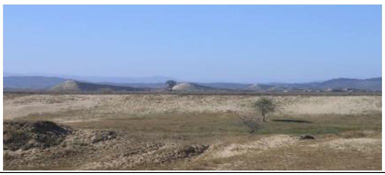
	Sand dune	Sand dune
Mountain Bog and Wetland	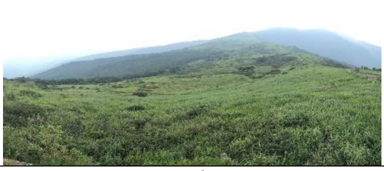	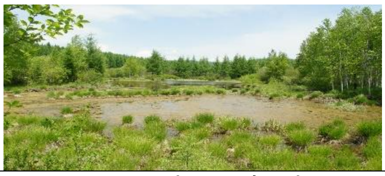
	Mountain Bog	Mountain Wetland
Irrigation Pond	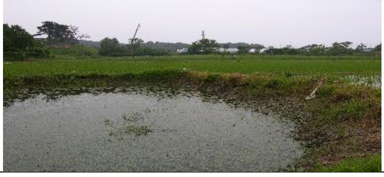	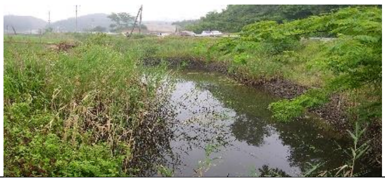
	Small Irrigation Pond	
Irrigation Canal	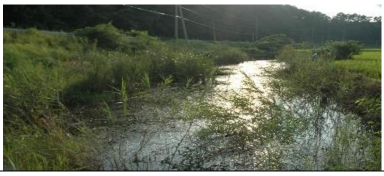	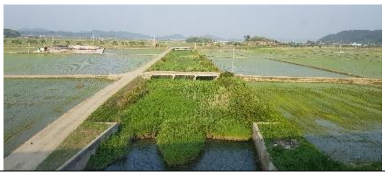
	Irrigation Canal	
Fluvial Landforms	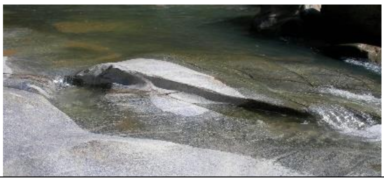	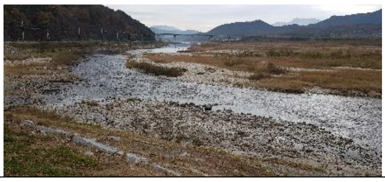
	Rocky Channel	Sand and Gravel Channel

	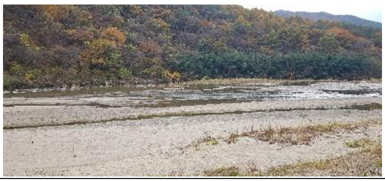	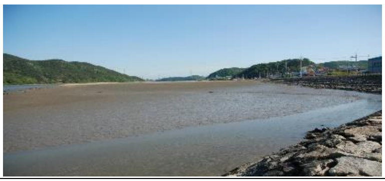
	Sand Channel	Silt and Mud Channel
Stream and Paddy Field Wetland	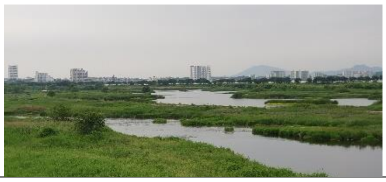	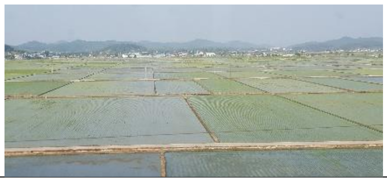
	Inner Wetland in Stream	Paddy Field Wetland
Lake Wetland	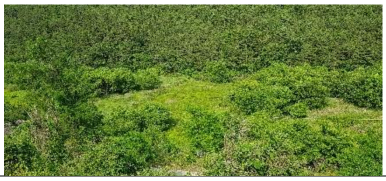	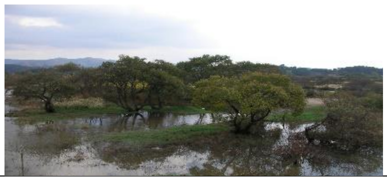
	Tributary Inflow Wetland in Lake	Tributary Inflow Wetland in Lake
Baekdudaegan and DMZ	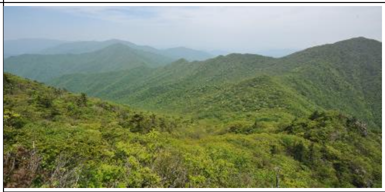	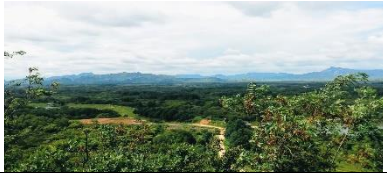
	Baekdudaegan	DMZ Ecozone

Supplementary data 2. Landform Classification Procedures by Categories

Category_1

① Mountain and Plains

Category_1	Classification	
**1:5,000,000**	**Relief**	**Elevation**
Mountains	91 <= Relief < 1,200	elev >= 100
Plains	0 <= Relief < 91	elev < 100

**Map algebra**	
Mountain	*(91 <= Relief < 1,200) and (elev >= 100)*
Plains	*(0 <= Relief < 91) and (elev < 100)*

② Fluvial Landform

Category_1	Classification	
1:5,000,000	Threshold number(facc)	Stream Order
Fluvial Landform	Facc >= 1,000,000	1st == Order

**Hydrological modelling and Spatial query**	
Fluvial Landform	*facc >= 1,000,000 → (Select == 1st order)*

③ Coastal Landform

Category_1	Classification
**1:5,000,000**	**Administration Data**
Coastal Landform	Coastal line == Administration Data

**Spatial query**	
Coastal Landform	*Select == Coastline*

④ Islands Landform

Category_1	Classification
**1:5,000,000**	**Administration Data**
Island	Island Polygon == Administration Data

**Spatial query**	
Islands Landform	*Select == Island*

⑤ Baekdudaegan

Category_1	Classification	
**1:5,000,000**	Threshold number(facc)	**History**
Baekdudaegan	(1st == Order) intersect with Watershed	Sangyeongpyo(a mountain chart)) == Daegan

**Hydrological modelling and Spatial query**	
Baekdudaegan	*facc >= 2,000 → (Select == 1st Order) and (intersect with watershed) and (Sangyeongpyo == Daegan)*

⑥ Demilitarized Zone

Category_1	Classification
**1:5,000,000**	**Geopolitics**
DMZ Zone	North and South Truce Line<br>Width of Military Demarcation Line is less 2km

**Headup digitizing: Military Demarcation Line**	
Headup digitizing	*headup digitizing with google earth image*

Category_2

① Mountain and Plains

Category_2	Classification			
**1:1,000,000**	**Relief**	**Elevation/slope**	**Threshold number(facc)**	**Landcover and NDVI**
High Elevation Mt.	250 <= Relief < 1,200	elev >= 1,000	-	-
Middle Elevation Mt.	140 <= Relief < 250	500 <= elev <1,000	-	-
Piedmont	91 <= Relief < 140	100 < elev < 500	-	-
Drainage Divide	-	-	facc >= 100,000 and watershed	-
Water and Lake	-	-	-	Water Body and area >= 100㎢
Wetland in Lake		slope <= 5		Tributary Inflow and – 0.2 < ndvi < 0.3
Alluvial Plains	0 <= Relief < 10	elev <= 30	-	
Undulating Hills	10 <= Relief < 91	30 < elev <= 100	-	

**Map algebra, Hydrological modelling, Spatial query and Headup digitizing**	
High Elevation Mt.	*(250 <= Relief < 1,200) and (Elev >= 1,000)*
Middle Elevation Mt.	*(140 <= Relief < 250) and (500 <= Elev < 1,000)*
Piedmont	*(91 <= Relief < 140) and (100 < Elev < 500)*
Drainage Divide	*facc >= 100,000 → watershed*

Water and Lake	*(landcover == Water Body) and (landcover == lake and area >= 100㎢)*
Wetland in Lake	*(landcover == lake and area >= 100) and (select == endpoint) and (slope <= 5) and (-0.2 < NDVI < 0.3) and headup digitizing*
Alluvial Plains:	*(0 <= Relief < 10) and (elev <= 30)*
Undulating Hills	*(10 <= Relief < 91) and (30 < elev <= 100)*

② Fluvial Landform

Category_2	Classification		
**1:1,000,000**	**Sea direction**	**Earth's Crust**	**Tectonic/Sealevel/geology**
Uplift Coast	East	Continent	Uplift
Rias Coast	West and South	Continent	Sea Level Rise
Volcanic Coast	East and South Sea	Ocean or Continent	Volcanic rock

**Map algebra and Spatial query**	
Uplift Coast	*(direction == East) and (select == Continent) and (select == tectonic)*
Rias Coast	*(direction == west) and (select == Continent) and (select == Sea Level Rise)*
Volcanic Coast	*((direction == east sea) or (direction == south sea)) and ((select == Continent) or (select == Continent)) and (select == Volcanic rock)*

③ Coastal Landform

Category_2	Classification		
**1:1,000,000**	**Sea direction**	**Earth's Crust**	**Tectonic/Sealevel**
Island	West and South	Continent	Sea Level Rise
Volcanic Island	East and South	Ocean or Continent	Volcanic rock

**Map algebra and Spatial query**	
Island	*((direction == west) or (direction == south)) and (select == continent) and (select == Sea Level Rise) and (Select == Island)*
Volcanic Island	*((direction == east) or (direction == south)) and (select == continent) or (elect == Ocean) and (select == Volcanic rock) and (Select == Island)*

Category_2	Classification		
**1:1,000,000**	**Sea direction**	**Earth's Crust**	**Tectonic/Sealevel**
Island	West and South	Continent	Sea Level Rise
Volcanic Island	East and South	Ocean or Continent	Volcanic rock

**Map algebra and Spatial query**	
Island	*((direction == west) or (direction == south)) and (select == continent) and (select == Sea Level Rise) and (Select == Island)*

Volcanic Island	*((direction == east) or (direction == south)) and (select == continent) or (elect == Ocean) and (select == Volcanic rock) and (Select == Island)*

Category_3

① Mountain Landform

Category_3	Classification			
**1: 50,000 ~ 25,000**	**Geology/Tectonic**	**Relief and Elev,Slope,Coast line**	**Threshold number(facc)**	**Landcoer and NDVI**
Mt. Granite	Granite	Condition in Category_2	-	-
Mt.Gneiss Series	Gneiss Series		-	-
Mt.Tertiary Layer	Tertiary Layer		-	-
Mt.Limestone	Limestone		-	-
Mt.Pyroclast	Pyroclast		-	-
Volcanic Mountain	Volcanic Rock	-	-	-
Lava Plateau	Basalt	elev > 800 and Relief <= 300	-	100 < Area < 13,000
High Flat	Except volcanic Rock	Elev > 800 and Relief <= 300	-	5 < Area < 100
Limestone Basin	Limestone	Relief <= 100	-	
Isolated Mountain	-	elev >= 800 including Summit	-	-
Drainage Divide	-	-	facc >= 15.000 and watershed	
Water and Lake	-	-	-	Water Body and 50 <= area <= 100㎢
Wetland in Lake		slope <= 5		Tributary Inflow and -0.2 <NDVI < 0.3
Inland Erosion Basin	Granite with intersect Faultline	Relief <= 100 and Coastline >= 5,000m	-	-
Caldera Basin	Collapsed Crater	Relief <= 10 and elev >= 600	-	-

**Map algebra, Hydrological modelling, Spatial query and Headup digitizing**	
Mt. Granite	*Geology == granite and Mt. in* Category_2
Mt.Gneiss Series	*Geology == Gneiss Series and Mt. in* Category_2
Mt.Tertiary Layer	*Geology == Tertiary Layer and Mt. in* Category_2
Mt.Limestone	*Geology == Limestone and Mt. in* Category_2

Mt.Pyroclast	*Geology == Pyroclast and Mt. in* Category_2
Volcanic Mountain	*Geology == Volcanic Rock and Mt. in* Category_2
Lava Plateau	*Geology == Basalt and (Elev > 800 and Relief <= 300) and (100 < Area < 13,000)*
High Flat	*Geology ne Basalt and (Elev > 800 and Relief <= 300) and (5 < Area < 100)*
Limestone Basin	*Geology == Limestone and Relief <= 100, headup digitizing with Google Image*
Isolated Mountain	*Focalmax(Elev > 800) and elev > 800*
Drainage Divide	*facc >= 15,000 → watershed*
Water and Lake	*(landcover == Water Body) and (landcover == lake and (50 <= area <= 100)*
Wetland in Lake	*(landcover == lake and (50 <= area <= 100) and (select == endpoint) and (slope <= 5) and (-0.2 < NDVI < 0.3), headup digitizing*
Inland Erosion Basin	*Geology == Granite and interslect with faultline and Relief <= 100 and Coastline >= 5,000*
Caldera Basin	*Geology == Volcanic Rock an Relief <= 10 and elev >= 600, headup digitizing with Google Image*

② Plain Landform

Category_3	Classification		
**1: 50,000 ~ 25,000**	**Relief and Elev, slope**	**River mouth Distance form Coastline**	**Landcover and NDVI**
Coastal Plains	Relief <= 91 and elev <= 30	*Coastline < 5,000*	-
Inland Plains	Relief > 91 and 30 <= elev <= 100	*Coastline >= 5,000*	-
Delta Plains	Relief <= 10 and elev <= 5 and Slope < 5	*River mouth and Coastline == 0*	Wetland and Vegetation and Seawater
Water and Lake			Water Body and 10 <= area < 50
Wetland in Lake			Tributary Inflow and -0.2 <NDVI < 0.3
Low Gentle Slope	10 <= relief < 91 and 30 <= elev <= 100	-	-

**Map algebra, Spatial query and Headup digitizing**	
Coastal Plains	*(Relief <= 91 and Elev <= 30) and Coastline < 5,000*
Inland Plains	*(10 < Relief > 91 and 30 < Elev <= 100) and Coastline >= 5,000*
Delta Plains	*(Relief <= 10 and elev <= 5 and Slope < 5) and* Select == 5 or 6st order → intersect == Coastline = 0 → Select == water, wetland, grassland

Water and Lake	*(landcover == Water Body) and (landcover == lake and (10 <= area < 50)*
Wetland in Lake	*(landcover == lake and (10 <= area < 50) and (select == endpoint) and (slope <= 5) and (-0.2 < NDVI < 0.3), headup digitizing*
Low Gentle Slope	*(10 <= relief < 91) and (30 < elev <= 100)*

③ Fluvial Landform

Category_3	Classification			
**1: 50,000 ~ 25,000**	**Relief and Slope, Elev**	**Threshold number(facc)/ Stream Order**	**Geology /Tectonic**	**Category_2/landcover**
Rocky Channel	relief > 91 and Slope > 10	facc >= 15.000 and 1~2st Order	All Bed Rock/Uplift	High Elevation Mt.
Sand and Gravel Channel	10 < relief <= 91 and 10 < elev <= 100 and 5 < Slope <= 10	facc >= 15.000 and 3~4st Order	Granite	Middle Elevation Mt.
Silt and Mud Channel	relief ><= 10 and elev <= 10 and Slope <= 5	facc >= 15.000 and 5~6st Order	All Bed Rock	Alluvial Plains
Water and Lake				Water Body and 10 <= area < 50
Wetland in Lake				Tributary Inflow and –0.2 < NDVI < 0.3

**Map algebra, Hydrological modelling and Spatial query**	
Rocky Channel	*(Relief > 91) and (slope > 10) and (elev > 100) and (facc >= 15,000 → Select == 1~2st Order) and (geology == all Rock and Uplift) and High Elevation Mt.*
Sand and Gravel Channel	*(10 < Relief <= 91) and (5 < slope <= 10) and (10 < elev <= 100) and (facc >= 15,000 → Select == 3-4st Order) and geology == granite and Middle Elevation Mt.*
Silt and Mud Channel	*(Relief <= 10) and (slope <= 5) and (elev <= 10) and (facc >= 15,000 → Select == 5-6st Order) and geology == all BedRock and Alluvial Plains*
Water and Lake	*(landcover == Water Body) and (landcover == lake and (10 <= area < 50)*
Wetland in Lake	*(landcover == lake and (10 <= area < 50) and (select == endpoint) and (slope <= 5) and (-0.2 < NDVI < 0.3), headup digitizing*

④ Coastal Landform

Category_3	Classification				
**1: 50,000 ~ 25,000**	**Sea direction/Headland/Coastype**	**Geology**	**Sea Energe**	**Landcover**	**Tectonic/Sealevel/geology**
Rocky Coast	East/West and Headland	Gneiss, Pyroclast	Wave		Uplift
Sand Coast	East and Bay Beach/ West and headland	Granite	Wave	Sand	Uplift
Sand and Mud Coast	West/Headland Beach	Granite	Wave and Tide		Sealevel rise
Sand and Gravel Coast	East/South and Headland Bay and beach	Granite	Wave		Uplift
Mud Coast	West	Granite, Gneiss	Tide	Tidalflat	Sealevel rise
Mixed Coast	South	Gneiss, Pyroclast	Wave and Tide		Middle

**Map algebra and Spatial query**	
Rocky Coast	*(direction == East or direction == West) and Headland and (Geology == Gneiss or Geology == pyroclast) and Sea engerge == Wave and tectonic == uplift*
Sand Coast	*((direction == East and Coastype == Bay) or (direction == West and Coastype == haedland)) and (Geology == Granite) and Sea engerge == Wave and tectonic == uplift and select = sand*
Sand and Mud Coast	*((direction == West and Coastype == headland beach) and and (Geology == Granite) and (Sea engerge == Wave and Sea engerge == tide) and (select==SeaLevelRise)*
Sand and Gravel Coast	*((direction == East or direction == South) and (Coastype == headland Bay or Coastype == Beach) and (Geology == Granite) and (Sea engerge == Wave) and tectonic == uplift*
Mud Coast	*((direction == West) (Geology == Granite or Gniess)) and (Sea engerge == Tide) and select == mudlfat and (select ==SeaLevelRise)*
Mixed Coast	*((direction==south) and (geology==gneiss or pyroclast) and (Sea engerge == Wave and Sea engerge == tide)*

Category_4

① Mountains

Category_4	Classification							
**Under 1:5,000**	**Relief/Elev**	**Slope TPI**	**Geology / tectonic**	**Area (㎢)**	**Threshold number(facc)/ Stream Order**	**Landcover and NDVI**	**Satellite / SWIR**	**Category_1**
Magma Plateau	Relief <= 300 and elev > 800	-	Basalt	40 < Area < 100	-	Grassland	-	-
Highland Flat	Relief <= 300 and Elev > 800	-	Except volcanic Rock	Area < 5	-	Grassland	-	-
Karst Basin	30 < Relief <= 300	-	Limestone	Area < 5	-	-	Google Headup	-
Karst Flat	Relief <= 30	-	Limestone	Area < 15	-	-	Google Headup	-
Wetland Mt.	Relief <= 30 and Magma Plateau and Highland Flat	-		-	-	Wetland and NDVI < 0	-	-
Wind Hole	Field Survey Data							
Piedmont	90 < Relief < 140 and 100 < elev < 500	-	Granite	Area < 5	-	-	-	-
Ridge	-	10 < S TPI < 700		-	-	-	-	-
Valley	-	-626< S TPI < -120		-	-	-	-	-
Rock Block	elev >= 200 and Slope > 7	10 < S TPI < 700	-	-	-	Barren	0 < NDVI < 0.07 and SWIR > 1.57㎛,, Talus, Google Headup	Mountain
Mountain Cliff	Slope > 40	-626< S TPI < -120	Faultline	-	-	-	-	Mountain

Ridge Saddle	-	-120 < S TPI < 10	-	-	-	-	-	-
Crator	Volcanic Crator							
Mountain Bog	Field Survey Data							
Doline Wetland	Relief <= 20	-	Limestone	-	-	Wetland and NDVI < 0	Google Headup	-
Drainage Divide	-	-		-	Facc >= 3,000	-	-	-
Water and Lake	-	-		-		Water Body and 5 < area < 10	-	-
Wetland in Lake						tributary inflow and -0.2 NDVI 0.3		

**Map algebra, Hydrological modelling, Spatial query and Headup digitizing**	
Magma Plateau	*(Relief <= 300 and elev > 800) and Geology == basalt and (40 < Area < 100) and landcover == grassland*
Highland Flat	*Relief <= 300 and elev > 800) and Geology ne basalt and Area < 5) and landcover == grassland*
Karst Basin	*30 < Relief <= 300 and geology == limestone and Area < 5, Headup digitizing with google*
Karst Flat	*Relief <= 30 and geology == limestone and area < 15, Headup digitizing with google*
Wetland Mt.	*Relief <= 30 and Magma Plateau and Highland Flat and landcover == wetland and NDVI < 0*
Wind Hole	*Field Survey data*
Piedmont	*90 < Relief < 140 and 100 < elev < 500 and geology == granite and Area < 5*
Ridge	*10 < S TPI < 700*
Valley	*-626< S TPI < -120*
Rock Block	*(elev >= 200 and Slope > 7) and (10 < S TPI < 700) and landvoer == barren and 0 < NDVI < 0.07 and SWIR > 1.57㎛ and select == mountians,*<br>*Talus Headup digitizing with google and*
Mountain Cliff	*Slope > 40 and (-626< S TPI < -120) and geology == faultline and select == mountain*
Ridge Saddle	*-120 < S TPI < 10*
Crator	*Geology == Volcanic crator*
Mountain Bog	*Field Survey Data*
Doline Wetland	*Relief <= 20 and Geology == limestone and landcover == Wetland and NDVI < 0*
Drainage Divide	*Facc >= 3,000 → waterbasin*

Water and Lake	*(landcover == Water Body) and (landcover == lake and (5 < area < 10)*
Wetland in Lake	*(landcover == lake and (5 < area < 10) and (select == endpoint) and (slope <= 5) and (-0.2 < NDVI < 0.3), headup digitizing*

② Plains

Category_4	Classification					
**Under 1:5,000**	**Relief/Elev/slope**	**Distance form Coastline**	**Geology**	**Area(㎢)**	**NDVI**	**Landcover**
Bar and Grassland	Relief <= 90 and Elev < 100	-	Granite	-	-	Sand Bar or Grassland
Waterway and Wetland	Relief <= 90 and Elev < 100	-	-	-	NDVI < 0	Canal or Paddy Field Wetland
Small irrigation pond and Canal	Relief <= 90 and Elev < 100	-	-	-	NDVI < 0	Dumbeon or waterway
Small Inland Plains	Relief <= 100 and 30 <= elev <= 100	over 5,000	-	Area<20	-	Paddy Field Wetland
Small Coastal Plains	Relief <= 91 and elev <= 30	under 5,000	-	Area<20	-	Paddy Field Wetland
Inland Wetland	Relief <= 100 and 30 <= elev <= 100	over 5,000	-	-	NDVI < 0	Wetland or Paddy Field Wetland
Isolated Low Hills	10 <= relief < 91 and 30 <= elev <= 100 not ridge	-	-	Area<1	-	Vegetation
Low Relief Gentle Slope	10 <= relief < 91 and 30 <= elev <= 100 and slope < 7	-	Granite	Area<20	-	Dry Field
Paddy Field Wetland	-	-	-	-	NDVI < 0	Paddy Field Wetland
Water and Lake	-	-	-	Area<5	-	Water Body
Wetland in Lake	-	-	-	-	-0.2 < NDVI < 0.3	tributary inflow
**Map algebra, Spatial query and Headup digitizing**						

Bar and Grassland	*(Relief <= 90 and Elev < 100) and geology == granite and (landcover == sandbar or landcover == grassland)*
Waterway and Wetland	*(Relief <= 90 and Elev < 100) and NDVI < 0 and (landcover == canal or landcover == lPaddy Field Wetland)*
Small irrigation pond and Canal	*Relief <= 100 and (1 <= elev <= 100) and NDVI < 0 and (landcover == Dumbeong or landcover == waterway)*
Small Inland Plains	*Relief <= 100 and (30 <= elev <= 100) and coastline > 5,000 and area < 20 and landcover == Paddy Field Wetland*
Small Coastal Plains	*Relief <= 91 and elev <= 30 and coastline < 5,000 and Area < 20 and landcover == Paddy Field Wetland*
Inland Wetland	*Relief <= 100 and (30 <= elev <= 100) and coastline > 5,000 and (landcover == Paddy Field Wetland or landcover == Wetland)*
Isolated Low Hills	*((10 <= relief < 91) and (30 <= elev <= 100) ne ridge) and area < 1 and landcover == vegetatioon*
Low Relief Gentle Slope	*(10 < Relief < 91) and (30 < elev < 100) and slope < 7 and geology == granite and area < 5 and landcover == Dry Field*
Paddy Field Wetland	*landcover == Paddy Field Wetland*
Water and Lake	*Area < 5 and landcover == Water Body and area < 5*
Wetland in Lake	*(landcover == lake and (area < 5) and (select == endpoint) and (slope <= 5) and (-0.2 < NDVI < 0.3), headup digitizing*

③ Fluvial Landform

Category_4	Classification						
**Under 1:5,000**	**Relief/Elev/slope**	**Slope TPI**	**Stream levee Distance/ geology**	**Area /count**	**Threshold number(facc)/ Stream Order**	**Landcover**	**Geololgy / tectonic**
River Terrace	160 <= Relief <= 207 and Elev < 100	0 < S_TPI < 40	out_SDT < 250	-	-	Sand Bar and Grassland	-
Alluvial Island	-	-	Granite	Area>0.1	-	Bar within Steam Channel	-
Riparian Wetland	-	S_TPI < 0	in_SDT < 100	Area>2	-	wetland within Steam Channel	-
Riverside Wetland	Relief <= 10	S_TPI < 0	out_SDT < 100	Area<100	-	External Wetland and Paddy Field Wetland	-
Braided Stream Channel	Relief <= 5	-	Stream channel	Count>10	-	Bar in within Steam Channel	-

Bar	-	-	Stream channel	Count<10		Bar within Steam Channel	-
Riverside Land	Relief <= 5	-	Stream channel	-	-	river levee and Stream channel	-
Stream and Lake	-	-	out_SDT < 200	-	-	water body	-
Wetland in Lake	-	-	-	area<2	tributary Inflow	-	-
Fluvial Cliff	Slope > 40	-626< S TPI < -120	-	-	-	Google headup	Terrace Cliff/Faultline and Incised Stream

**Map algebra, Hydrological modelling and Spatial query**	
River Terrace	*(160 <= Relief <= 207) and Elev < 100 and (160 <= Relief <= 207) and out_SDT < 250 and (landcover == Sand bar or landcover == grassland)*
Alluvial Island	*Geology == granite and Area > 0.1 and (Landcover == Bar within Steam Channel)*
Riparian Wetland	*S_TPI < 0 and in_SDT < 100 and Area > 2 and (landcover == water body or and landcover == barren or landcover == grassland*
Riverside Wetland	*Relief <= 10 and S_TPI < 0 and out_SDT < 100 and Area < 100 and(landvoer == wetland or landcover == paddy field wetland)*
Braided Stream Channel	*Relief <= 5 and select == stream channel and Count > 10 and landcover == bar*
Bar	*select == stream channel and Count < 10 and landcover == bar*
Riverside Land	*Relief <= 5 and select == stream channel and landcover == barren*
Stream and Lake	*out_SDT < 500 and landcover == water body*
Wetland in Lake	*landcover == lake and (area < 5) and (select == endpoint) and (slope <= 5) and (-0.2 < NDVI < 0.3), headup digitizing*
Fluvial Cliff	*Slope > 40 and (-626< S TPI < -120) and geology == faultline and select == incised Stream*

④ Coastal Landform

Category_4	Classification				
**Under 1:5,000**	**Relief/Elev**	**Geology /Tectonic**	**Wave /Tide/wind**	**Coastal Line Distance / landform**	**Landcover/area㎢**
Coastal Terrace	7 <= Relief <= 70 and 10 <= Elev <= 90	Pyroclast and Sedimentary Rock/Uplift	Wave	CLD < 250	area < 2
Rocky Beach	-	Gneiss, Pyroclast/Uplift	Wave	Headland	area < 2
Sand Beach	-	Granite	Wave	East and West/Beach and Headland	Sand beach, area < 2
Sand and Mud Beach	-	Granite	Wave/Tide	West Bay/Beach	Sand Beach, Tidalflat, area < 2

Sand and Gravel Beach	-	Granite	Wave	Headland/ East and South Beach	Sand Beach, area < 2
Tidalflat	-	-	Tide	West	Tidalflat, area < 2
Mixed Coast	-	-	Wave/Tide	South Beach	Tidalflat, area < 2
Sandune Wetland	-	-	Wave	East and West	Saltmarsh, area < 2
Sand Dune	-	-	Wave/wind	East and West/Beach	Sanddune, area < 2
Salt Marsh	-	-	Tide	West Beach	Saltmarsh, area < 2
Lagoon	-	-	Wave	East	Lagoon, area < 2
Coastal Cliff	-	-	Wave	East/West and Headland	Google headup
Headland	-	gneiss, pyroclast,uplift	Wave	East/West convex	area < 2

**Map algebra and Spatial query**	
Coastal Terrace	*7 <= Relief <= 70 and 10 <= Elev <= 90 and (geology == pyroclast or geology == sedimentary rock) and tectonic == uplift and sea energy == wave and CLD < 250 and area < 2*
Rocky Beach	*(geology == Gneiss or geology == Pyroclast) and tectonic == Uplift and sea energy == wave and coastal landform == headland and area < 2*
Sand Beach	*geology == Granite and emergy == wave and (direction == east or (direction == west and coastype == beach and coastal landform == headland beach)) and landcover == sand and area < 2*
Sand and Mud Beach	*((direction == East or direction == South) and (Coastype == headland Bay or Coastype == Beach) and (Geology == Granite) and (Sea engerge == Wave) and tectonic == uplift and area < 2*
Sand and Gravel Beach	*((direction == East or direction == South) and (Coastype == headland Bay or Coastype == Beach) and (Geology == Granite) and (Sea engerge == Wave) and tectonic == uplift and area < 2*
Tidalflat	*((direction == West) (Geology == Granite or Gniess)) and (Sea engerge == Tide) and select == mudlfat and (select==SeaLevelRise)and area < 2*
Mixed Coast	*((direction==south)and(geology==gneiss or pyroclast)and(Sea engerge == Wave and Sea egerge == tide) and area < 2*
Sandune Wetland	*sea energy == wave and (direction == east or direction == west) and landcover == saltmarsh and area < 2*
Sand Dune	*(sea energy == wave and strong wind) and (direction == east or direction == west) and landcover == sanddune and area < 2*
Salt Marsh	*direction == west and sea energy == tide and landcover == saltmarsh and area < 2*
Lagoon	*sea energy == wave and direction == east and landcover == Lagoon and area < 2*
Coastal Cliff	*sea energy == wave and ((direction == east or west) and headland), headup digitizing with google*
Headland	*tectonic == uplift and sea energy == wave and coastline == east or west convex and geology ==gneiss or geology == pyroclast) and area < 2*

⑤ Islands

Category_4	Classification				
**Under 1:5,000**	**Relief/Elev**	**Coast shape/area**	**Geology /Tectonic/slope**	**Wave and Tide**	**Landcover**
Mud Beach		Small Bay, area < 2	Granite Pyroclast	Tide	TidalFlat
Salt Marsh		Backward Sanddune, area < 1	Granite Pyroclast	Wave and Tide	Saltmarsh
Sand Beach		Small bay, area < 1	Granite Pyroclast	Wave	Sand beach
Sanddune		Sand beach, area < 1	Granite Pyroclast	Wave	SandDune
Lagoon		Backward Sanddune, area < 1	Granite Pyroclast	Wave	Lagoon
Tidal Flat		Small Bay, area < 1	Granite Pyroclast	Tide	Tidalflat
Cliff		Headland, area < 1	Faultline/slope	Wave	-
Coastal Terrace	0 <= Relief <= 70 and 1 <= Elev <= 50	area < 1			

**Map algebra and Spatial query**	
Mud Beach	*coastline == concave and area < 2 and (geology == granite or geology == gneiss or geology == pyroclast) and sea energy = tide and landcover == tidalflat*
Salt Marsh	*area < 1 and (geology == granite or geology == gneiss or geology == pyroclast) and (sea energy = tide or sea energy == wave) and landcover == saltmarsh*
Sand Beach	*coastline == concave and area < 1 and (geology == granite or geology == pyroclast) and sea energy = wave and landcover == sand beach*
Sanddune	*coastline == concave and area < 1 and geology == granite and sea energy == wave and landcover == sanddune*
Lagoon	*sea energy == wave and area < 1 and (geology == granite or geology == pyroclast) and sea energy == wave and landcover = water and lagoon*
Tidal Flat	*coastline == concave and area < 1 and ea energy == tide and landcover == tidalflat*
Cliff	*coastline == convex and geology == faultline and slope > 40 and sea energy == wave*
Coastal Terrace	*0 <= Relief <= 70 and 5 <= Elev <= 50 and (geology == pyroclast or geology == sedimentary rock) and tectonic == uplift and sea energy == wave and area < 2*

⑥ Baekdudaegan Ecozone

Category_4	Classification	
**Under 1:5,000**	**Geology**	**Elevation**
Baekdudaegan Ecozone	7 Geological Zone base on 6 Bedrock(Granite, Gneiss series, Limestone, Basalt)	elev >= 350

**Map algebra and Spatial query**	
Baekdudaegan 1,2,3 Ecozone	*geology == granite and elev >= 350 → 1,2,3 ecozone*

Baekdudaegan 4,5 Ecozone	*geology == limestone and elev >= 350 → 4,5 ecozone*
Baekdudaegan 6 Ecozone	*geology == gneiss series and elev >= 350 → 6 ecozone*
Baekdudaegan 7 Ecozone	*geology == basalt and elev >= 350 → 7 ecozone*

⑦ DMZ Ecozone

Category_4	Classification
**Under 1:5,000**	**Flow Accumulation**
DMZ Ecozone	Facc > 2,000 and Basins
**Map algebra and Spatial query**	
DMZ Ecozone 1	Facc > 2,000 → flow accumulation == watershed 1
DMZ Ecozone 2	Facc > 2,000 → flow accumulation == watershed 2
DMZ Ecozone 3	Facc > 2,000 → flow accumulation == watershed 3
DMZ Ecozone 4	Facc > 2,000 → flow accumulation == watershed 4
DMZ Ecozone 5	Facc > 2,000 → flow accumulation == watershed 5
DMZ Ecozone 6	Facc > 2,000 → flow accumulation == watershed 6
DMZ Ecozone 7	Facc > 2,000 → flow accumulation == watershed 7

⑧ Biodiversity and Geodiversity, Weathering Tendency in Korea Peninsular

Category	Criteria	
Biodiversity and Geodiversity	Data	Biodiversity: 160 Thousands Biota in Korea Peninsular → Shannon’s index<br>Geodiversity: Elevation, Slope, Relief, Curvature, TPI, Landform classifications → Shannon’s index → Geodiversity
	Correlation	Total Cor. 0.39 with Geodiversity<br>Over 0.039 of Biodiversty Cor. 0.93 with Geodiversity
Weathering	Data	Climate: Bioclim data, Warm’s index, Coldindex, Continentality,<br>Elevation: DEM, TWI, Solar radiation<br>Geology: Weathering Difference each Bedrock
	Weathering Tendency	**Shallow mechanical Weathering**<br>([temper] <= 9) and ([precipt00] <= 1000) and ([dem] >= 600)
		**Deep mechanical deep Weathering**<br>([temper] <= 9) and ([Precipt00] >= 1000) and ([Precipt00] <= 2200) and ([dem] >= 600) and (([Geol] = 2) or ([Geol] = 4) or ([Geol] = 11) or ([Geol] = 12) or ([Geol] = 17) or ([Geol] = 28) or ([Geol] = 27) or ([Geol] = 32))
		**Mechanical W > Chemical Weathering**<br>([temper] <= 9) and ([precipt00] <= 1000) and ([dem] >= 600)
		**Chemical W > Mechanical Weathering**<br>(([temper] >= 11) and ([temper] <= 32)) and (([precipt00] >= 1110) and ([precipt00] <= 2200))
		**Deep Chemical Weathering**<br>((([temper] >= 9) and ([temper] <= 32)) and (([precipt00] >= 1110) and ([precipt00] <= 2200)) and (([Geol] = 2) or ([Geol] = 4) or ([Geol] = 11) or ([Geol] = 12) or ([Geol] = 17) or ([Geol] = 28) or ([Geol] = 27) or ([Geol] = 32))) and ([Wi100] >= 87)

Supplementary data 3. Landform classification result maps

Category_1

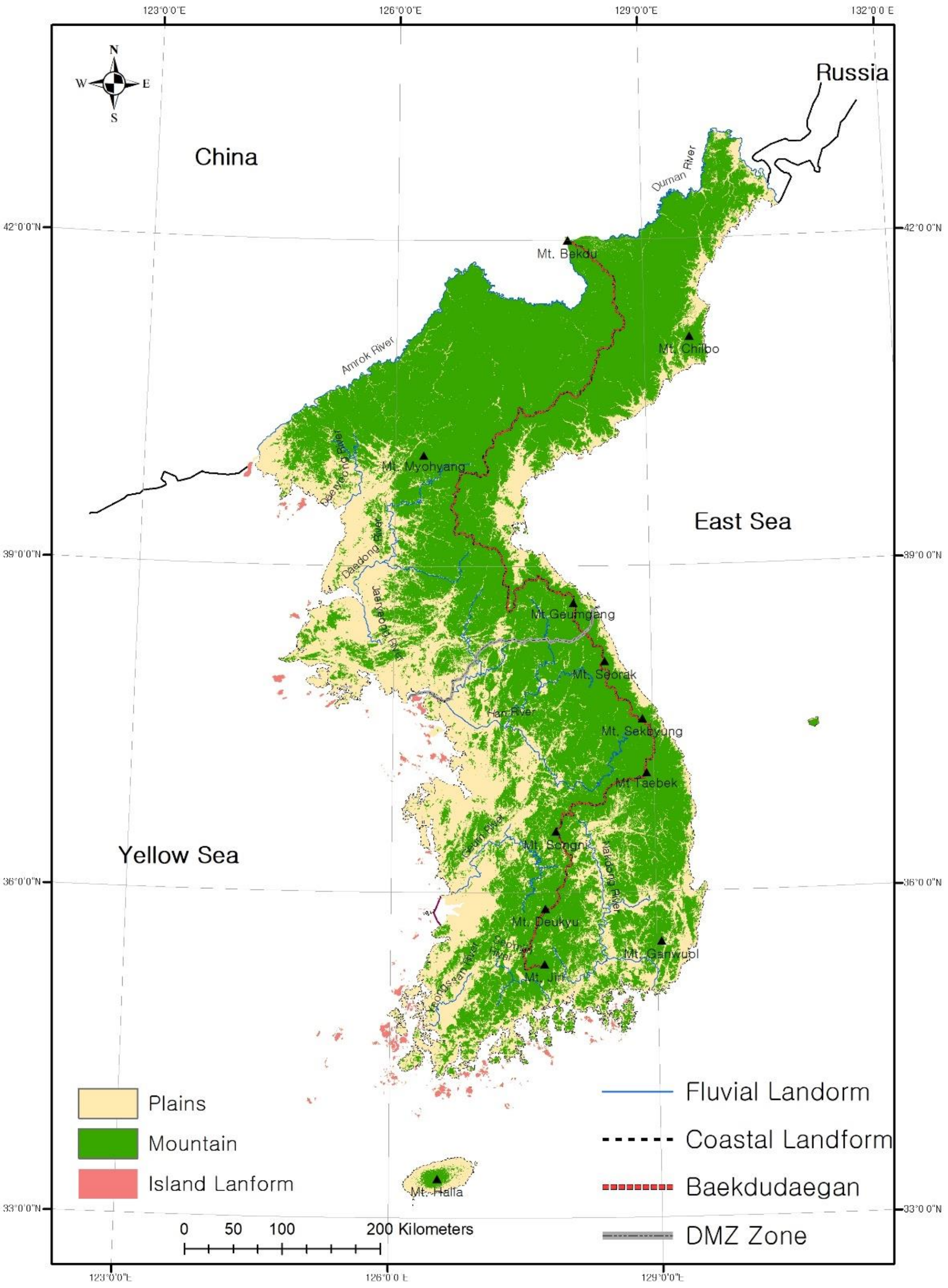

Category_2

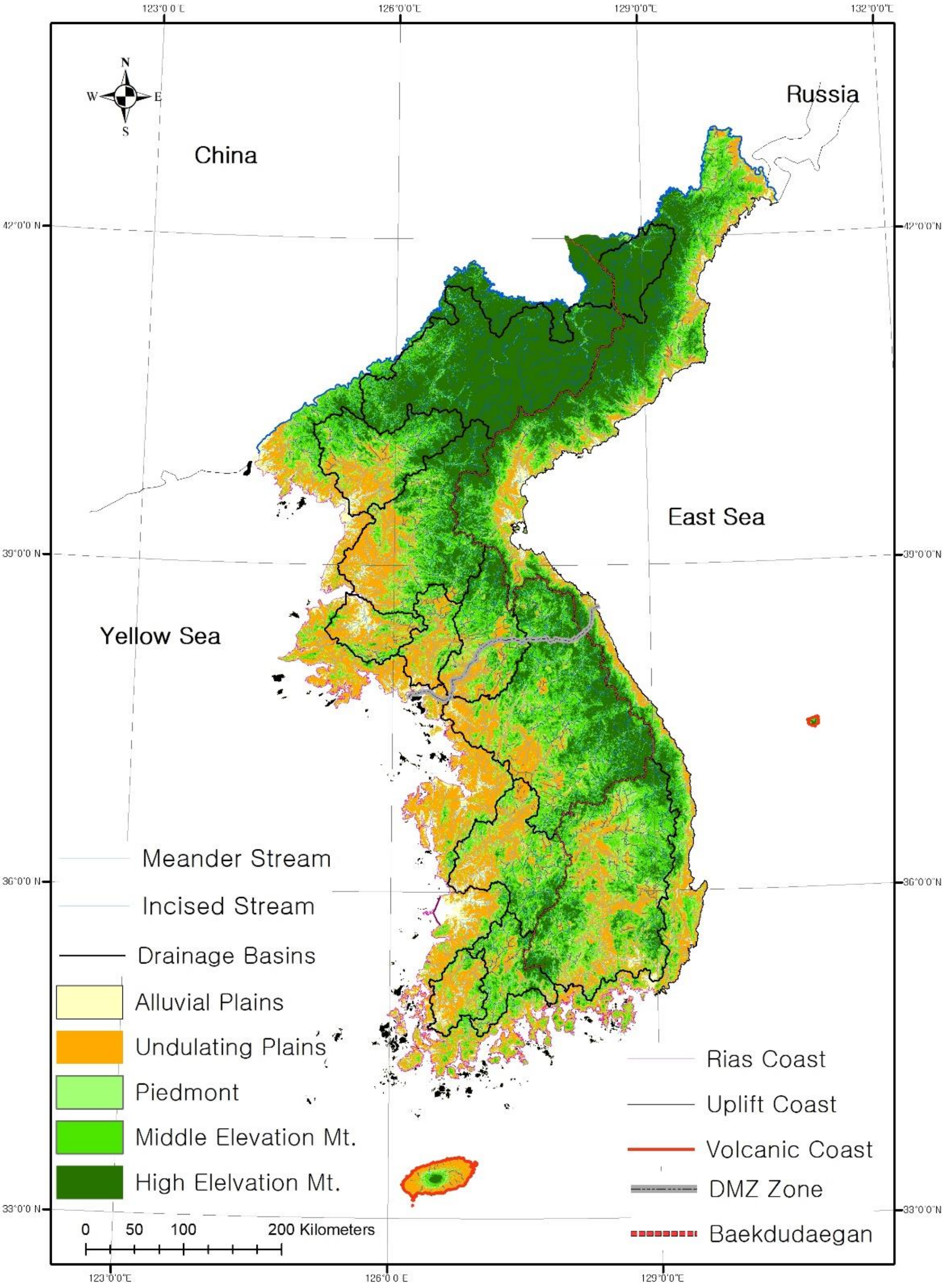

China
Russia
East Sea
Yellow Sea
Meander Stream
Incised Stream
Drainage Basins
Alluvial Plains
Undulating Plains
Piedmont
Middle Elevation Mt.
High Elelvation Mt.
Rias Coast
Uplift Coast
Volcanic Coast
DMZ Zone
Baekdudaegan
0 50 100 200 Kilometers

Elevation

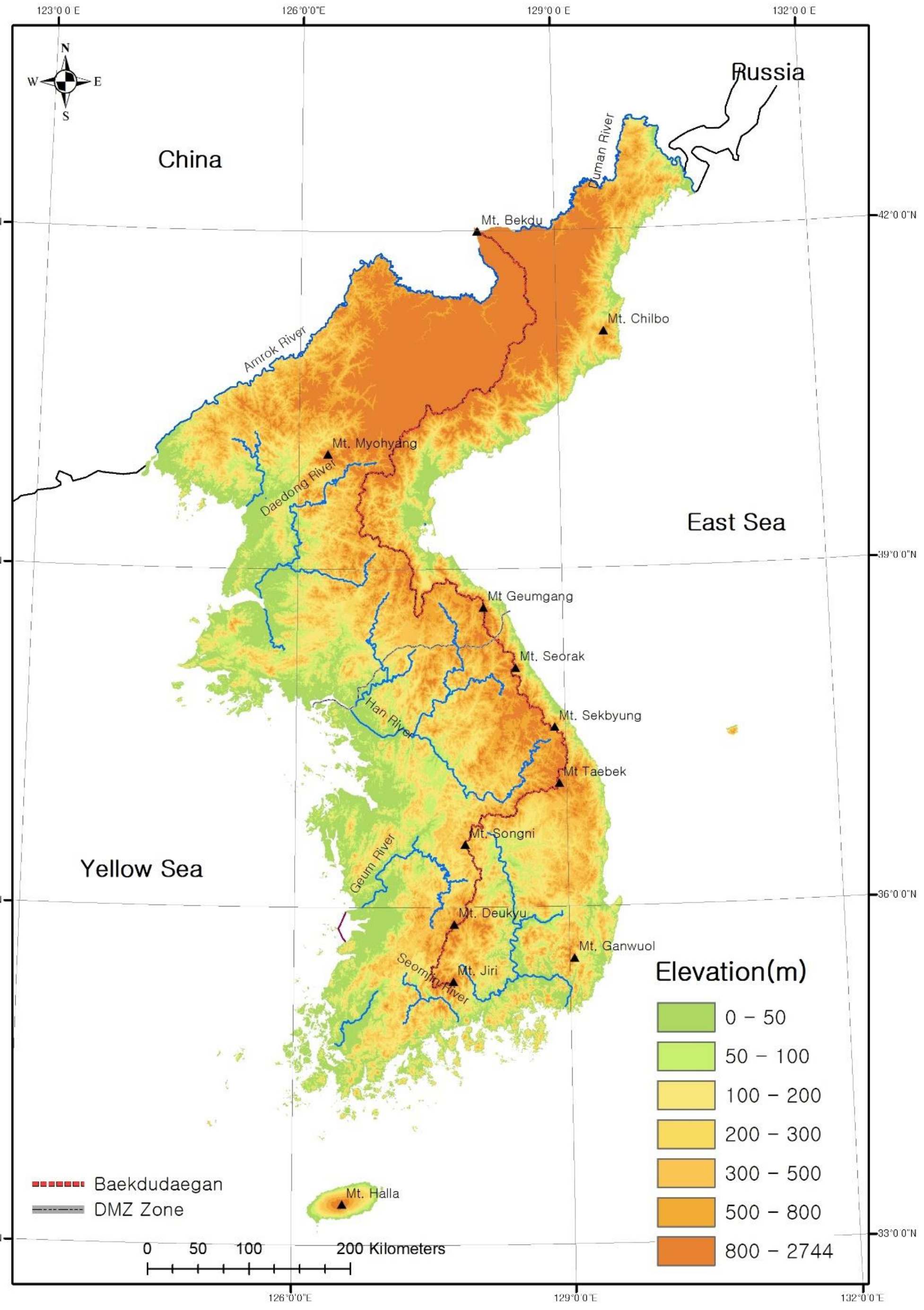

China
Russia
East Sea
Yellow Sea
Duman River
Amrok River
Daedong River
Han River
Geum River
Seomjin River
Mt. Bekdu
Mt. Chilbo
Mt. Myohyang
Mt Geumgang
Mt. Seorak
Mt. Sekbyung
Mt Taebek
Mt. Songni
Mt. Deukyu
Mt. Ganwuol
Mt. Jiri
Mt. Halla
Elevation(m)
0 – 50
50 – 100
100 – 200
200 – 300
300 – 500
500 – 800
800 – 2744
Baekdudaegan
DMZ Zone
0 50 100 200 Kilometers

Weathering

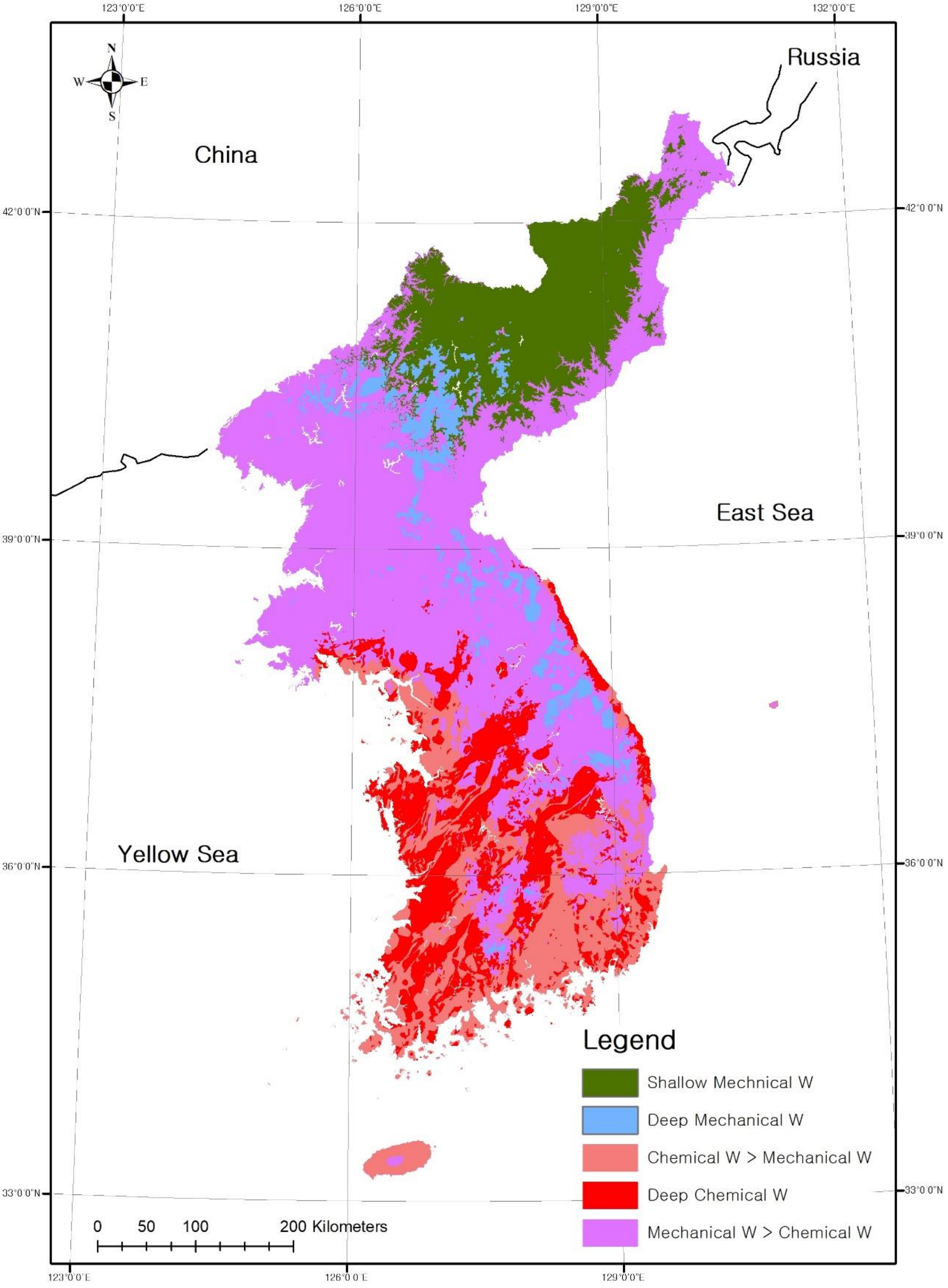

123°0'0"E
126°0'0"E
129°0'0"E
132°0'0"E
42°0'0"N
39°0'0"N
36°0'0"N
33°0'0"N
N
S
W
E
China
Russia
East Sea
Yellow Sea
Legend
Shallow Mechnical W
Deep Mechanical W
Chemical W > Mechanical W
Deep Chemical W
Mechanical W > Chemical W
0 50 100 200 Kilometers

Biodiversity

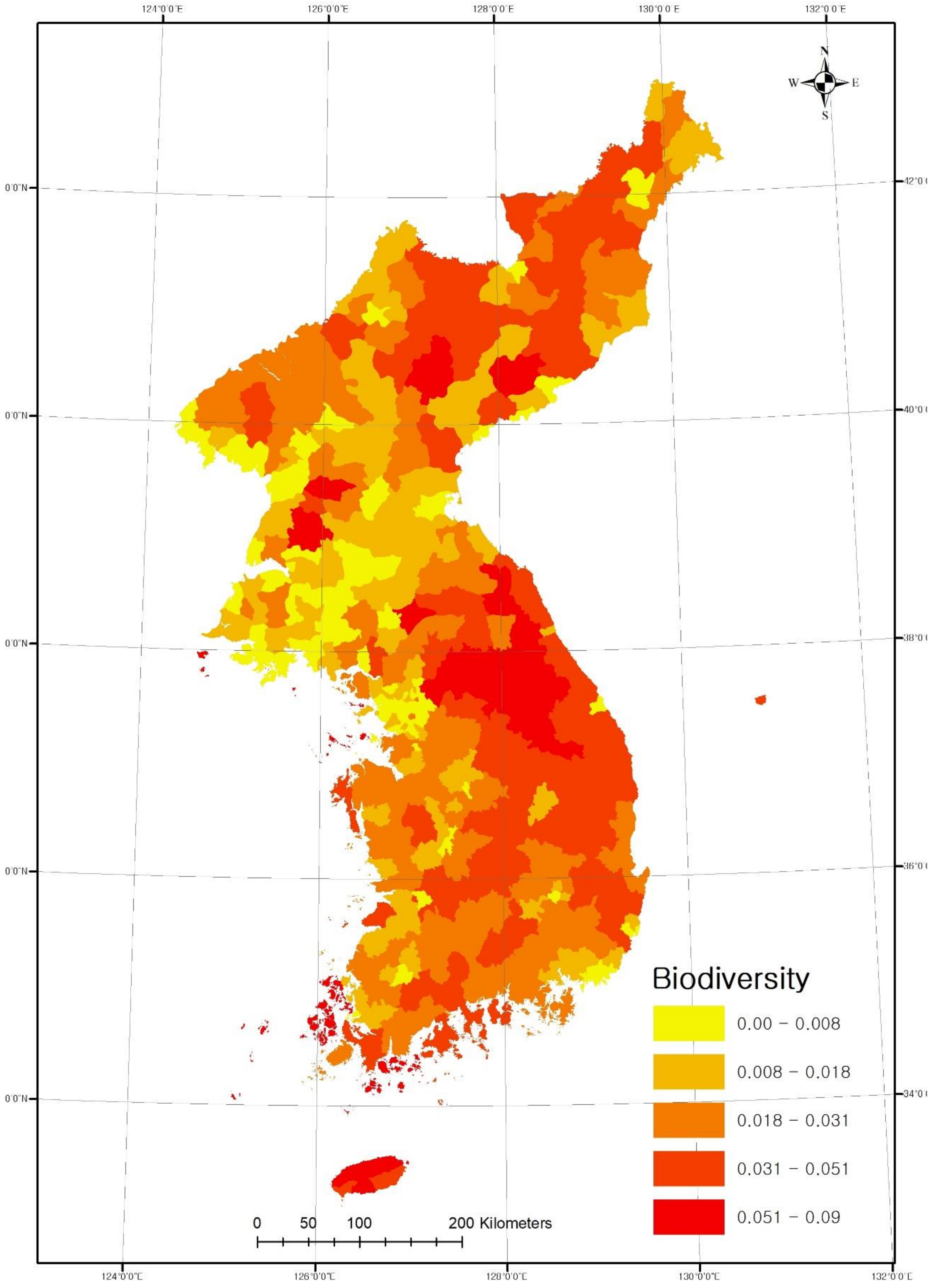

Geodiversity

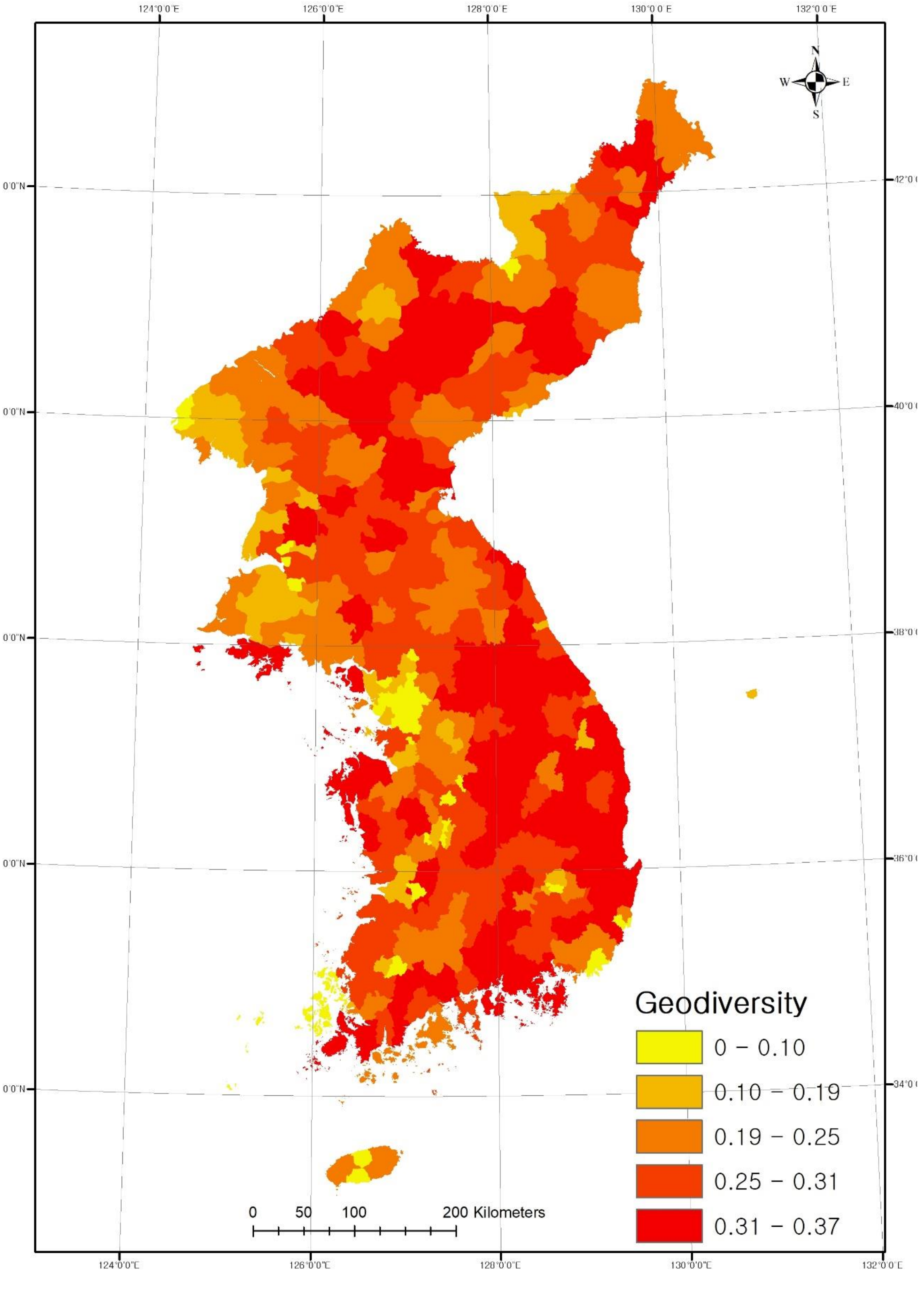

Index area

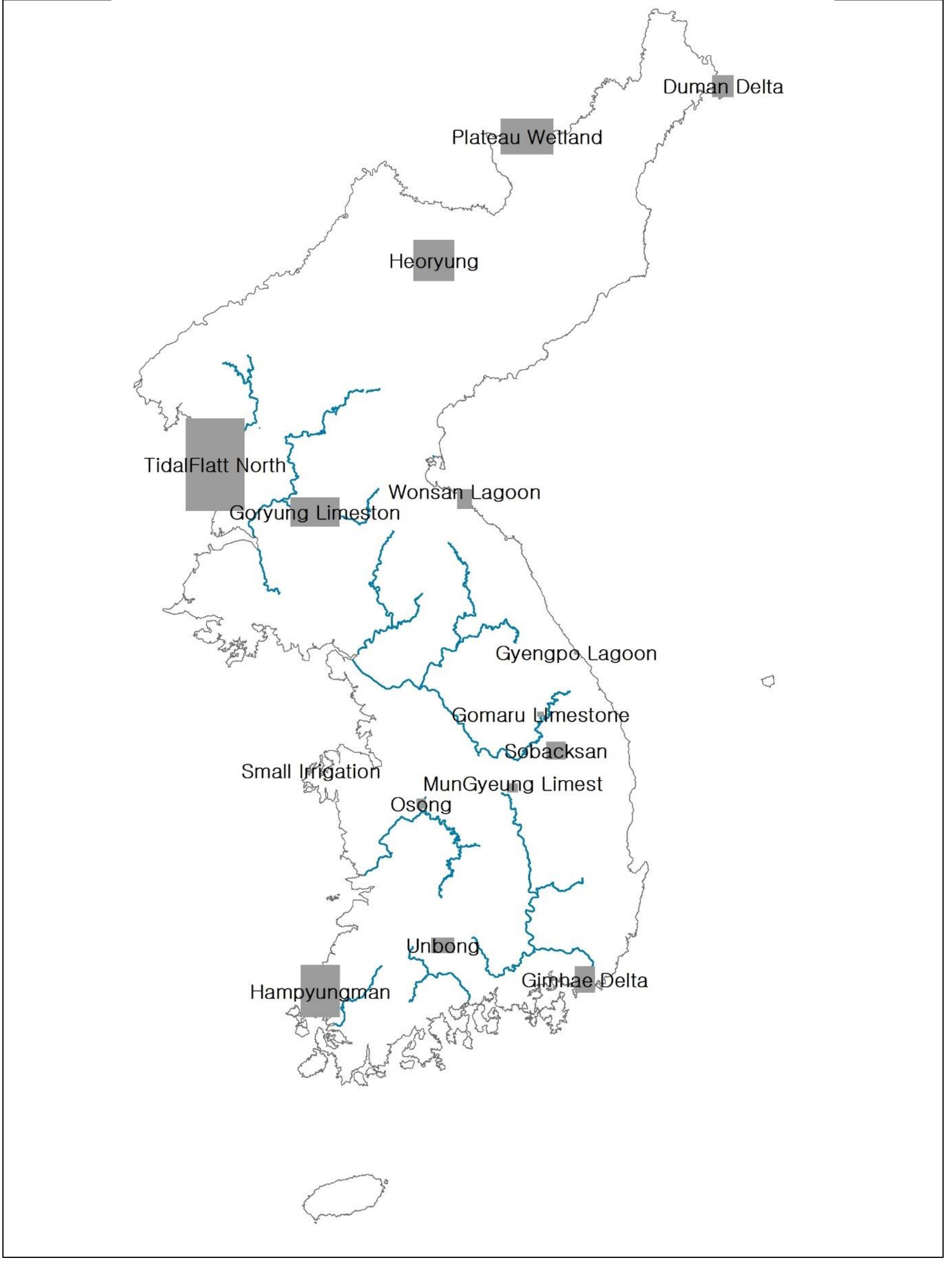
Duman Delta
Plateau Wetland
Heoryung
TidalFlatt North
Wonsan Lagoon
Goryung Limeston
Gyengpo Lagoon
Gomaru Limestone
Sobacksan
Small Irrigation
MunGyeung Limest
Osong
Unbong
Gimhae Delta
Hampyungman

Category_3: Mt. Seolak

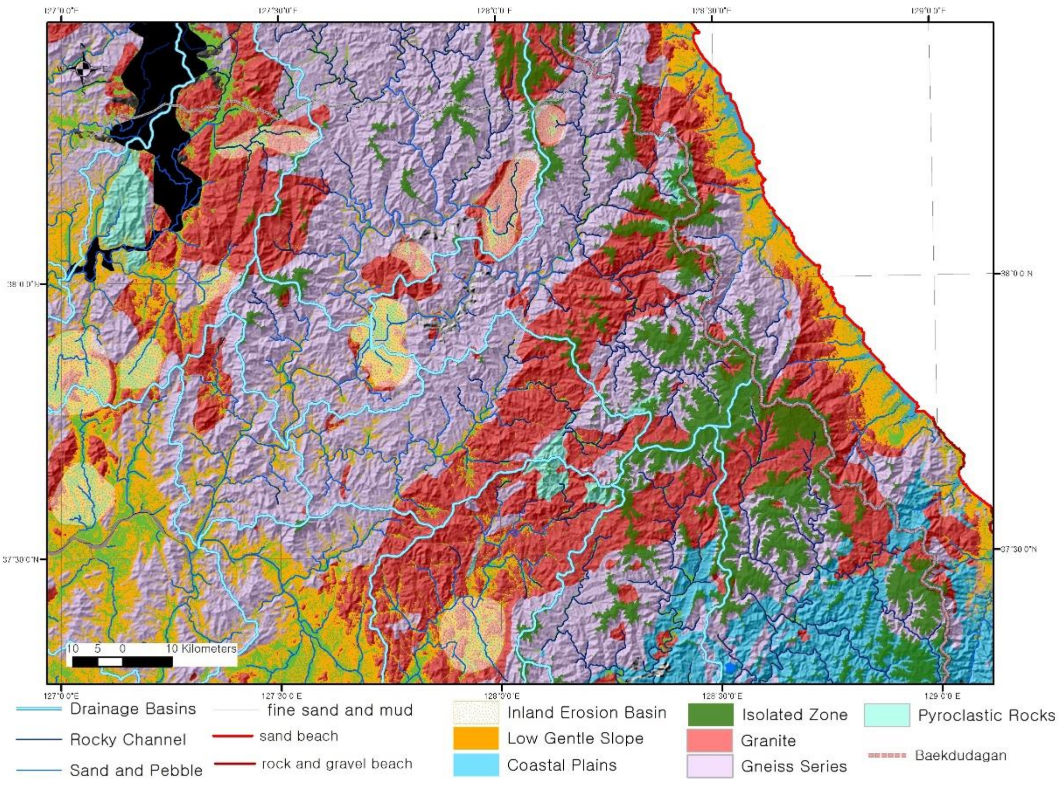

Category_3: Gaema Platuae and Mt. Baekdu

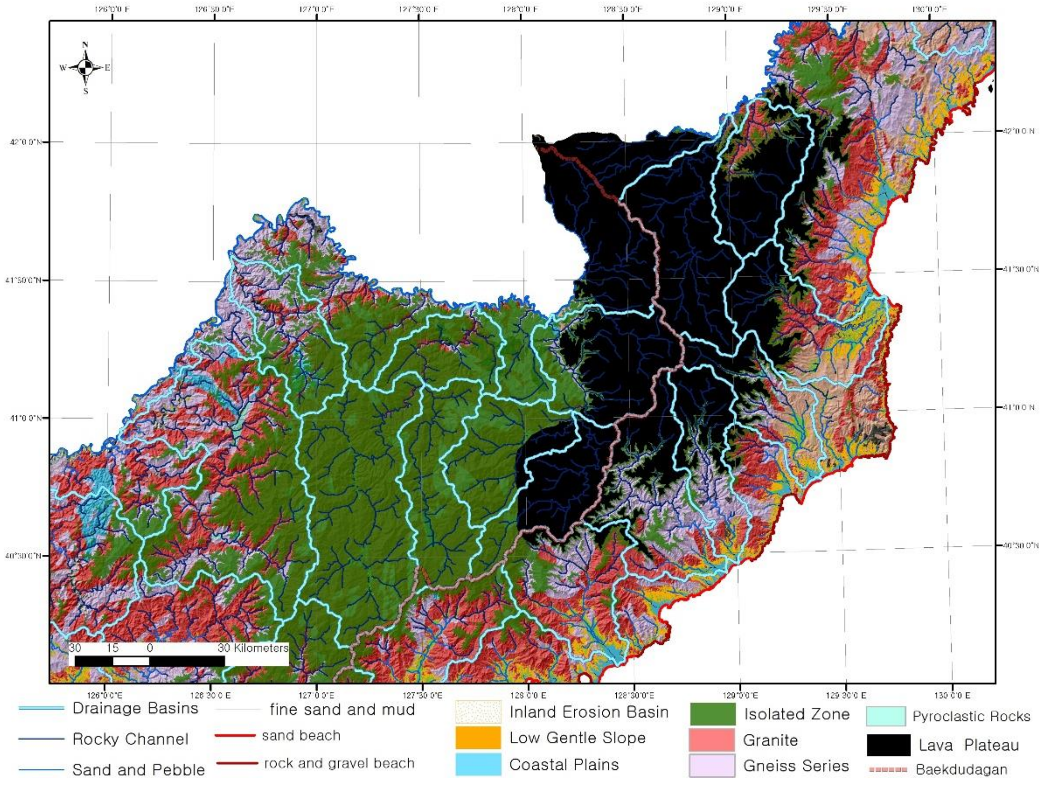

Category_3: Coastal plains, Hills, Basin

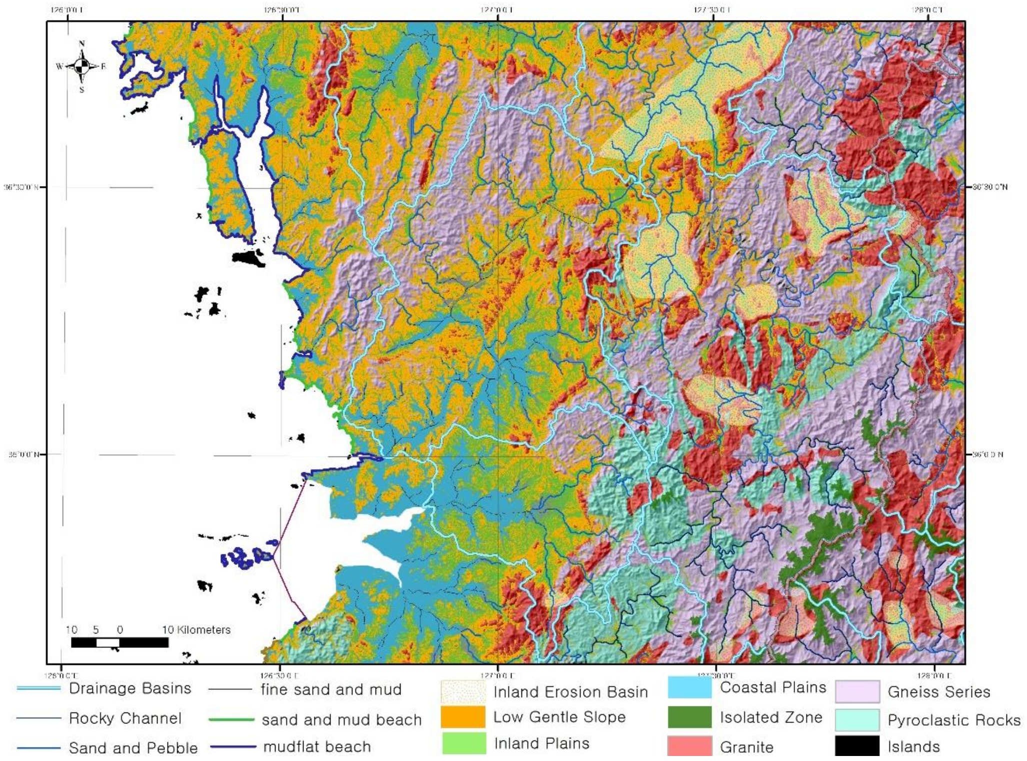

Category_3: Middle of Nakdong River

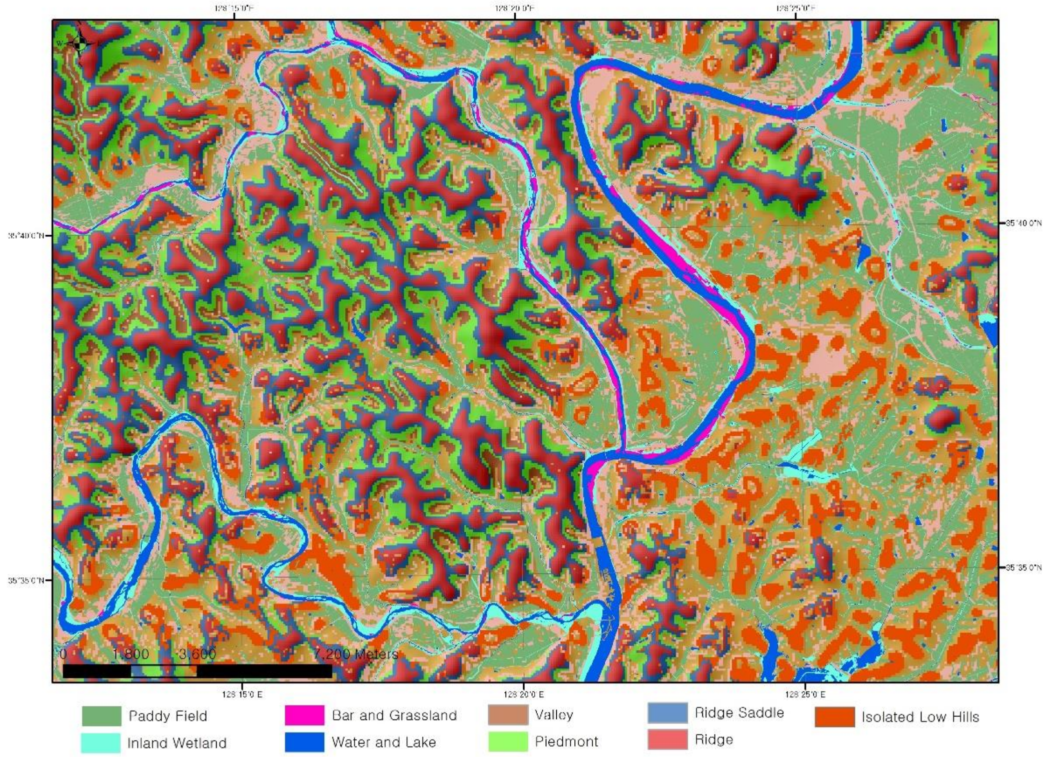

Category_3: Basin Unbong

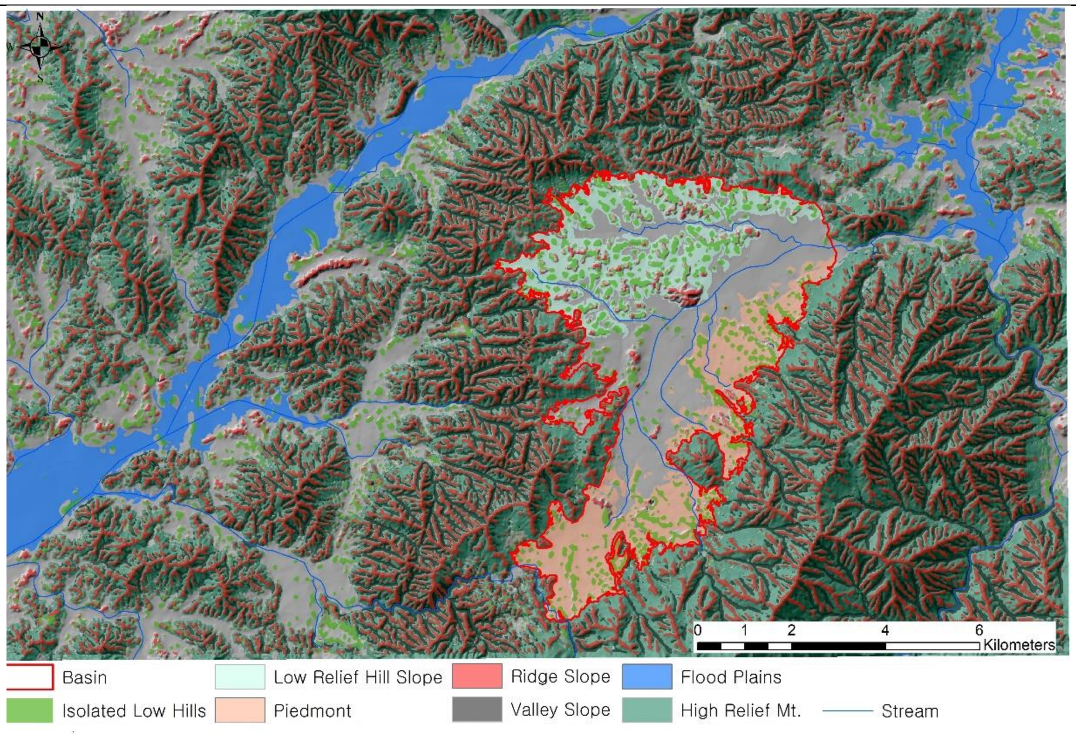

Category_3: Tidal flat, Hampyung

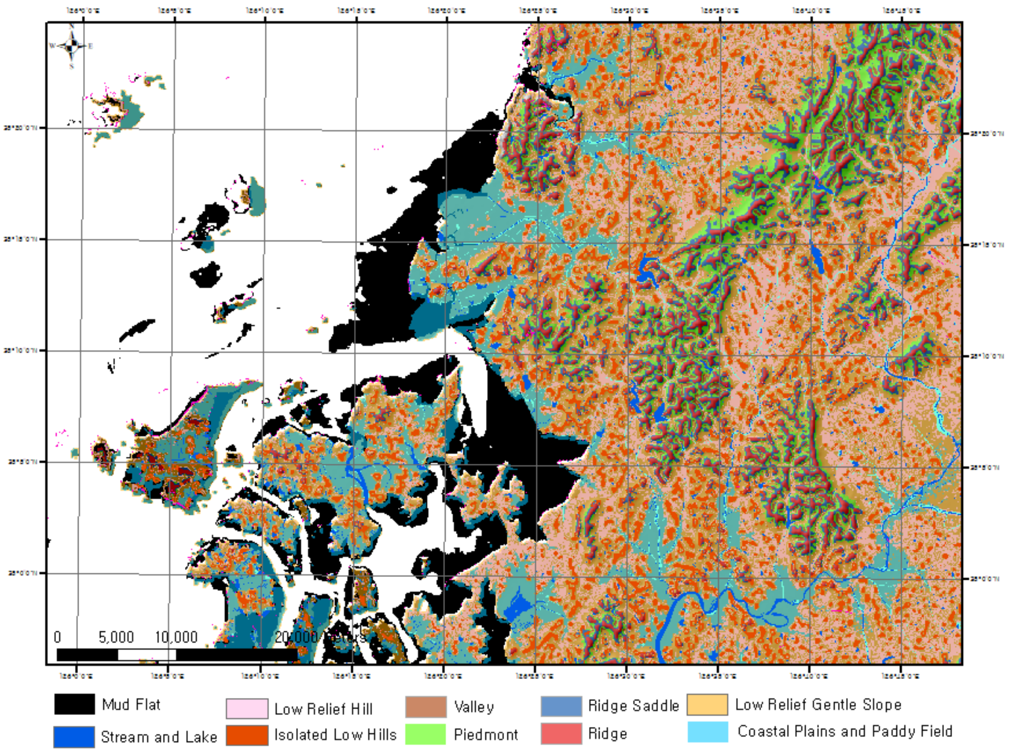

Category_3: Tidal flat, Pyungyang
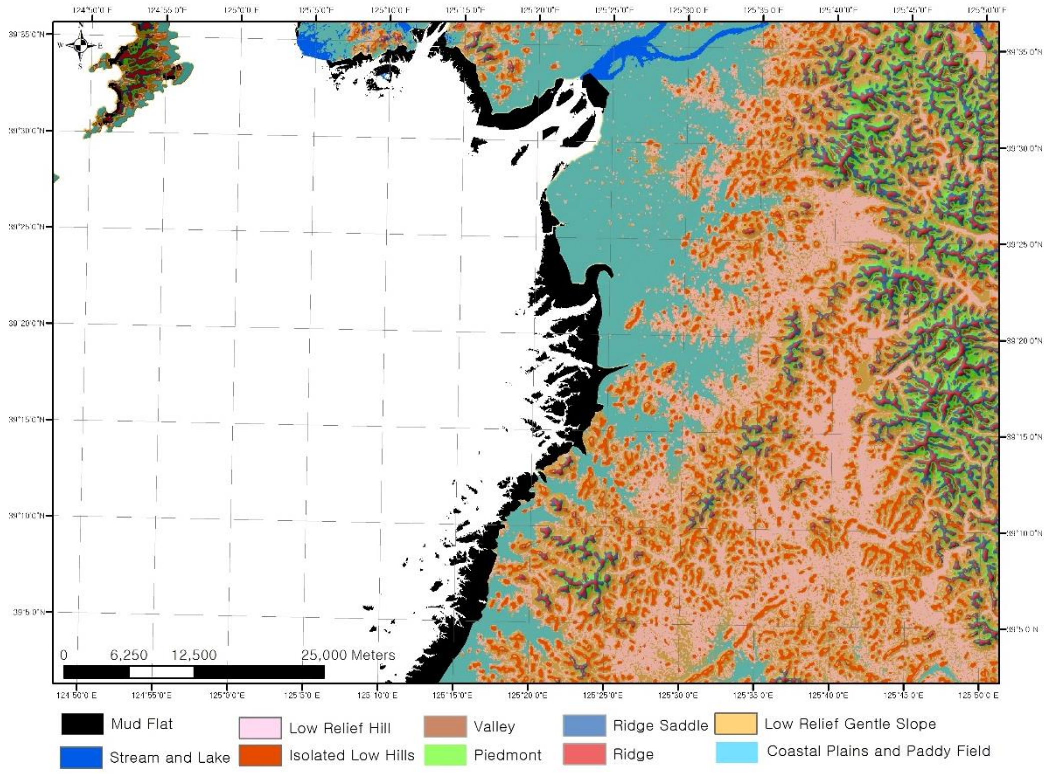
**Category_4: Coastal Terrace, Jeongdongjin**
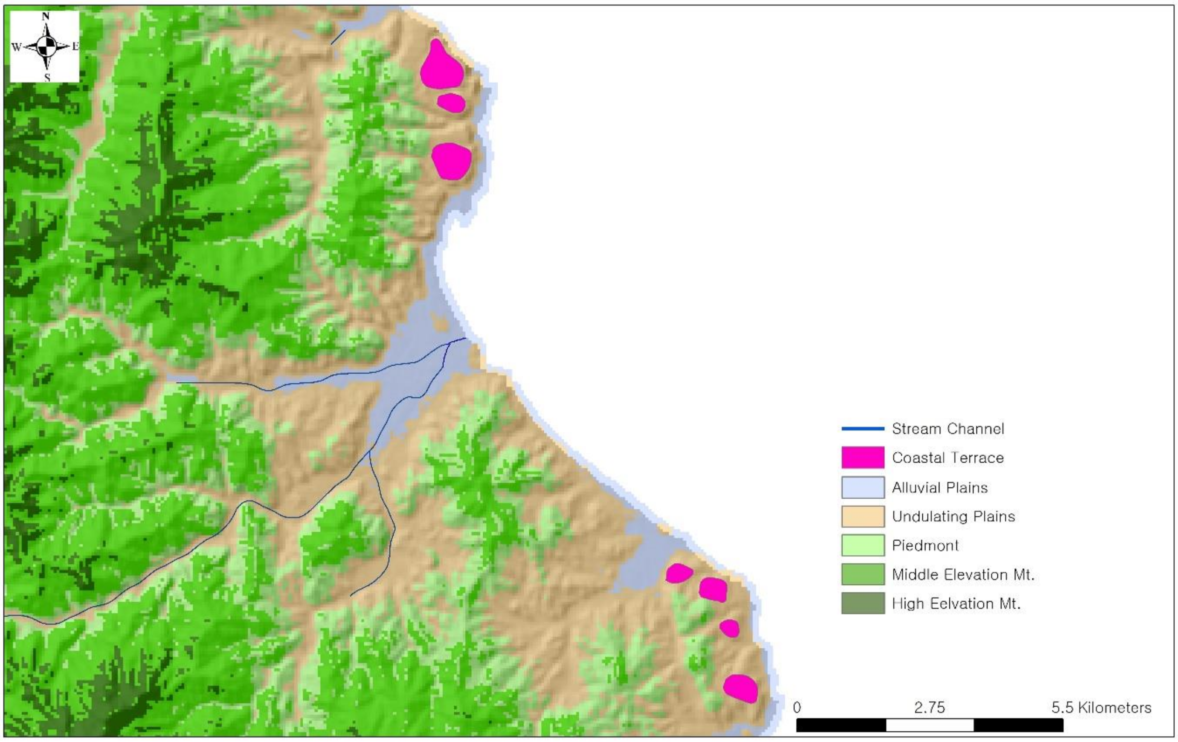

Category_4: Fluvial terrace

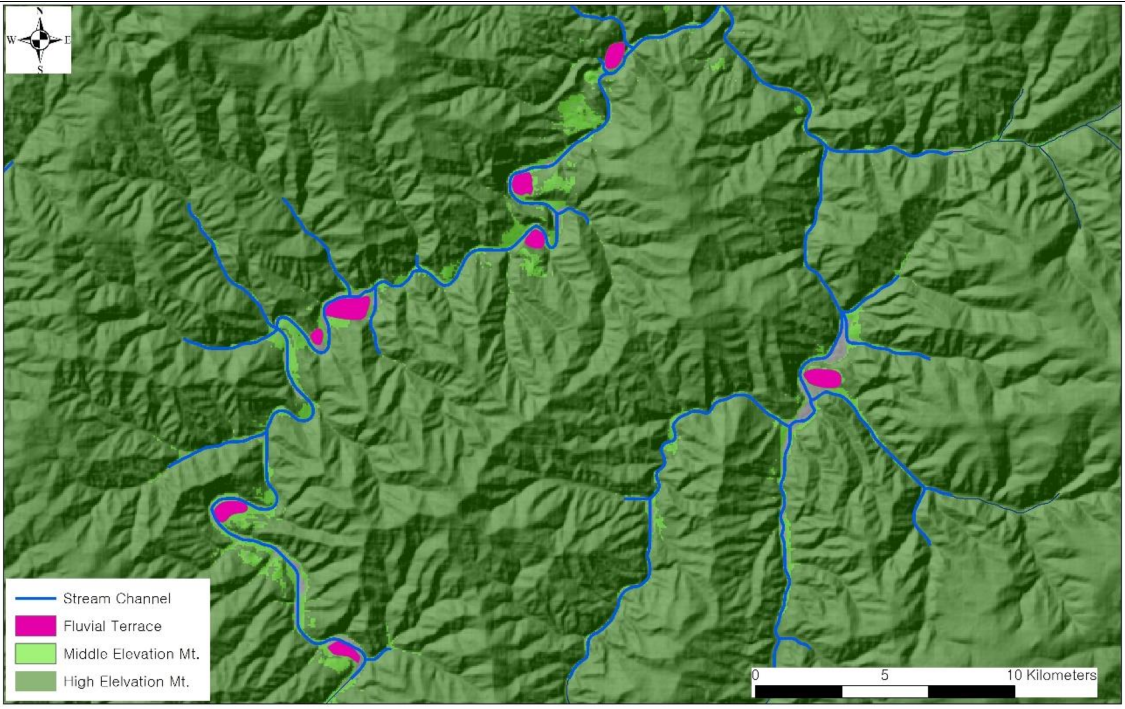

Category_4: Doline and Wetland

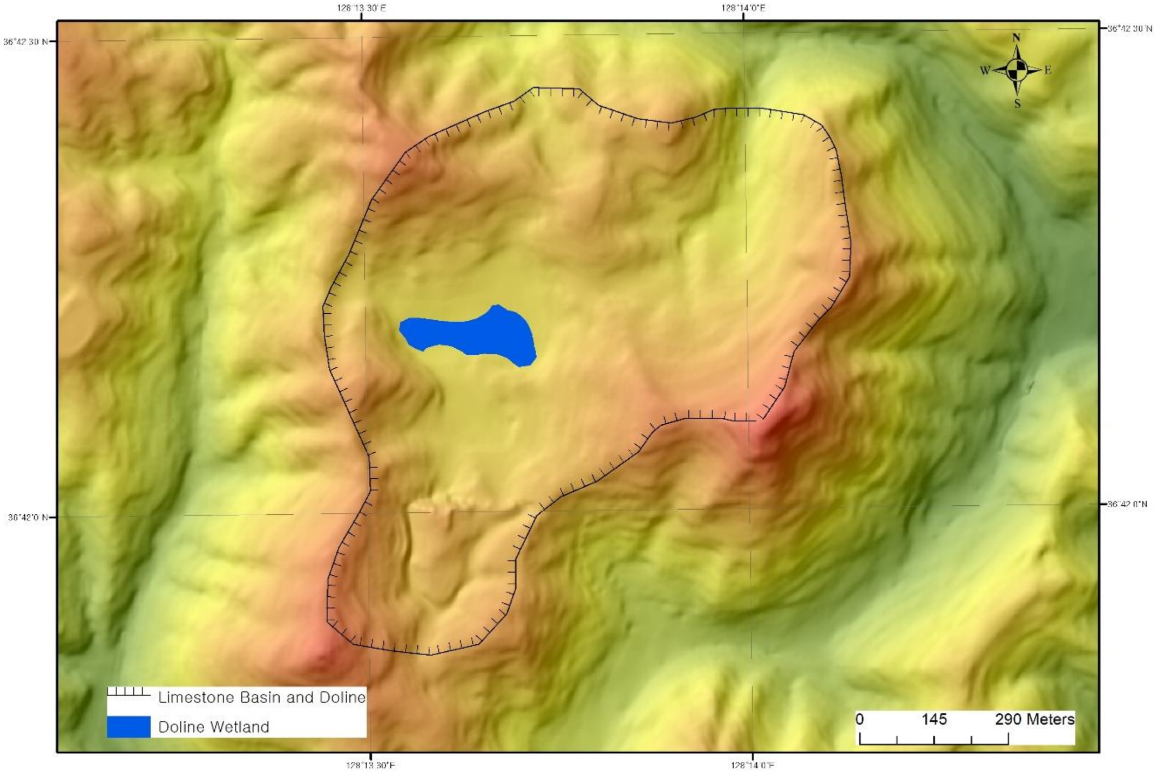

Category_4: Doline and Basin

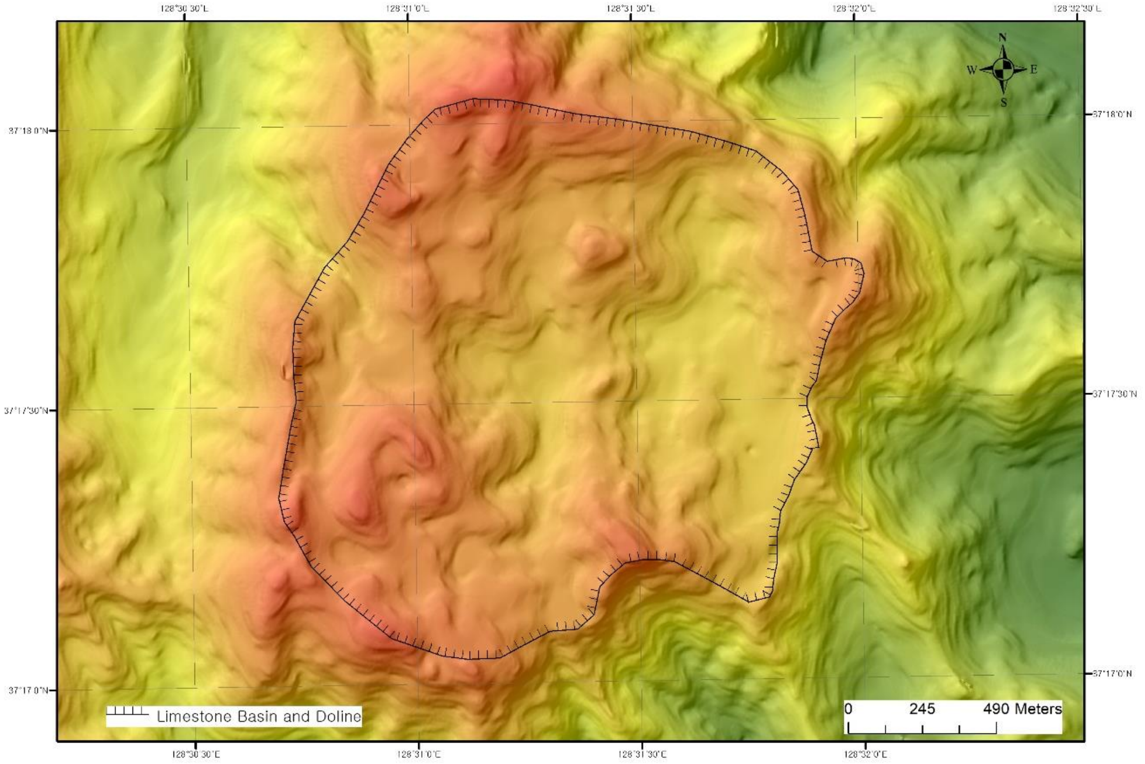

Category_4: limestone Highland, Basin and Dolline

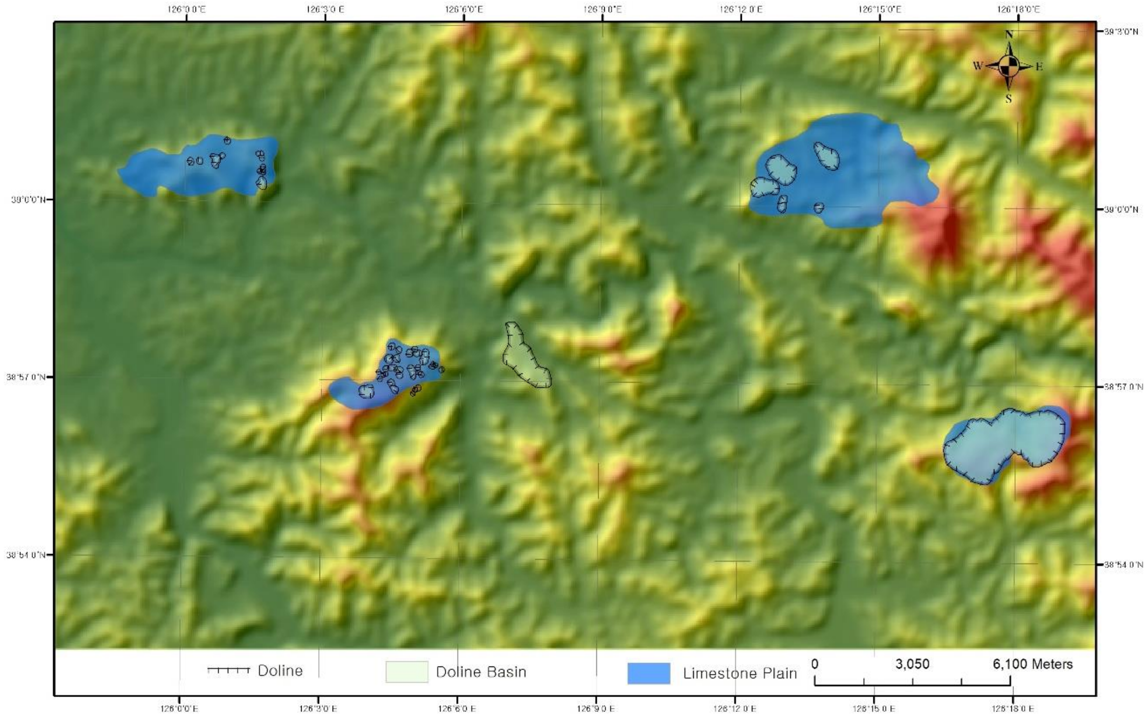

Category_4: Sand bar, Wetlands

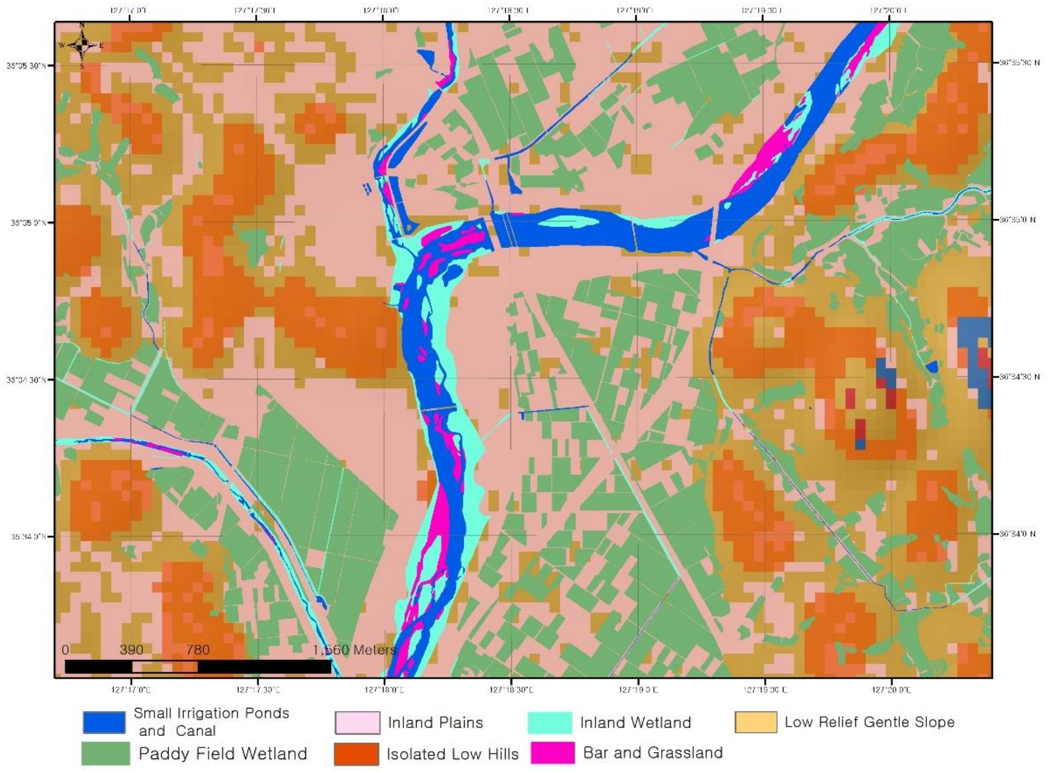

Category_4: Irrigation ponds, Channel, Paddy field wetland

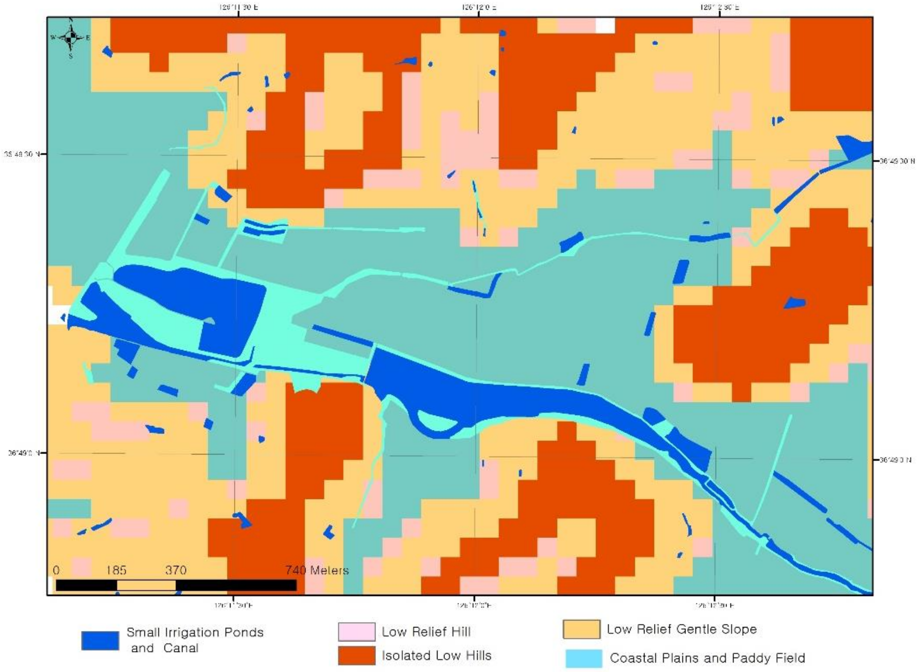

Category_4: sandbar, wetlands

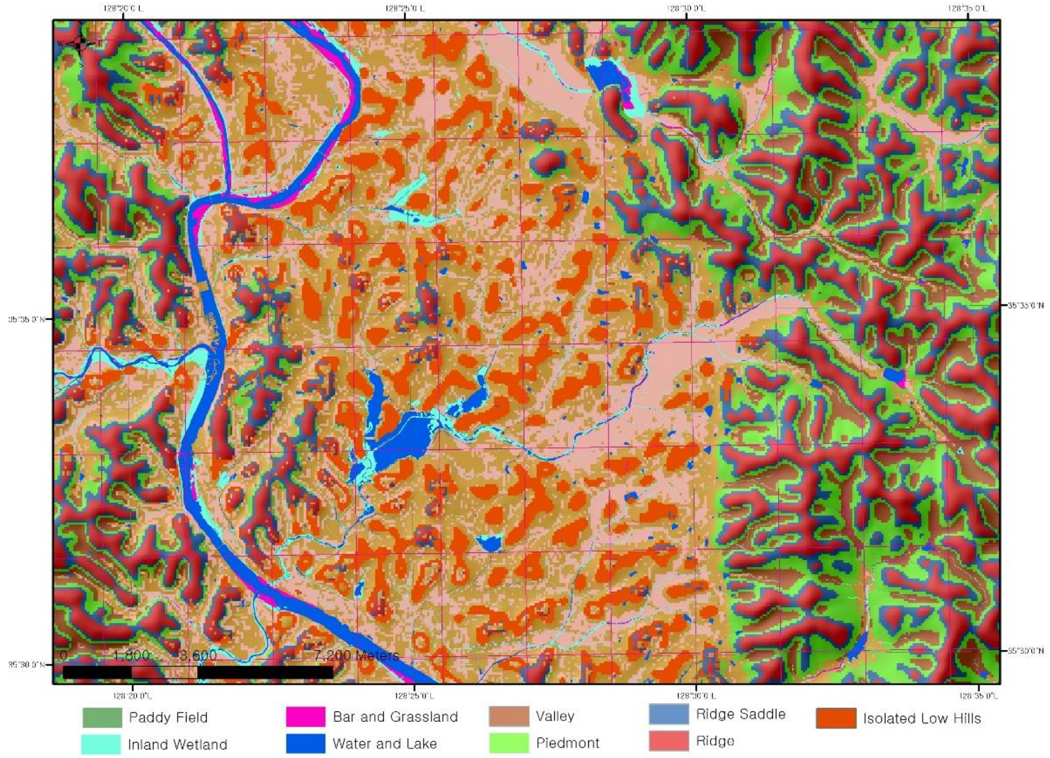

Category_4: Lagoon, Sanddune, Sand beach

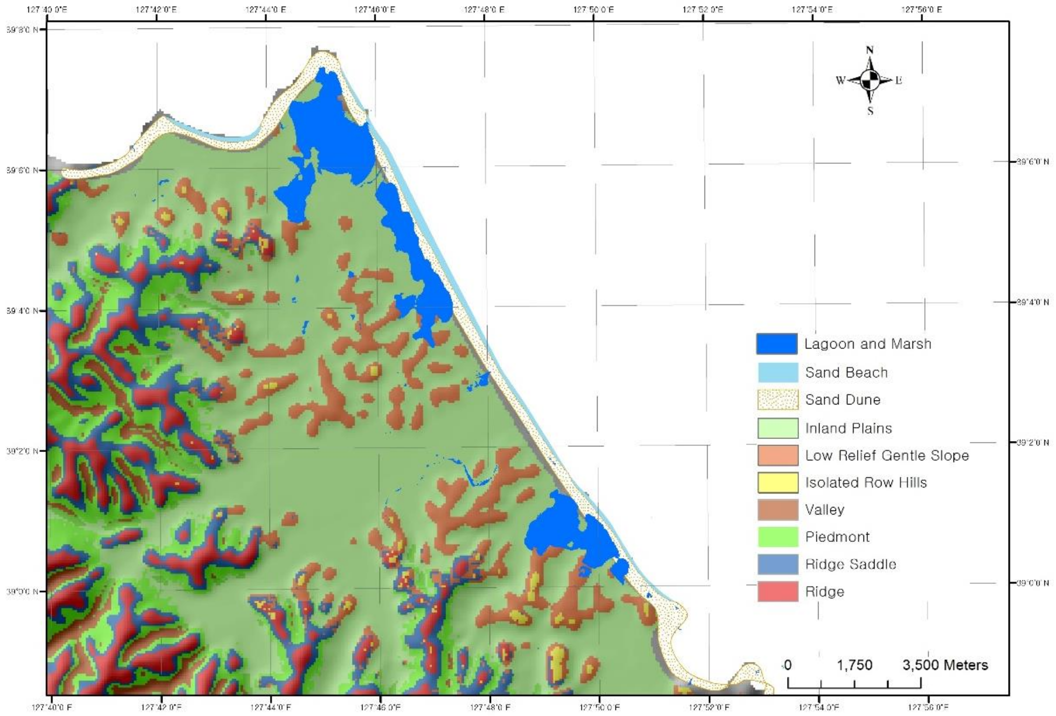

Category_4: Mountain wetlands, Gaema Plateau

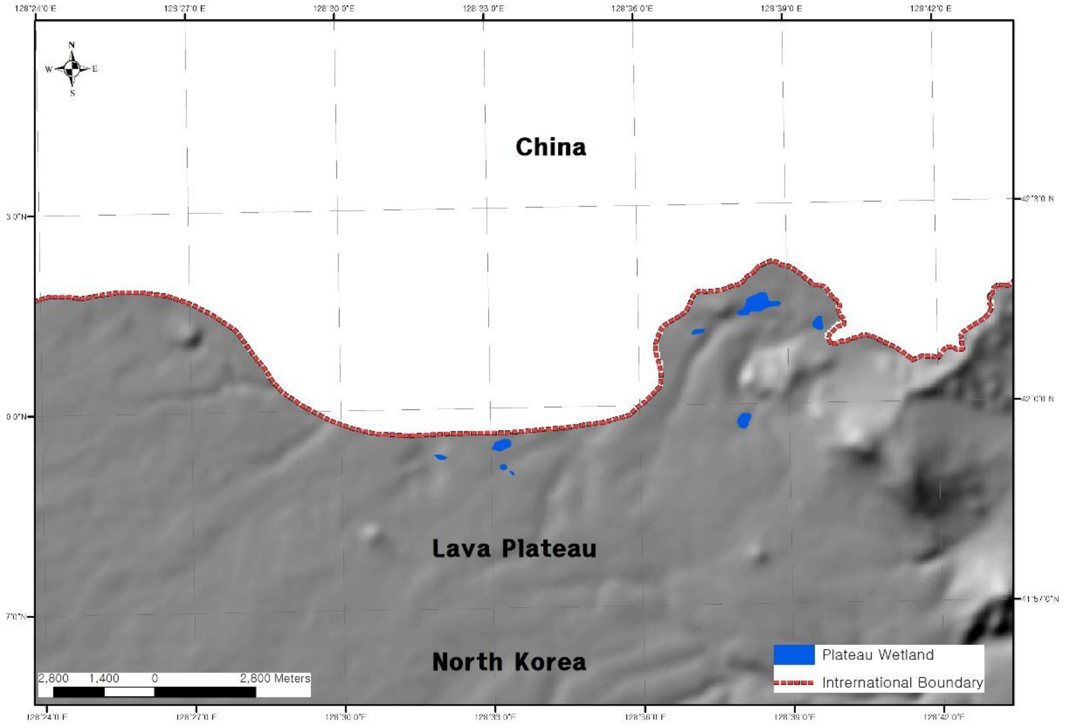

Category_4: Delta

Left:Nakdong river, Right: Duman river

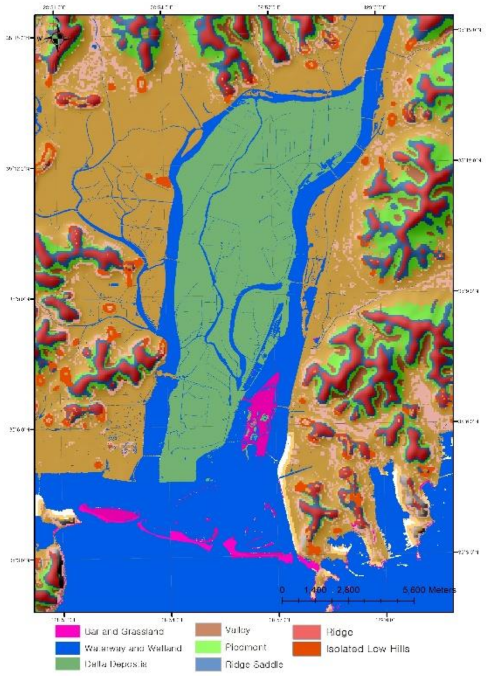

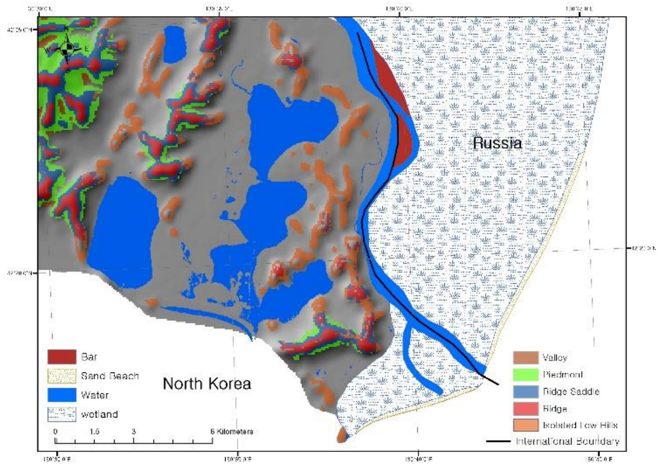

Habitats for biological Communites

6 Cateories, 461 Habitats

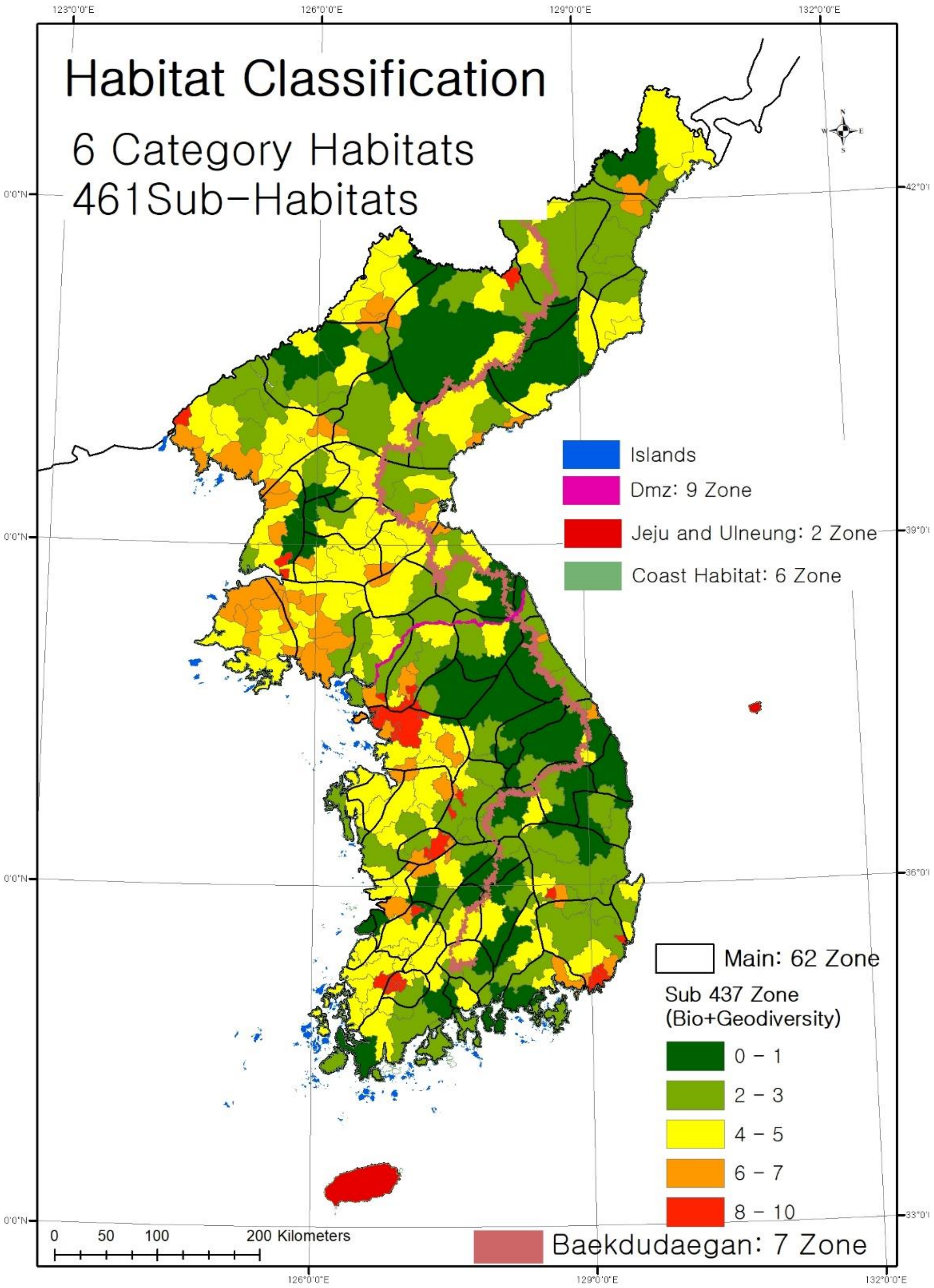

Supplementary data 4. Indicator species each habitats.

Indicator Species	Significance level	
Group 10 Indicator species 7	stat	p.value
Echinosophora koreensis (Nakai) Nakai	0.387	0.005 **
Epimedium koreanum Nakai	0.344	0.010 **
Sanicula rubriflora F.Schmidt ex Maxim.	0.338	0.005 **
Polygonatum sibiricum F. Delaroche	0.308	0.030 *
Neillia uekii Nakai	0.307	0.005 **
Sedum zokuriense Nakai	0.280	0.020 *
Paeonia obovata Maxim.	0.258	0.010 **
Group 12 Indicator species 8		
Berberis koreana Palib.	0.379	0.005 **
Mukdenia rossii (Oliv.) Koidz.	0.370	0.005 **
Forsythia saxatilis (Nakai) Nakai	0.356	0.030 *
Anemone amurensis KOM.	0.356	0.025 *
Viburnum burejaeticum	0.290	0.010 **
Aconitum pseudolaeve Nakai	0.258	0.015 *
Cirsium setidens (Dunn) Nakai	0.247	0.020 *
Clematis trichotoma Nakai	0.239	0.010 **
Group 13 Indicator species 1		
Prunus takesimensis Nakai	0.365	0.025 *
Group 15 Indicator species 6		
Lonicera chrysantha Turcz. ex Ledeb.	0.707	0.045 *
Lathyrus vaniotii H.Lév.	0.707	0.045 *
Spiraea trichocarpa Nakai	0.707	0.045 *
Oplopanax elatus	0.707	0.045 *
Corydalis maculata B.U.Oh & Y.S.Kim	0.295	0.040 *
Asarum versicolor (K.Yamaki) Y.N.Lee	0.277	0.030 *
Group 16 Indicator species 9		
Asplenium ruta-muraria L.	0.775	0.005 **
Pulsatilla tongkangensis Y. N. Lee & T. C. Lee	0.632	0.030 *
Saussurea chabyoungsanica H. T. Im	0.600	0.005 **
A. coreana	0.456	0.005 **
Viola mirabilis var. subglabra L.	0.447	0.025 *
Saxifraga octopetala Nakai	0.359	0.020 *
Anemone koraiensis Nakai	0.321	0.005 **
Hypodematium glanduloso-pilosum (Tagawa) Ohwi	0.308	0.030 *
Lonicera subsessilis Rehder	0.284	0.015 *
Group 18 Indicator species 1		
Ajuga spectabilis Nakai	0.232	0.045 *
Group 19 Indicator species 1		
Megaleranthis saniculifolia Ohwi	0.32	0.005 **

Group 20 Indicator species 1		
Clematis brachyura Maxim.	0.252	0.025 *
Group 22 Indicator species 1		
Anemone reflexa Steph. ex Willd.	0.319	0.005 **
Group 23 Indicator species 1		
Delphinium maackianum Regel	0.313	0.02 *
Group 24 Indicator species 5		
Artemisia rubripes Nakai	0.309	0.005 **
Hosta minor (Baker) Nakai	0.306	0.005 **
Gueldenstaedtia verna (Georgi) Boriss.	0.303	0.030 *
Vicia chosenensis Ohwi	0.279	0.005 **
Iris koreana Nakai	0.259	0.035 *
Group 28 Indicator species 1		
Aconitum austrokoreense Koidz.	0.455	0.005 **
Group 30 Indicator species 1		
Ribes komarovii Pojark.	0.333	0.03 *
Group 31 Indicator species 1		
Dracocephalum rupestre Hance	0.707	0.025 *
Group 33 Indicator species 6		
Filipendula formosa Nakai	0.452	0.005 **
allopia koreana B.U.Oh & J.G.Kim	0.433	0.005 **
Parasenecio pseudotaimingasa (Nakai) B.U.Oh	0.433	0.005 **
Smilacina bicolor Nakai	0.408	0.005 **
Crepidiastrum koidzumianum (Kitam.) Pak & Kawano	0.387	0.005 **
Abies koreana	0.333	0.005 **
Group 34 Indicator species 1		
Coreanomecon hylomeconoides Nakai	0.315	0.01 **
Group 35 Indicator species 79		
Sasa palmata (Bean) E.G.Camus	1.000	0.005 **
Peracarpa carnosa var. circaeoides (F.Schmidt ex Miq.) Makino	1.000	0.005 **
Angelica japonica A.Gray	1.000	0.005 **
Pternopetalum tanakae (Franch. & Sav.) Hand.-Mazz.	0.894	0.005 **
Adonis multiflora Nishikawa & Koki Ito	0.894	0.005 **
Galeola septentrionalis Rchb.f.	0.894	0.005 **
Chionographis japonica (Willd.) Maxim.	0.894	0.005 **
Maackia fauriei (H.Lév.) Takeda	0.894	0.005 **
Strobilanthes oliganthus Miq.	0.894	0.005 **
Damnacanthus indicus C.F.Gaertn.	0.894	0.005 **
Gentiana scabra Bunge	0.866	0.005 **
Artemisia japonica var. hallaisanensis (Nakai) Kitam.	0.866	0.005 **
Schisandra repanda (Siebold & Zucc.) Radlk.	0.866	0.005 **
Wedelia prostrata Hemsl.	0.816	0.005 **

Aruncus aethusifolius (H.Lev.) Nakai	0.816	0.005 **
Pteris cretica L.	0.756	0.005 **
Cirsium rhinoceros (H.Lev. & Vaniot) Nakai	0.756	0.005 **
Mercurialis leiocarpa Siebold & Zucc.	0.756	0.005 **
Rhynchospermum verticillatum Reinw.	0.756	0.005 **
Euphorbia pekinensis Rupr.	0.750	0.005 **
Dendranthema coreanum (H.Lev. & Vaniot) Vorosch.	0.707	0.015 *
Microlepia strigosa (Thunb.) C.Presl	0.707	0.005 **
Rhododendron weyrichii Maxim.	0.707	0.005 **
Daphne kiusiana Miq.	0.671	0.005 **
Aster arenarius (Kitam.) Nemoto	0.671	0.005 **
Desmodium caudatum (Thunb.) DC.	0.667	0.005 **
Pollia japonica Thunb.	0.632	0.005 **
Cimicifuga biternata (Siebold & Zucc.) Miq.	0.612	0.005 **
Cardamine glechomifolia H.Lev.	0.612	0.005 **
Ranunculus crucilobus H.Lev.	0.612	0.005 **
Ligustrum foliosum Nakai	0.577	0.005 **
Rhamnus taquetii (H.Lev. & Vaniot) H.Lev.	0.567	0.005 **
Cleyera japonica Thunb.	0.555	0.005 **
Ficus erecta var. sieboldii (Miq.) King	0.555	0.005 **
Wahlenbergia marginata (Thunb.) A.DC.	0.535	0.005 **
Distylium racemosum Siebold & Zucc.	0.535	0.005 **
Neolitsea aciculata (Blume) Koidz.	0.535	0.005 **
Zanthoxylum ailanthoides Siebold & Zucc.	0.516	0.005 **
Ardisia crenata Sims	0.516	0.005 **
Elaeagnus submacrophylla Servett.	0.516	0.005 **
Ligularia taquetii (H.Lev. & Vaniot) Nakai	0.500	0.030 *
Berberis amurensis var. quelpaertensis (Nakai) Nakai	0.474	0.005 **
Cimicifuga japonica (Thunb.) Spreng.	0.471	0.005 **
Ophiopogon jaburan (Siebold) Lodd.	0.471	0.005 **
Idesia polycarpa Maxim.	0.471	0.005 **
Verbena officinalis	0.459	0.005 **
Machilus japonica Siebold & Zucc.	0.459	0.005 **
Eurya emarginata (Thunb.) Makino	0.459	0.005 **
Actinodaphne lancifolia (Siebold & Zucc.) Meisn.	0.459	0.005 **
Corydalis decumbens (Thunb.) Pers.	0.447	0.005 **
Ainsliaea apiculata Sch.Bip.	0.447	0.005 **
Sageretia thea (Osbeck) M. C. Johnst.	0.436	0.005 **
Viburnum odoratissimum var. awabuki (K.Koch) Zabel ex Rumpler	0.436	0.005 **
Primula modesta var. hannasanensis T.Yamaz.	0.433	0.005 **
Quercus acuta Thunb.	0.417	0.005 **
Ostrya japonica Sarg.	0.401	0.010 **

Dryopteris fuscipes C.Chr.	0.400	0.005 **
Castanopsis sieboldii (Makino) Hatus.	0.400	0.005 **
Litsea japonica (Thunb.) Juss.	0.400	0.005 **
Rhaphiolepis indica var. umbellata (Thunb.) Ohashi	0.400	0.005 **
Diplopterygium glaucum (Thunb. ex Houtt.) Nakai	0.392	0.005 **
Caesalpinia decapetala (Roth) Alston	0.392	0.005 **
Quercus myrsinifolia Blume	0.392	0.005 **
Kadsura japonica (L.) Dunal	0.392	0.005 **
Cinnamomum yabunikkei H.Ohba	0.392	0.005 **
Quercus glauca	0.378	0.005 **
Aster hayatae H.Lev. & Vaniot	0.371	0.005 **
Messerschmidia sibirica	0.365	0.005 **
Elaeagnus glabra Thunb.	0.365	0.005 **
Boehmeria pannosa Nakai & Satake	0.365	0.005 **
Cnidium japonicum Miq.	0.365	0.005 **
Ficus erecta	0.348	0.005 **
Rubus hirsutus Thunb.	0.343	0.005 **
Ilex crenata Thunb.	0.338	0.005 **
Asarum maculatum Nakai	0.338	0.005 **
Pittosporum tobira (Thunb.) W.T.Aiton	0.320	0.005 **
Juncus setchuensis var. effusoides Buchenau	0.306	0.015 *
Artemisia dubia Wall.	0.270	0.010 **
Rhynchosia volubilis Lour.	0.263	0.030 *
Group 40 Indicator species 2		
Lycopodium clavatum var. nipponicum	0.500	0.015 *
Astilboides tabularis (Hemsl.) Engl.	0.385	0.010 **
Group 41 Indicator species 6		
Vaccinium bracteatum Thunb.	0.566	0.005 **
Callicarpa mollis Siebold & Zucc.	0.430	0.005 **
Indigofera kirilowii f. albiflora Uyeki	0.369	0.005 **
Mallotus japonicus (L.f.) Müll.Arg.	0.349	0.005 **
Bletilla striata (Thunb.) Rchb.f.	0.298	0.040 *
Millettia japonica (Siebold & Zucc.) A.Gray	0.296	0.035 *
Group 46 Indicator species 2		
Rubus ribisoideus Matsum.	0.333	0.03 *
Pinellia tripartita (Blume) Schott	0.320	0.04 *
Group 47 Indicator species 4		
Lespedeza maritima Nakai	0.311	0.010 **
Vicia hirticalycina Nakai	0.305	0.005 **
Ligustrum japonicum Thunb.	0.290	0.005 **
Deutzia paniculata Nakai	0.277	0.035 *
Group 49 Indicator species 1		

Goodyera velutina Maxim. ex Regel	0.816	0.005 **
Group 51 Indicator species 1		
Fimbristylis dichotoma (L.) Vahl	0.577	0.02 *
Group 54 Indicator species 1		
Scutellaria insignis Nakai	0.289	0.01 **
Group 56 Indicator species 1		
Dunbaria villosa (Thunb.) Makino	0.269	0.01 **
Group 57 Indicator species 24		
Veronica kiusiana var. diamantiaca (Nakai) T.Yamaz.	0.676	0.005 **
Androsace cortusaefolia Nakai	0.632	0.005 **
Bupleurum euphorbioides Nakai	0.539	0.005 **
Hanabusaya asiatica (Nakai) Nakai	0.527	0.005 **
Aristolochia manshuriensis Kom.	0.447	0.025 *
Patrinia rupestris (Pall.) Juss.	0.415	0.005 **
Acer ukurunduense Trautv. & C.A.Mey.	0.408	0.005 **
Rosa koreana Kom.	0.405	0.010 **
Forsythia ovata Nakai	0.405	0.005 **
Scabiosa tschiliensis Gruning	0.402	0.005 **
Menyanthes trifoliata	0.400	0.040 *
Leontopodium japonicum Miq.	0.390	0.005 **
Clematis fusca var. coreana (H.Lev.) Nakai	0.390	0.005 **
Salvia chanryoenica Nakai	0.378	0.005 **
Cardamine flexuosa	0.358	0.005 **
Acer tegmentosum	0.344	0.005 **
Lilium cernuum Kom.	0.341	0.005 **
Thalictrum rochebrunianum var. grandisepalum (H.Lév.) Nakai	0.327	0.005 **
Saxifraga punctata L.	0.316	0.030 *
Viola diamantiaca Nakai	0.307	0.005 **
Saussurea macrolepis (Nakai) Kitam.	0.253	0.010 **
Scopolia japonica Maxim.	0.250	0.020 *
Cirsium pendulum Fisch. ex DC.	0.244	0.035 *
Scrophularia koraiensis Nakai	0.224	0.045 *
Group 58 Indicator species 12		
Melampyrum roseum var. hirsutum Beauverd	0.707	0.005 **
Lentinus lepideus (Fr.)Fr.	0.707	0.005 **
A. rufinerve Nak.	0.707	0.005 **
Potentilla fruticosa var. mandshurica Maxim.	0.707	0.010 **
Betula fusenensis	0.707	0.005 **
Picea jezoensis (Siebold & Zucc.) Carrière	0.707	0.005 **
Betula microphylla var. coreana	0.707	0.005 **
Sorbus alnifolia (Siebold & Zucc.) K.Koch	0.612	0.005 **
Salix orthostemma	0.612	0.005 **

Cardamine trifida (Lam. ex Poir.)	0.577	0.010 **
Lycopodium alpinum L.	0.577	0.010 **
Euphrasia retrotricha	0.365	0.035 *
Group 59 Indicator species 12		
Dendropanax morbiferus H.Lév.	0.440	0.005 **
Rubus corchorifolius L.f.	0.371	0.005 **
Rhus succedanea L.	0.362	0.005 **
Nanocnide japonica Blume	0.360	0.005 **
Neolitsea sericea (Blume) Koidz.	0.352	0.005 **
Lindera sericea (Siebold & Zucc.) Blume	0.336	0.005 **
Pteris multifida Poir.	0.318	0.005 **
Rhus sylvestris Siebold & Zucc.	0.316	0.005 **
Meliosma myriantha Siebold & Zucc.	0.303	0.005 **
Chloranthus fortunei (A.Gray) Solms	0.296	0.005 **
Arisaema ringens (Thunb.) Schott	0.286	0.005 **
Hepatica insularis Nakai	0.285	0.005 **
Group 501 Indicator species 5		
Lycoris uydoensis M.Y.Kim	1.000	0.010 **
Hosta yingeri S.B.Jones	1.000	0.010 **
Euonymus chibai Makino	0.577	0.040 *
Dumasia truncata Siebold & Zucc.	0.577	0.015 *
Silene capitata Kom.	0.408	0.050 *

Supplementary data 5. Conservation grade of landform for each scale.

<table>
<tr><th>Category_1
1:5,000,000</th><th>Values</th><th>Category_2
1:1,000,000</th><th>Values</th><th>Category_3
1: 50,000 ~ 25,000</th><th>Values</th><th>Category_4
Under 1:5,000</th><th>Values</th></tr>
<tr><td rowspan="24">Mountains</td><td rowspan="24">Ⅰ,Ⅱ</td><td rowspan="6">High Elevation Mt.</td><td rowspan="6">Ⅰ</td><td rowspan="2">Mt. Granite</td><td rowspan="2">Ⅰ</td><td>Magma Plateau</td><td>Ⅰ</td></tr>
<tr><td rowspan="2">Highland Flat</td><td rowspan="2">Ⅰ</td></tr>
<tr><td rowspan="2">Mt. Gneiss Series</td><td rowspan="2">Ⅰ</td></tr>
<tr><td rowspan="2">Karst Basin</td><td rowspan="2">Ⅰ</td></tr>
<tr><td rowspan="3">Mt. Tertiary Layer</td><td rowspan="2">Ⅱ</td></tr>
<tr><td rowspan="3">Karst Flat</td><td rowspan="3">Ⅰ</td></tr>
<tr><td rowspan="8">Middle Elevation Mt.</td><td rowspan="9">Ⅰ</td><td rowspan="3">Ⅰ</td></tr>
<tr><td rowspan="2">Mt. Limestone</td></tr>
<tr><td rowspan="2">Wetland Mt.</td><td rowspan="2">Ⅰ</td></tr>
<tr><td rowspan="2">Mt. Pyroclast</td><td rowspan="3">Ⅰ</td></tr>
<tr><td rowspan="2">Wind Hole</td><td rowspan="2">Ⅰ</td></tr>
<tr><td rowspan="4">Volcanic Mt.</td></tr>
<tr><td rowspan="3">Ⅰ</td><td>Piedmont</td><td>Ⅱ (landuse Ⅲ)</td></tr>
<tr><td rowspan="3">Ridge</td><td rowspan="3">Ⅰ</td></tr>
<tr><td rowspan="7">Piedmont</td></tr>
<tr><td rowspan="6">Ⅱ (landuse Ⅲ)</td><td rowspan="2">Lava Plateau</td><td rowspan="2">Ⅰ</td></tr>
<tr><td rowspan="2">Valley</td><td rowspan="2">Ⅰ</td></tr>
<tr><td rowspan="2">High Flat</td><td rowspan="2">Ⅰ</td></tr>
<tr><td rowspan="2">Ridge Saddle</td><td rowspan="2">Ⅰ</td></tr>
<tr><td rowspan="3">Limestone Basin</td><td rowspan="4">Ⅰ</td></tr>
<tr><td rowspan="3">Rock Block</td><td rowspan="3">Ⅰ</td></tr>
<tr><td rowspan="3">Drainage Divide</td><td rowspan="3">Ⅰ</td></tr>
<tr><td rowspan="2">Isolated Mountain</td></tr>
<tr><td>Ⅰ</td><td>Mountain</td><td>Ⅰ</td></tr>
</table>

				Drainage Divide		Cliff	
					Ⅰ	Crator	Ⅰ
				Water and Lake	Ⅰ,Ⅲ	Mountain Bog	Ⅰ
		Water and Lake	Ⅰ	Wetland in Lake	Ⅰ	Doline Wetland	Ⅰ
				Inland Erosion Basin	Ⅲ	Drainage Divide	Ⅰ
				Caldera Basin	Ⅰ	Water and Lake	Ⅰ,Ⅲ
						Wetland in Lake	Ⅰ
Plains	Ⅱ, Ⅲ	Alluvial Plains	Ⅲ	Coastal Plains	Ⅲ	Bar and Grassland	Ⅱ
						Waterway and Wetland	Ⅱ
				Inland Plains	Ⅲ	Small irrigation pond and Canal	Ⅱ
		Undulating Hills	Ⅱ (landcover Ⅲ)	Delta Plains	Ⅱ	Small Inland Plains	Ⅲ
						Small Coastal Plains	Ⅲ
				Water and Lake	Ⅱ	Inland Wetland	Ⅰ
						Isolated Low Hills	Ⅱ
				Wetland in Lake	Ⅰ	Low Relief Gentle Slope	Ⅲ
						Paddy Field Wetland	Ⅲ
				Low Gentle	Ⅲ	Water and Lake	Ⅱ,Ⅲ

				Slope		Wetland in Lake	Ⅱ
Fluvial Landform	Ⅰ, Ⅱ	Incised Meander Stream	Ⅰ	Rocky Channel	Ⅰ	River Terrace	Ⅱ
						Alluvial Island	Ⅲ
				Sand and Gravel Channel	Ⅰ	Riparian Wetland	Ⅱ
						Riverside Wetland	Ⅱ
				Silt and Mud Channel	Ⅱ,Ⅲ	Braided Stream Channel	Ⅱ
		Meander Stream	Ⅱ			Bar	Ⅱ
				Water and Lake	Ⅱ	Riverside Land	Ⅱ
						Stream and Lake	Ⅱ,Ⅲ
				Wetland in Lake	Ⅰ	Wetland in Lake	Ⅱ
						Fluvial Cliff	Ⅰ
Coastal Landform	Ⅰ, Ⅱ,Ⅲ	Uplift Coast	Ⅰ	Rocky Coast	Ⅰ	Coastal Terrace	Ⅰ
						Rocky Beach	Ⅰ
						Sand Beach	Ⅰ
				Sand Coast	Ⅰ	Sand and Mud Beach	Ⅱ
						Sand and Gravel Beach	Ⅰ
				Sand and Mud Coast	Ⅱ	Tidalflat	Ⅰ
		Rias Coast	Ⅰ (landc over Ⅱ,Ⅲ)			Mixed Coast	Ⅱ
				Sand and Gravel Coast	Ⅱ	Sandune Wetland	Ⅰ
						Sand Dune	Ⅰ
				Mud Coast	Ⅰ	Salt Marsh	Ⅰ
						Lagoon	Ⅰ

		Volcanic Coast	Ⅱ(land cover Ⅲ)				
						Coastal Cliff	Ⅰ
				Mixed Coast	Ⅱ	Headland	Ⅱ
Island	Ⅰ	Island	Ⅰ (landcoer Ⅲ)	Island	Ⅰ (landcoer Ⅲ)	Mud Beach	Ⅰ
						Salt Marsh	Ⅰ
						Sand Beach	Ⅰ
						Sanddune	Ⅰ
		Volcanic Island	Ⅰ (landcoer Ⅲ)	Volcanic Island	Ⅰ (landcoer Ⅲ)	Lagoon	Ⅰ
						Tidal Flat	Ⅰ
						Cliff	Ⅰ
						Coastal Terrace	Ⅰ
Baekdudaegan	Ⅰ	Baekdudaegan	Ⅰ	Baekdudaegan	Ⅰ	Baekdudaegan Ecozone	Ⅰ
DMZ	Ⅰ	DMZ	Ⅰ	DMZ	Ⅰ	DMZ Ecozone	Ⅰ
